# Multi-hazard exposure and vulnerability drive rising agricultural risk in global breadbasket regions

**DOI:** 10.1038/s41467-026-74617-5

**Published:** 2026-06-18

**Authors:** Hossein Tabari, Parisa Hosseinzadehtalaei

**Affiliations:** 1 Faculty of Applied Engineering, University of Antwerp, Antwerp, Belgium; 2 Department of Meteorological and Climate Research, Royal Meteorological Institute of Belgium, Uccle, Belgium; 3 United Nations University Institute for Water, Environment and Health (UNU-INWEH), Richmond Hill, Ontario Canada; 4 Department of Physics and Astronomy, Ghent University, Ghent, Belgium; 5 Sino-Belgian Joint Laboratory for Geo-Information, Ghent, Belgium

**Keywords:** Natural hazards, Climate change, Hydrology, Agriculture

## Abstract

Climate extremes increasingly threaten global food production, yet global multi-hazard agricultural risk assessments that jointly consider multiple hazards, crop exposure and socioeconomic vulnerability are still missing. This gap limits our ability to identify where climate shocks to croplands are most likely to translate into high agricultural risk. Here we present a global agricultural risk assessment that quantifies the exposure of croplands and ten major crop groups to heatwaves, droughts, floods and compound hot–dry events, and combines this with a vulnerability index constructed from socio-economic and institutional indicators using latent factor modelling. Using ensembles of climate and hydrological models under multiple future scenarios, we show widespread increases in multi-hazard exposure, with the strongest intensification across South Asia, Sub-Saharan Africa and major U.S. and European breadbaskets. A significant relationship between vulnerability and cropland exposure emerges in future scenarios but is absent historically, indicating a growing role for vulnerability in shaping future agricultural risk. These results demonstrate that reliably identifying future agricultural risk hotspots requires jointly accounting for multi-hazard exposure and socioeconomic vulnerability, so that adaptation efforts can be targeted to regions where intensifying extremes coincide with high vulnerability.

## Abstract (web summary)

Climate extremes increasingly threaten global food production. This study shows that cropland exposure to multiple hazards is rising worldwide, especially in major breadbasket regions, with vulnerability amplifying future agricultural risk.

## Introduction

The resilience of the global food system depends on a small number of highly productive regions, or breadbaskets, that supply most of the world’s staple crops, including rice, wheat, maize, and soy^1,2^. Together, the United States, Argentina, Europe, Russia/Ukraine, China, India, Australia, Indonesia, and Brazil produced 56%, 56%, 73%, and 38% of global wheat, maize, soybean, and rice production, respectively in 2012^1^, while occupying about half of the world’s cropland^3,4^. These regions are increasingly threatened by anthropogenic climate change, which amplifies the frequency, severity, and spatial extent of extremes such as heatwaves, droughts, and floods^5–8^. Climate variability already accounts for nearly half of global year-to-year yield fluctuations over the past several decades^9,10^, and the intensification of extremes raises the risk of simultaneous production shocks that could destabilize markets and food access^11,12^.

Over the past decade, major advances have been made in quantifying the impacts of gradual warming and shifting climate patterns on agricultural productivity. Process-based crop models have been used to project yields under different global warming levels^13^, rising atmospheric CO_2_^14^, and regional climate conditions^15–19^. Global multi-model ensembles now estimate mean yield responses for major crops under various emission scenarios^20^. Similarly, empirical approaches link historical crop yields to observed climate anomalies^1,5,21–25^ or explore adaptation measures such as adjusting sowing dates and cultivar maturity classes^26^, as well as breeding advances^27,28^. These studies have provided valuable insights into long-term changes in crop productivity under gradual climate forcing.

Yet major gaps remain. Most global process-based assessments of agricultural impacts of climate change^13,14,20,22,29–31^ focus on mean temperature, precipitation, and CO_2_ trends or on simple anomalies rather than explicitly quantifying climate extremes (floods, droughts, and heatwaves) that directly drive agricultural losses. In addition, global empirical and statistical analyses typically consider only one hazard at a time^32–34^, and future multi-hazard assessments, especially those that include compound events across diverse crop types, are still missing. The absence of such a framework limits understanding of how different hazards and their concurrent or sequential interactions, such as compound hot–dry events, exacerbate agricultural impacts^4,23,35^. Moreover, global crop risk analyses rarely include vulnerability, despite evidence that socioeconomic and institutional conditions strongly modulate impacts^36,37^. As a result, true global agricultural risk hotspots, where high exposure coincides with limited adaptive capacity, remain poorly identified.

Here, we address these gaps by developing a global risk assessment of agricultural crops that integrates multi-hazard exposure and socioeconomic vulnerability. We quantify the exposure of total cropland and ten major crop groups to four key hazards (heatwaves, droughts, floods, and compound hot–dry events) under multiple future socioeconomic and land-use scenarios. The projected cropland exposure is compared among farming regions (breadbaskets, intermediate, and limited farming zones, defined by their cropland fraction). We then develop a vulnerability index using unsupervised latent factor modeling that objectively weights and synthesizes multidimensional demographic, development, and governance indicators into interpretable resilience dimensions (see Methods). By integrating this index with cropland exposure to climate extremes, we assess the risk of farming regions to future climate shocks. Our results show that breadbasket regions are projected to experience the largest increases in cropland exposure to climate extremes, with particularly strong signals in South Asia, Sub-Saharan Africa, and the United States. Differences across farming regions are driven more by climate change than by changes in cropland area. A statistically significant relationship between vulnerability and cropland exposure emerges in future scenarios but is absent historically, indicating a growing role for vulnerability in shaping future agricultural risk.

## Results

### Multi-hazard exposure of croplands

We assessed future changes in global cropland exposure to four types of climate extremes (floods, heatwaves, droughts, and compound hot-dry events) defined as the product of the cropland fraction and the probability of each event occurring at the grid-cell level (see “Methods”). This assessment was performed across a range of Shared Socioeconomic Pathways (SSPs), which represent plausible trajectories of global demographic, economic, and technological development, as well as environmental governance^38^.

**Fig. 1:**
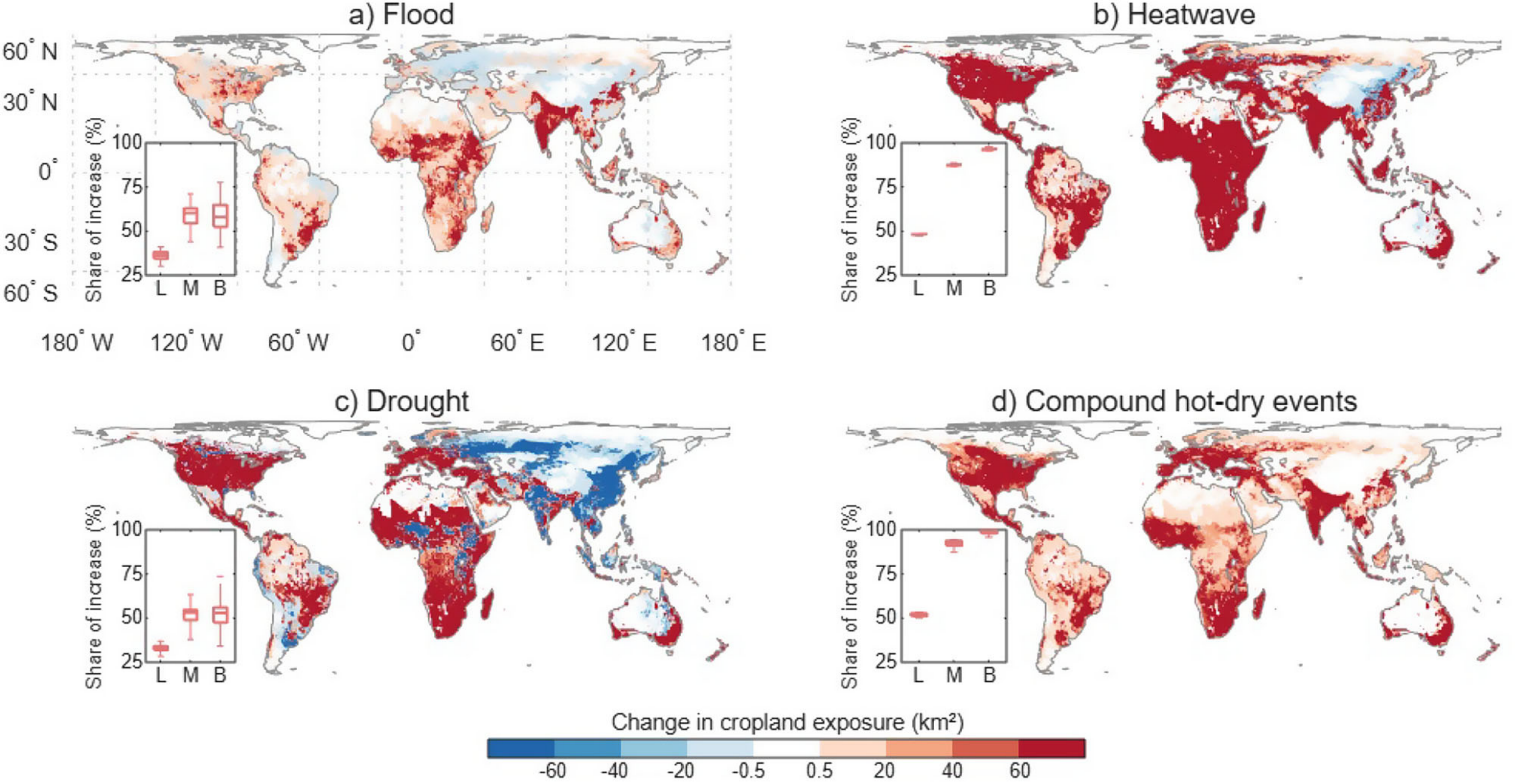
Projected changes in global cropland exposure to climate extremes by the end of the 21st century under SSP5-8.5. The maps show the absolute change (km²) in global cropland area exposed to (**a**) floods, (**b**) heatwaves, (**c**) droughts, and (**d**) compound hot-dry events between the late twentieth century and the late twenty-first century, based on the multi-model ensemble median. Cropland exposure is defined as the product of a climate-driven hazard and cropland proportion. For floods, the hazard is taken from RCP-forced ISIMIP2b simulations and combined with SSP-based cropland, whereas for the other extremes, both hazard and land use are SSP-based. Insets in each panel summarize the spatial coverage of increasing exposure (“Share of increase (%)”) across three farming regions: limited (L), intermediate (M) and breadbasket (B). Boxplots represent distributions across independent model ensemble members, with the center line indicating the median, boxes the interquartile range (25th–75th percentiles), and whiskers extending to the most extreme values within 1.5×IQR. Sample sizes are *n*  =  28 (floods), 18 (heatwaves), 22 (droughts), and 22 (compound events). Results for SSP1-2.6 are shown in Supplementary Fig. 1.

Under the most intense scenario, about 72% of global croplands are projected to experience increasing flood exposure by the end of the 21st century (Fig. 1). The largest increases occur in low-lying, intensively farmed regions of South and Southeast Asia, particularly the Ganges–Brahmaputra and Mekong basins (Fig. 1), consistent with the projected intensification of extreme precipitation in these monsoon-dominated regions^39^. Additional hotspots include parts of Central Africa and South America. Our results extend historical evidence of increasing cropland exposure to river flooding^40^ into future climate scenarios and show that regions identified as historical flood exposure hotspots, particularly in South and East Asia^41^, also experience some of the largest projected increases.

Heatwaves show a widespread intensification, with 85% of global croplands experiencing increased exposure. Particularly strong increases occur in the United States (Midwest and California), Southern and Eastern Europe, India, China’s North Plain, and parts of Brazil and Sub-Saharan Africa. These regions see the largest expansion in cropland affected by persistent extreme heat, reflecting the consistent increase in mean temperatures and heat extremes projected across climate models^42^.

For droughts, 53% of global croplands show increasing exposure under SSP5-8.5. The Mediterranean Basin, western North America, northeastern Brazil, and southern Africa emerge as major hotspots. These areas experience notable drying trends, reduced soil moisture, and increasing water stress^43^, posing critical challenges to both irrigated and rainfed agriculture. The pronounced increase in drought hazard and exposure in major breadbasket regions, including Brazil, aligns with earlier global assessments^43–45^, although the magnitude differs due to the use of different models and methodological approaches^46^.

The spatial pattern of compound hot-dry events reflects the combined influence of both drought and heatwave patterns, with 91% of global croplands showing increasing exposure. Hotspots occur where both heat and moisture deficits intensify simultaneously, including Southern Europe, the Sahel, India, and much of the United States. These compound extremes are particularly concerning due to their synergistic impacts on crop stress, often leading to yield failures and increased food insecurity^47,48^.

These projections underscore a growing and geographically widespread threat to global agricultural systems, with several major crop-producing regions simultaneously facing heightened exposure to multiple climate hazards. The stark contrast with the lower exposure increases under the low-emission scenario SSP1-2.6, where 54%, 63%, 43%, and 79% of global cropland area are projected to experience increasing exposure to floods, heatwaves, droughts, and compound hot-dry events, respectively (Suppl. Fig. 1), underscores the critical importance of ambitious climate mitigation in safeguarding food production. Consistent with this, a recent study showed that under SSP1-2.6 the global cropland gross primary productivity (GPP) exposure is reduced by 330% relative to SSP5-8.5 by the end of the 21st century^49^, indicating a substantially lower exposure of cropland productivity to climate extremes under a low-emission pathway.

Beyond global and regional aggregates, our gridded analysis (Fig. 1 and Supplementary Fig. 1) reveals pronounced subnational contrasts within major agricultural producers. The Indo-Gangetic Plain, spanning northern India, southern Nepal, eastern Pakistan, and Bangladesh, consistently emerges as a multi-hazard hotspot, with cropland highly exposed to floods, droughts, heatwaves, and compound hot-dry events. In the United States, elevated exposure to heatwaves, droughts, and compound events is concentrated across the Midwest and southern Great Plains. In Brazil, exposure increases most sharply in the southern states (Paraná, Rio Grande do Sul, and Santa Catarina), where cropland faces rising risks from all four extremes, whereas northern agricultural frontiers show an intensification of heat and compound events. These patterns demonstrate that exposure is far from spatially uniform, even within top crop-producing nations.

### Regional disparities in cropland exposure

**Fig. 2:**
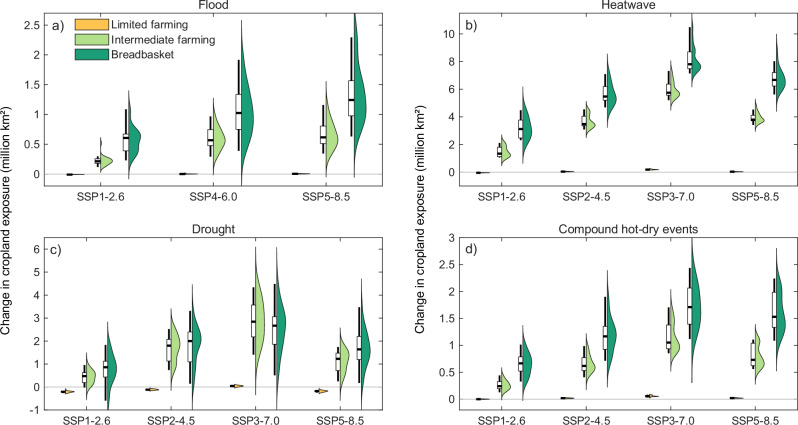
Comparison of projected changes in cropland exposure to climate extremes among farming regions. Cropland exposure was defined as the product of a climate-driven hazard and cropland proportion, and changes are calculated as differences between the late twentieth century and the late twenty-first century. For floods, the hazard is taken from RCP-forced ISIMIP2b simulations and combined with SSP-based cropland, whereas for the other extremes both hazard and land use are SSP-based. Violin plots depict the ensemble probability density for (**a**) flood, (**b**) heatwave, (**c**) drought, and (**d**) compound events. Boxplots show the ensemble median (horizontal line), interquartile range (25th–75th percentiles; box), and whiskers extending to the most extreme values within 1.5×IQR; outliers (if present) are shown as points. Sample sizes are *n*  =  28 (floods), 18 (heatwaves), 22 (droughts), and 22 (compound events). For the spatial distribution of farming regions under historical and SSP scenarios, see Suppl. Fig. 2.

To better understand the spatial distribution of future climate risks to agriculture, we compared projected changes in cropland exposure to climate extremes across three types of farming regions: limited farming, intermediate farming, and breadbasket regions, as defined under historical and future SSP scenarios, thereby accounting for the evolving dynamics of breadbasket regions (Suppl. Fig. 2). A consistent and striking pattern emerges: breadbasket regions are projected to experience the largest increases in cropland exposure across all extreme event types (floods, heatwaves, droughts, and compound hot-dry events) particularly under the high-emission SSP3-7.0 and SSP5-8.5 scenarios (Fig. 2).

Across all extremes and scenarios, projected changes for limited farming regions are almost zero or negative, whereas the ensemble medians and upper distribution tails of cropland exposure are substantially higher in breadbasket regions than in intermediate regions (Fig. 2). Breadbasket regions are expected to be most affected by heatwaves, with cropland exposure increases of 2.4, 5.6, 8.9, and 6.2 million km² projected for SSP1-2.6, SSP2-4.5, SSP3-7.0, and SSP5-8.5, respectively. This is followed by drought, with corresponding increases of 0.6, 2.4, 3.7, and 1.7 million km², and by compound hot-dry events, with increases of 0.5, 1.1, 1.7, and 1.4 million km² for SSP1-2.6, SSP2-4.5, SSP3-7.0, and SSP5-8.5, respectively. For floods, the projected increases in cropland exposure are 0.3, 0.7, and 0.8 million km² for SSP1-2.6, SSP4-6.0, and SSP5-8.5, respectively. In terms of spatial coverage (Fig. 1), a large fraction of breadbasket regions is projected to experience increasing cropland exposure: 59% ( ± 7% uncertainty across models), 94% ( ± 0.6%), 53% ( ± 8%), and 98% ( ± 1.1%) of breadbasket croplands show rising exposure to floods, heatwaves, droughts, and compound hot-dry events, respectively.

Although exposure in breadbasket regions is highest, intermediate farming regions also exhibit substantial increases in cropland exposure across all extremes and scenarios. These regions often act as transitional production zones and potential buffers within global food supply networks, and may therefore play a critical role in absorbing future production shocks and maintaining food system stability. For example, under SSP5-8.5, median exposure in intermediate regions increases by 2.5 million km² for heatwaves, 0.6 million km² for drought, 0.5 million km² for compound hot–dry events, and 0.3 million km² for floods, representing roughly 34–40% of the corresponding increases observed in breadbasket regions. In terms of spatial coverage (Fig. 1), 62% ( ± 4% uncertainty across models), 86% ( ± 0.4%), 57% ( ± 6%), and 91% ( ± 1.8%) of intermediate farming regions are projected to experience increasing cropland exposure to floods, heatwaves, droughts, and compound hot-dry events, respectively.

Given that breadbasket regions concentrate the highest absolute agricultural risk while supplying a disproportionate share of global crop and food production, the contrast across farming zones remains decisive. For all types of extremes, the disparity in cropland exposure between farming regions is smallest under SSP1-2.6 and largest under SSP3-7.0 and SSP5-8.5, highlighting the reinforcing effects of emission trajectories on regional inequality in climate risk. Notably, breadbasket regions not only face higher exposure in absolute terms but also exhibit greater inter-model spread, reflecting both intensified and more uncertain risks under future climate conditions. Importantly, the inter-model spread increases from limited to intermediate to breadbasket regions (Fig. 2), indicating higher uncertainty in areas with greater projected exposure. For drought, heatwave, and compound hot–dry events, uncertainty mainly stems from differences in climate inputs across GCMs. For floods, in addition to climate-model differences, uncertainty arises from structural heterogeneity among global hydrological models. This ensemble spread is therefore a key component of projection uncertainty and highlights that regional exposure estimates should be interpreted as probabilistic rather than deterministic.

### Crop-specific exposure to climate extremes

**Fig. 3:**
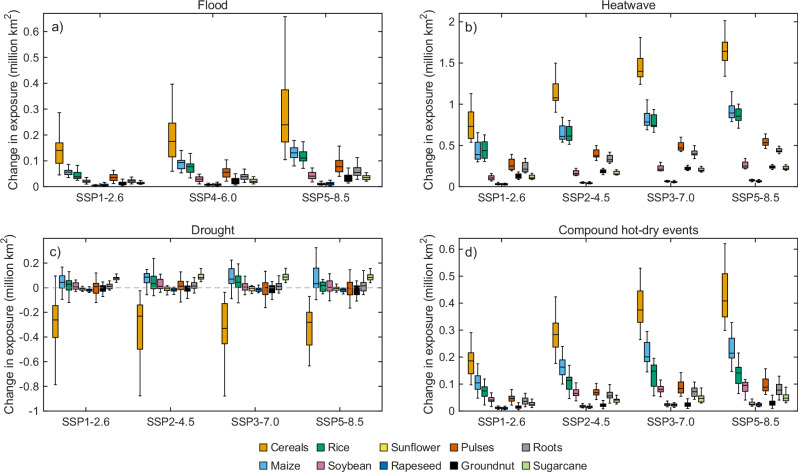
Comparison of projected changes in global exposure of different crops to climate extremes. Box-and-whisker plots show the distribution of projected changes in exposure to (**a**) floods, (**b**) heatwaves, (**c**) droughts, and (**d**) compound hot–dry events across climate model ensembles for major crop types under different SSP scenarios. The center line represents the median, boxes indicate the interquartile range (25th–75th percentiles), and whiskers extend to the most extreme non-outlier values within 1.5 times the interquartile range. Outliers are not shown. Changes are calculated by comparing the late 20th century and the late 21st century. Sample sizes are *n*  =  28 for floods, 18 for heatwaves, 22 for droughts, and 22 for compound events.

To evaluate the differential vulnerability of global food systems, we assessed how projected exposure to climate extremes varies across ten dominant crop species and groups (cereals, maize, rice, soybean, sunflower, rapeseed, pulses, groundnut, roots, and sugarcane) by the end of the 21st century under different SSP scenarios (Fig. 3). These crops together account for the majority of global food production, making their exposure critical for understanding climate risks to food security. Cereals show the highest absolute increases in exposure to all extremes except drought, with the most pronounced changes occurring under high-emission scenarios SSP3-7.0, SSP4-6.0, and SSP5-8.5. The inter-model spread (error bars) indicates moderate to large uncertainty, particularly for heatwave and flood exposure.

Heatwaves drive the largest increases, with cereal exposure expanding by 0.7–1.6 million km² ( ± 0.2 million km² uncertainty) depending on the scenario (Fig. 3). Among individual crops, maize and rice are consistently the most exposed to climate extremes, particularly to heatwaves, 0.4–0.9 million km² ( ± 0.1 million km² uncertainty), followed by compound hot-dry events, floods, and droughts. However, sugarcane stands out with higher exposure to drought. Most crop types follow a similar exposure ranking: heatwaves > compound events > floods > droughts.

**Fig. 4:**
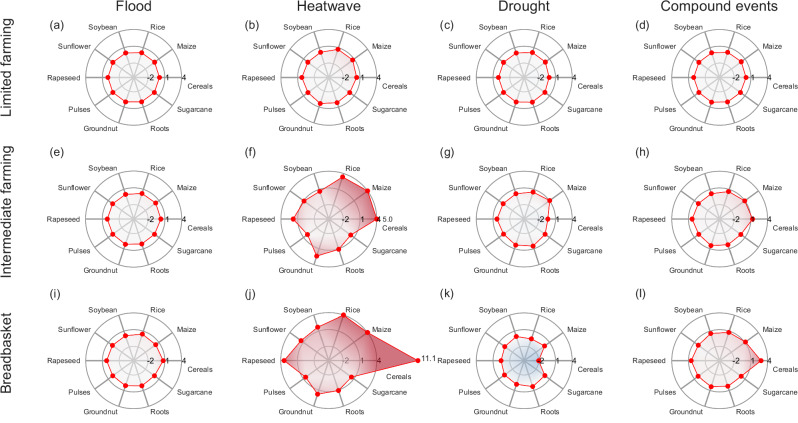
Comparison of projected changes in global cropland exposure of different crops to climate extremes across farming regions for SSP5-8.5. Shown are differences in cropland exposure (× 10⁵ km²) between the late 20th century and the late 21st century for major crops under flood (**a**, **e**, **i**), heatwave (**b**, **f**, **j**), drought (**c**, **g**, **k**), and compound events (**d**, **h**, **l**) across limited farming (**a**–**d**), intermediate farming (**e**–**h**), and breadbasket regions (**i**–**l**). Values along the axes represent exposure changes for individual crops, and concentric circles indicate increasing levels of exposure change. Results for other scenarios are provided in Supplementary Figs. 3–6.

Comparing crop-specific exposure across regions reveals that the increase in exposure to floods, heatwaves, and compound hot-dry events is consistently larger in breadbasket regions than in intermediate and limited farming regions under all future scenarios (Fig. 4 and Supplementary Figs. 3–6). This disparity is especially pronounced for cereals, rice, and maize. For instance, under SSP3-7.0, the projected increase in cropland exposure to heatwaves in breadbasket regions exceeds that in intermediate regions by 0.9, 0.4, and 0.4 million km² for cereals, rice, and maize, respectively. These inter-regional differences in exposure are more substantial under more intensive scenarios: SSP5 > SSP4 > SSP1 for floods, and SSP3 > SSP5 > SSP2 > SSP1 for heatwaves and compound events. A different pattern emerges for drought, where both increases and decreases in crop exposure are projected for different crops. Increases in exposure are generally more pronounced in intermediate farming regions across most crop types. However, under SSP3-7.0, for globally dominant crops such as rice, maize, and soybean, breadbasket regions show larger drought exposure than the other two regions.

### Driver of regional disparities in cropland exposure

**Fig. 5:**
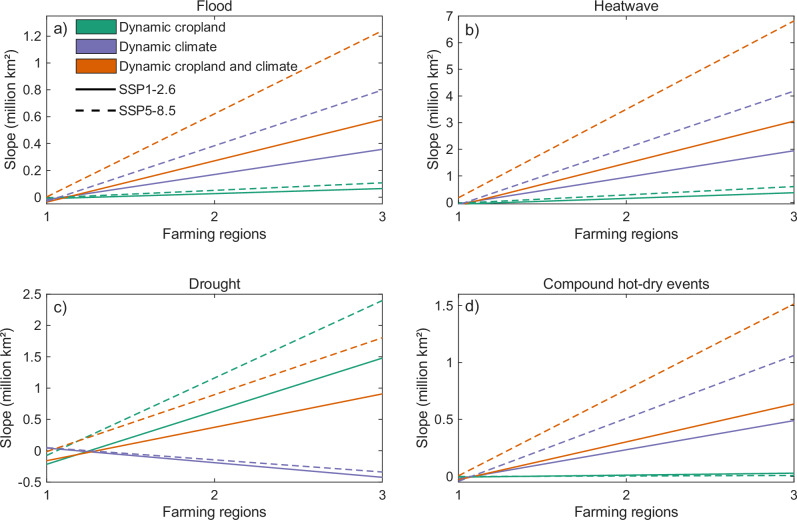
Drivers of differences in projected changes in cropland exposure among farming regions. Plots show the slope of projected changes in cropland exposure to flood (**a**), heatwave (**b**), drought (**c**), and compound events (**d**) across farming regions under three conditions: dynamic cropland, dynamic climate, and both dynamic cropland and dynamic climate, for SSP1-2.6 and SSP5-8.5. A steeper slope indicates a larger difference in projected exposure among farming regions. Changes are calculated by comparing the late 20th century and the late 21st century.

To disentangle the relative contributions of climate change and cropland dynamics to regional disparities in cropland exposure, we analyzed three scenarios: (1) static climate (historical) with dynamic cropland (future), (2) dynamic climate (future) with static cropland (historical), and (3) both dynamic climate and dynamic cropland (future). For floods, heatwaves, and compound hot-dry events, differences in cropland exposure across farming regions are driven far more by climate change than by changes in cropland extent (Fig. 5 and Suppl. Fig. 7). Although exposure increases progressively from limited to intermediate and breadbasket regions under all scenarios, the slope of change is considerably steeper under dynamic climate than under dynamic cropland conditions, especially under SSP1 and SSP5. In contrast, for drought, cropland dynamics play a more influential role. Under a dynamic climate, the slope of cropland exposure change across regions is negative, whereas under dynamic cropland it is positive, indicating that cropland changes, rather than climate change, account for the increasing exposure disparities. The positive slope under dynamic cropland for drought is 3–12 times larger than the negative slope under dynamic climate, resulting in an overall positive combined effect. This implies that, similar to the other extreme types, exposure to drought also intensifies from limited to intermediate and breadbasket regions when both climate change and cropland dynamics are considered. These findings underscore the need to jointly consider climate and land-use trajectories when evaluating regional patterns of agricultural risk.

### Vulnerability and adaptive capacity to increasing cropland exposure

It is important to assess the vulnerability of countries regarding cropland exposure because exposure alone does not determine agricultural risk; vulnerability plays a central role in shaping how countries experience and manage climate-related impacts on agriculture. Cropland exposed to extreme events may contribute only modestly to increases in agricultural risk where vulnerability is low, because impacts can be more effectively absorbed and managed. Conversely, even moderate exposure can lead to marked increases in agricultural risk when vulnerability is high. To capture this, we assessed vulnerability using Principal Component Factor Analysis (PCFA; see Methods), based on six indicators: governance indicator (GI), Human Development Index (HDI), population size, gender ratio, tertiary education attainment, and age dependency ratio. This provides a multidimensional understanding of national capacities to cope with climate risks affecting cropland and to safeguard food security.

To assess the robustness, we benchmarked the PCFA vulnerability index against the Notre Dame Global Adaptation Initiative (ND-GAIN), a widely used and comprehensive composite of socioeconomic vulnerability that lacks future projections. Compared with the ND-GAIN food-sector vulnerability, spatial patterns align across countries (Supplementary Fig. S9a, b) and a strong linear association emerges in the country-by-country comparison (*r* = 0.881, *p* < 0.001), with most countries near the 1:1 line (Suppl. Fig. S9c). A Bland–Altman analysis shows differences cantered near zero and bounded within ±1.96 SD with no trend versus the mean (Suppl. Fig. S9d), indicating no systematic bias. To test generalizability beyond the food sector, we compare the PCFA index with overall ND-GAIN vulnerability (36 indicators across six sectors); agreement remains high in both spatial patterns and cross-country correlations (Suppl. Fig. S10), with similarly small, unbiased differences in Bland–Altman space. Consistency with both sector-specific and overall ND-GAIN indices indicates that the PCFA index captures core structural dimensions of socioeconomic vulnerability and supports its use for future projections.

To understand the role of vulnerability, we analyzed the relationship between the cropland exposure and vulnerability for different climate extremes and scenarios (Suppl. Fig. 8). The results reveal a strong relationship between vulnerability and exposure across all future scenarios and extreme event types. These relationships are statistically significant in all cases, except for SSP5 under drought conditions and SSP5 under compound events. In contrast, no such relationship is observed during the historical period. This suggests that, in future scenarios, countries with high cropland exposure also have high vulnerability, resulting in elevated agricultural risk. In particular, several major breadbasket regions face heightened risk, underpinned by persistently high vulnerability. When comparing SSPs, the relationship is weaker in SSP1 and SSP5, which are associated with higher investment in sustainable development and rapid economic growth, respectively^50^, while it is stronger in SSP2, SSP3, and SSP4, reflecting more unequal or fragmented development trajectories.

**Fig. 6:**
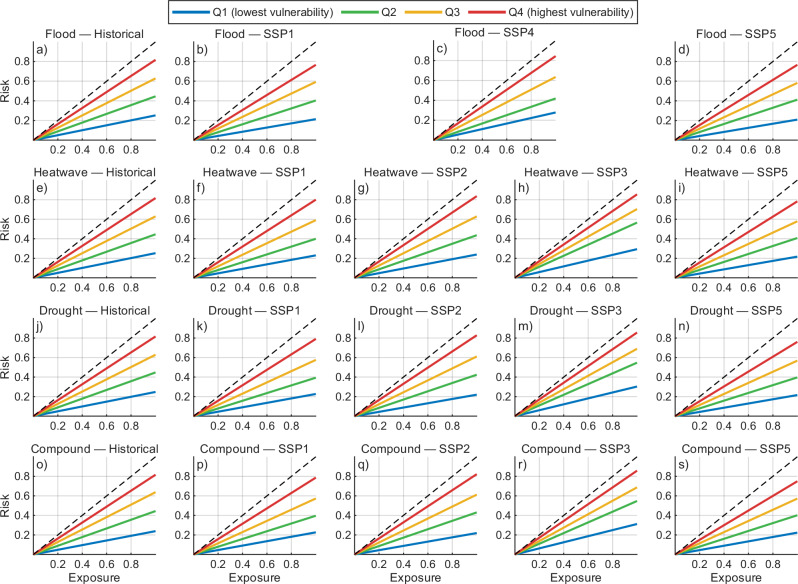
Exposure–risk relationships by vulnerability quartiles across hazards and SSP scenarios. Each panel shows the relationship between normalized cropland exposure and resulting risk for four vulnerability quartiles (Q1–Q4; colored lines) across 151 countries. Panels (**a**–**d**) depict flood, (**e**–**i**) heatwave, (**j**–**n**) drought, and (**o**–**s**) compound hot–dry events. The dashed line indicates the 1:1 reference where risk = hazard × exposure × vulnerability.

To better elucidate the moderating role of vulnerability, we examined the relationship between cropland exposure and resulting risk across different vulnerability quartiles (Fig. 6). Across all hazards and scenarios, the slope between exposure and risk systematically steepens from the lowest (Q1) to the highest (Q4) vulnerability quartile. This effect is most pronounced under SSP3, characterized by regional rivalry and limited adaptation capacity, and weakest under SSP1, where sustainable development reduces vulnerability. Notably, even under moderate exposure conditions, highly vulnerable countries (Q4) exhibit risk levels comparable to or exceeding those of less vulnerable countries under extreme exposure, underscoring the amplifying influence of socioeconomic fragility. The consistent widening gap between quartiles across future scenarios suggests that disparities in adaptive capacity will increasingly drive the distribution of agricultural risk under climate change.

Focusing on the major breadbasket countries further illustrates how these dynamics translate into distinct trajectories of agricultural risk (Suppl. Fig. 11). The seven countries exhibit heterogeneous responses across hazards and scenarios. For floods, risk approximately doubles in the USA, Nigeria, and India across almost all scenarios, with a moderate increase in Argentina, particularly under SSP2. Heatwaves produce the strongest and most widespread amplification: the USA, Ukraine, Nigeria, and India show large increases across all scenarios, with fourfold increases under SSP3, whereas Brazil and Argentina exhibit pronounced intensification under SSP3 and more moderate increases in the remaining SSPs. China experiences a moderate increase in heatwave-related agricultural risk, most clearly under SSP3. For drought, risk increases consistently across all scenarios in the USA, while in the other breadbasket countries, the amplification is concentrated under SSP3. Compound hot–dry events broadly mirror the heatwave response, but with slightly larger risk amplification. Overall, these patterns indicate that the type of climate extreme strongly shapes the magnitude and distribution of future agricultural risk in breadbasket countries, underlining the importance of a multi-hazard perspective in agricultural risk assessment.

## Discussion

The spatial convergence of high exposure and high vulnerability identified in this study underscores the potential for simultaneous disruptions across major breadbasket regions. Such co-occurring or sequential failures could amplify volatility in global food markets, threaten trade-dependent nations, and strain humanitarian response systems. By linking physical climate risks with socio-institutional capacity, our analysis provides a framework to anticipate where global food system instability may first emerge under intensifying extremes. In doing so, this study delivers a global, multi-hazard assessment that explicitly integrates spatially explicit cropland exposure with a validated, data-driven vulnerability index, thereby revealing future exposure–vulnerability relationships that are absent in the historical period.

Our findings highlight that breadbasket areas, especially in South Asia, Sub-Saharan Africa, and the United States, are projected to experience the greatest increases in cropland exposure. Across all extremes and scenarios, ensemble medians show that exposure in these regions increases by 2–8 million km² for heatwaves, 0.5–3 million km² for droughts, 0.4–1.5 million km² for compound hot–dry events, and 0.4–1.0 million km² for floods by the end of the century relative to the historical baseline. Among different crops, cereal crops such as maize and rice are expected to be the most exposed to climate extremes, particularly under scenarios characterized by high population growth, regional rivalry, and limited socio-economic development (e.g., SSP3), or by fossil fuel-driven economic growth with weak climate policies (e.g., SSP5). These regions are not only physically vulnerable to intensifying extremes^51^ but also often lack the institutional and infrastructural capacity to buffer these impacts, as emphasized in recent vulnerability assessments^52^. In contrast, scenarios such as SSP1 and SSP2, which assume more inclusive development, stronger institutions, and more proactive sustainability efforts, are associated with lower cropland exposure, with increases approximately 17–85% smaller than in SSP3–7.0 and SSP5–8.5, despite comparable levels of climatic forcing^50^.

Previous global crop modeling studies for future climates have predominantly assessed the effects of mean global warming or CO_2_ fertilization^12–14,26,30^ rather than future climate extremes^20,23^. These studies consistently report average yield reductions of 6.0% for wheat, 3.2% for rice, 7.4% for maize, and 3.1% for soybean per degree of global warming^53,54^, and project a 24% decline in global maize production by the end of the century under SSP5-8.5^20^. For the historical period, Heinicke et al.^55^ evaluated 13 crop yield models and found mean relative yield declines under drought of 4.3% (maize), 1.3% (rice), 3.4% (soybean), and 6.7% (wheat), and under heatwaves of 8.2% (maize), 2.2% (soybean), and 4.5% (wheat). Empirical analyses of national cereal production likewise show 9–10% losses during droughts and extreme heat^56^. Yield losses are most pronounced when high temperatures coincide with dry conditions (compound hot–dry events), particularly during sensitive growth stages^47,57–59^, with temperature effects further modulated by precipitation^60^ and soil moisture^61^. For example, during the 2003 European compound heatwave and drought event, wheat and maize yields declined by 11% and 21%, respectively^62^. Zhao et al.^48^ also show that compound hot–dry–windy events are particularly damaging to U.S. wheat, causing an average 4% reduction per 10 h of exposure during heading to maturity. Building on this historical and modeling evidence, the projected increases in cropland exposure to climate extremes identified here are therefore expected to translate into substantial yield reductions, especially in major breadbasket regions. While all future impact assessments inevitably involve uncertainty, our analysis explicitly quantifies and communicates these uncertainties across multiple dimensions. Despite variability, ensemble medians consistently indicate substantial increases in cropland exposure across hazards and regions, demonstrating that the identified patterns are robust across models, crops, and scenarios.

A key finding of our study is the emergence of a strong relationship between vulnerability and cropland exposure in future scenarios, a pattern absent in the historical period. This relationship becomes particularly pronounced under SSP2, SSP3, and SSP4, with a statistically significant relationship (*p* < 0.001) indicating that countries with higher exposure also tend to exhibit higher vulnerability. These results reinforce the vulnerability-scenario framework^63^ and align with previous work highlighting the importance of structural factors in shaping climate risk^64,65^. Accordingly, future patterns of agricultural risk are governed not only by physical climate hazards but increasingly by socio-economic development and vulnerability, marking a shift away from the historically dominant role of the spatial exposure of croplands to extremes.

We also find a growing inequality in exposure across breadbasket regions. Areas with high vulnerability and limited adaptive capacity are likely to face disproportionately higher risks, even when they are not the most hazard-prone in purely physical terms. For instance, under SSP5-8.5, median cropland exposure to extreme events in high-vulnerability countries is 1.1–3.3 times greater than in low-vulnerability countries. This pattern reflects the concept of climate injustice^66^, where socio-economically disadvantaged regions bear the brunt of climate impacts. Without targeted adaptation, existing disparities in climate vulnerability are likely to deepen, particularly in agriculturally dependent economies^67^. The potential for socio-economic development to mitigate exposure underscores the importance of proactive, equity-focused adaptation strategies, especially in regions central to global food supply chains^68^.

The mechanisms underlying this relationship clarify how institutional and human capacities shape regional responses to climate shocks. Strong governance enables timely resource allocation for risk management, such as maintaining irrigation and drainage, operating early-warning systems, coordinating disaster response, and enforcing land use regulations that limit exposure. Higher education and extension access enhance adaptive decision-making by improving risk awareness, facilitating technology adoption, and fostering innovation in farm management. By contrast, weak institutions and low human capital often lead to fragmented coordination and delayed or inadequate responses to extremes. These institutional and human dimensions therefore, act as buffers or amplifiers of physical exposure, determining whether climate shocks translate into systemic agricultural losses.

While this study provides global, spatially explicit projections of cropland exposure to multiple climate extremes under diverse emissions and socio-economic scenarios, several promising directions remain for future research. An important extension could be to evaluate the impacts of these extremes on crop yields using process-based crop models. However, this integration poses substantial challenges at the global scale due to the computational demands of simulating multiple crop types under various coupled stress conditions, the biophysical interactions between wet/dry and hot/cold extremes with crop physiology, and the complexity of accurately representing causal pathways in yield formation models^47,55,69,70^. Similar to our exposure analysis at grid scale (Fig. 1), future research might also explore vulnerability at finer spatial scales to capture subnational disparities and localized adaptation potential. Because vulnerability is computed at the national scale, substantial within-country heterogeneity in socioeconomic and institutional conditions is not represented. Developing subnational vulnerability indices (e.g., at state/province/district levels) that incorporate region-specific determinants, such as irrigation and drainage access, market connectivity, and post-harvest storage capacity, agricultural extension services, and access to credit/insurance, could allow the index to reflect local adaptive capacity rather than national averages. This finer resolution could help reveal within-country risk hotspots in major producers (e.g., the Indo-Gangetic Plain in India, the U.S. Midwest/Mississippi Basin, and MATOPIBA in Brazil) and could better align risk assessment with jurisdictions that implement adaptation. Such refinement could support targeted, equity-focused planning by directing resources to places and populations that are both highly exposed and least able to cope. In addition, several vulnerability indicators, such as governance-related metrics (e.g., institutional capacity and policy implementation effectiveness), are difficult to quantify systematically and often rely on qualitative assessments, which may introduce further uncertainty into the vulnerability estimates.

Moreover, there are important limitations associated with the global climate (GCM) and hydrological (GHM) simulations used in this study. Both modelling systems rely on simplified representations of key physical processes because fully resolving atmospheric and hydrological dynamics at fine spatial scales is computationally infeasible at the global level. In GCMs, processes such as convection, cloud microphysics, boundary-layer turbulence, and land–atmosphere coupling are parameterized, which affects the simulation of extreme precipitation^71,72^. GHMs likewise parameterize infiltration, groundwater–surface water interactions, floodplain storage, river routing, reservoir operations, and water withdrawals, with substantial structural differences across models^73^. Many global hydrological models, including several used in ISIMIP, employ coarse global drainage direction maps and one-dimensional routing along a single downstream link per grid cell. These schemes do not explicitly represent backflow or tidal and surge-driven hydrodynamics and are therefore not suited to resolving flood processes in flat delta regions^73,74^. Moreover, statistical methods used to estimate extreme events may introduce bias: both extreme value analysis^39^ and short-baseline frequency estimation can lead to overestimation of future flood occurrence^75^. Combined with the coarse resolution of GCMs and GHMs, the structural heterogeneity among models, and methodological biases, these factors contribute to uncertainty in global hazard projections.

Nevertheless, our findings carry clear and actionable implications for climate adaptation and food security policy. They underscore the urgent need for targeted, equity-centered, and proactive adaptation strategies in the world’s most vulnerable breadbasket regions, particularly where exposure to climatic hazards is rising faster than adaptive capacity^76^. Strengthening institutional, infrastructural, and governance systems will be pivotal to reducing agricultural risks, even under high-emission futures. Governments, development agencies, and multilateral institutions should therefore prioritize context-specific and inclusive adaptation actions in the regional hotspots identified by emerging risk assessments. This includes investing in climate-resilient infrastructure (e.g., irrigation networks, on-farm storage, drainage, and floodplain management) to reduce direct exposure in high-risk regions, strengthening governance and institutional coordination between national climate services and local agencies to improve risk communication and adaptive response, enhancing education, extension, and digital advisory systems to translate climate information into adaptive action at the farm level, expanding access to finance and insurance to buffer farmers against income shocks and enable recovery, and prioritizing adaptation finance for regions where high exposure coincides with low adaptive capacity, ensuring equity in resilience-building. In South Asia, investments in irrigation efficiency, groundwater recharge, and drought- or heat-resilient crop varieties can buffer yield losses associated with recurrent heat and water stress. Expanding access to climate-smart irrigation and precision agriculture technologies will also enhance long-term water-use sustainability^77,78^. In Sub-Saharan Africa, where limited institutional capacity exacerbates exposure, priority should be given to crop diversification, small-scale water storage, and farmer-led insurance and credit systems. These approaches strengthen resilience by stabilizing production, improving local food access, and supporting smallholder autonomy under climate uncertainty^79^. In the United States, exposure is driven primarily by climate change rather than structural adaptive deficits^80,81^. Adaptation should therefore focus on regional trade adjustments, modernization of irrigation infrastructure, and flexible water-allocation policies aligned with state and federal adaptation plans.

Across all breadbasket regions, accelerating early-warning systems, agricultural extension services, and climate-informed decision-support tools will be essential to translate adaptive capacity into tangible risk reduction. Evidence shows that early-warning investments yield “triple dividends”^82^ by saving lives, reducing economic losses, and strengthening long-term resilience, particularly when linked to community-based and digital advisory platforms. Crucially, coordinating adaptation across multi-breadbasket systems via interoperable early-warning and market information platforms, contingency trade arrangements, and regional risk-pooling is essential to limit cascading food insecurity risks and dampen systemic shocks to global supply chains.

Finally, because investments in capacity building and institutional strength often exhibit a temporal lag before reducing exposure, early, sustained, and well-targeted implementation is crucial. Safeguarding global food security under climate change thus requires a dual strategy: reducing physical hazards through technological and ecological innovation, while advancing socio-institutional transformation that directs financial, informational, and infrastructural resources toward those who are most exposed and have the weakest adaptive capacity.

## Methods

### Hydroclimatic data

The outputs from 26 global climate models (GCMs) participating in the Coupled Model Intercomparison Project Phase 6 (CMIP6) were used to quantify drought, heatwave, and compound hot-dry events. The analysis included historical simulations spanning 1971–2014 and future projections for 2015–2100, considering four Shared Socioeconomic Pathways (SSP1-2.6, SSP2-4.5, SSP3-7.0, and SSP5-8.5). The variables analyzed included precipitation, total runoff, total soil moisture, near-surface air temperature, relative humidity, and surface pressure. A complete list of the CMIP6 models used is provided in Suppl. Table 1.

To aggregate results across climate models, each GCM is treated as an equally plausible ensemble member. All central estimates reported in the main text and figures correspond to the multi-model ensemble median, unless stated otherwise. Projection uncertainty is quantified as the inter-model spread, computed for each metric across the full ensemble. Specifically, we report uncertainty as the 95th–5th percentile range in Figs. 1 and 2, and as the standard deviation across models in Fig. 3.

For flood analysis, we used data from the Inter-Sectoral Impact Model Intercomparison Project (ISIMIP), which focuses on hydrological processes, higher resolution, and calibration with observed data^83^. We obtained daily discharge simulations from seven ISIMIP global hydrological models (GHMs): CLM4.5^84^, H08^85^, JULES-W1^86^, LPJmL^87^, MATSIRO^88^, ORCHIDEE^89^, and WaterGAP2^8^^90^^.^. Each GHM was driven by three state-of-the-art, observation-based historical climate datasets: GSWP3^91^, PGFv2^92^, and WFD/WFDEI^93^. The GHMs were forced by climate projections from four CMIP5 GCMs (GFDL-ESM2M, HadGEM2-ES, IPSL-CM5A-LR, and MIROC5), under three Representative Concentration Pathways (RCP2.6, RCP6.0, and RCP8.5). Before being used to force the GHMs, the raw GCM outputs were bias-corrected to the EWEMBI reference dataset (EartH2Observe, WFDEI, and ERA-Interim data Merged and Bias-corrected for ISIMIP^94^) using a trend-preserving method^95^. As with the CMIP6 ensemble, all GCM–GHM combinations were equally weighted, and hydrological model uncertainty is represented by the inter-model spread (Figs. 1–3). Further methodological details on model structure, including canopy, snow, soil, groundwater, lake, reservoir, wetland and river compartments, human water use sectors, and associated water storage and flow schemes, are provided in Telteu et al.^73^.

### Cropland data

To assess cropland exposure to climate extremes, we obtained total cropland proportions for historical (2005) and future (2085) periods from the AIM-SSP/RCP Gridded Emissions and Land-use dataset at a 0.5° × 0.5° resolution^96^ for five scenario combinations: SSP1–RCP2.6, SSP2–RCP4.5, SSP3–RCP6.0, SSP4–RCP4.5, and SSP5–RCP6.0. We further analyzed exposure for 10 dominant crop species and species groups, with future land-use patterns derived from the MAgPIE land-use model under SSP2^97,98^. To ensure consistency between historical (HYDE3.2) and future projections, the MAgPIE data were harmonized using a smoothing algorithm. Cropland projections were available for four GCMs (GFDL-ESM2M, HadGEM2-ES, IPSL-CM5A-LR, and MIROC5). For our analysis, we used the median of these projections for both historical and future periods (2005 and 2085).

### Vulnerability data

To estimate vulnerability, country-level data for six key indicators were utilized. Data on total population, age-specific population, education levels by age group, and age-specific gender distributions, available at a decadal resolution from 2010 to 2100, developed by the International Institute for Applied Systems Analysis (IIASA), were used. We also used the governance indicator (GI^99^) and the Human Development Index (HDI^100^) for the historical period and future SSPs (2005 and 2085).

We selected these indicators because future projections are available under SSPs 1–5 and they robustly capture socio-demographic and institutional dimensions of vulnerability, as detailed below. The GI serves as a proxy for institutional strength, reflecting governance quality and efficiency, which are critical for both immediate disaster response and long-term climate resilience^101^. This composite index includes GDP per capita, higher education attainment, and gender gaps in education, available for both historical periods and future SSP scenarios^99^. The HDI, derived from life expectancy, mean years of schooling, and Gross National Income per capita^100^, offers a broad measure of socioeconomic development and vulnerability.

Population size influences vulnerability and adaptive capacity by shaping resource availability and infrastructure demands: larger populations may strain resources, while smaller ones may lack sufficient labor or expertise. Tertiary education attainment (ages 25–64) represents human capital, enhancing public awareness and preparedness for hazards^102^. The age dependency ratio, defined as the ratio of non-working-age to working-age population, indicates economic burdens and differential vulnerability, as children and older adults are more exposed to climate risks due to mobility constraints, health vulnerabilities, and reliance on caregivers^103,104^. Gender ratio addresses differential impacts, as women, especially older women, often experience greater climate-related mortality due to physiological, social, and economic inequalities^105^.

The six indicators were subsequently integrated into a single vulnerability index, as detailed in the assessing vulnerability and risk subsection.

### Definition of farming regions

There is no universally accepted standard method for defining farming regions, and breadbasket regions have been defined in various ways in previous studies, typically based on present-day global production of specific crops^1,12,106,107^ and mostly at coarse national or sub-national scales^108^. Because our aim is to analyze cropland exposure at a finer spatial scale, we define farming regions at the 0.5° × 0.5° grid-cell level, consistent with the spatial resolution of the cropland dataset used in this study. To account for possible future changes in cropland extent, and thus in farming regions, we identify farming regions separately for the historical period and for each future SSP scenario, so that the classification reflects changing land use patterns (Suppl. Fig. 2).

We use the statistical distribution of cropland proportion to distinguish three types of farming regions. Grid cells with cropland proportion below the first quartile (Q1) are classified as limited farming regions and represent the least intensively cultivated 25% of cropland areas. Intermediate farming regions comprise grid cells with cropland proportion between the first and third quartiles (Q1–Q3), capturing moderately cultivated landscapes. Breadbasket regions are defined as grid cells with cropland proportion above the third quartile (Q3), corresponding to the most intensively cropped 25% of areas.

Historical breadbasket regions delineated using this approach correspond closely to known major crop-producing areas based on FAOSTAT^2^ and subnational production statistics, such as Shandong, Hebei, Henan, Jiangsu, and Anhui in China; Uttar Pradesh, Punjab, Maharashtra, and Karnataka in India; Paraná and São Paulo in Brazil; Córdoba in Argentina; and Wisconsin, Minnesota, and Illinois in the United States^1^.

### Quantification of cropland exposure to climate extremes

This study assesses cropland exposure to four types of climate extremes: floods, heatwaves, droughts, and compound hot-dry events. The probability of these events was calculated for both historical (the last 30 years of the 20th century) and future (the last 30 years of the 21st century) periods, under various RCP/SSP scenarios. For flood analysis, block maxima time series of daily discharge were derived for each grid cell for both periods. The generalized extreme value distribution (GEV) was applied to fit discharge block maxima and calculate flood return periods. Flood probability was estimated by determining the future return period of the historical 100-year flood event. Heatwaves were quantified by calculating the probability of the daily wet-bulb globe temperature exceeding the 90th percentile. Droughts were analyzed using the Multivariate Standardized Drought Index (MSDI^109^), which is based on the joint distribution of precipitation, runoff, and soil moisture using copula functions. Drought events were identified when the MSDI dropped below a threshold of −1. Compound hot-dry events were quantified by calculating the joint probability between the MSDI and the Standardized Temperature Index (STI^110^) using bivariate copulas. The Standardized Compound Hot-Dry Event Index (CHDI) was derived, and event occurrence probabilities were determined based on a threshold of −0.8^111^. A detailed description of the methodology for calculating climate extremes is provided in the supplementary material.

Following the calculation of event probabilities, cropland exposure was determined for each grid cell by multiplying the cropland proportion by the event probabilities, consistent with standard exposure definitions used in agricultural exposure studies^112,113^. For floods, the event probabilities are taken from ISIMIP2b simulations forced by RCP scenarios and are combined with the SSP-based cropland fractions described above to obtain flood exposure. For heatwaves, droughts, and compound hot–dry events, both event probabilities and cropland are derived from SSP scenarios. The percentage of cropland at each 0.5° x 0.5° grid cell was converted to square kilometers using spherical trigonometry to account for varying areas of GCM and GHM grid cells across latitudes. To assess changes in cropland exposure under different RCP/SSP scenarios, the difference in exposure between historical and future periods was calculated. In addition to total cropland, we also quantified changes in exposure for ten dominant crop species and species groups. Finally, exposure was aggregated for three farming-region types: limited farming, intermediate farming, and breadbasket regions.

The analysis of cropland exposure to different types of extremes was conducted for various combinations of climate and cropland changes to understand their contributions to differences in cropland exposure between farming regions. These combinations include static climate with dynamic cropland, which highlights differences in cropland exposure among farming regions driven by changes in cropland alone; dynamic climate with static cropland, reflecting differences in cropland exposure among farming regions resulting solely from climate change; and dynamic climate and dynamic cropland, examining how future climate and cropland changes together impact cropland exposure across farming regions.

### Assessing vulnerability and risk

Following IPCC AR5 risk framework^114^, we defined risk as a function of hazard, exposure, and vulnerability/coping-capacity. Vulnerability was quantified using a composite index derived from six socio-demographic and institutional indicators. Constructing a vulnerability index from a diverse set of indicators is methodologically challenging, particularly due to the need to assign appropriate weights to each indicator^115^, a process that is often subjective and inconsistent across studies^116–118^. To overcome this, we adopted an objective, data-driven approach that avoids arbitrary weighting. Specifically, we implemented Principal Components Factor Analysis (PCFA), a hybrid dimensionality reduction technique that integrates the variance-maximizing capabilities of Principal Component Analysis (PCA) with the error-accounting structure of Factor Analysis (FA). This method was selected to address the limitations of these individual objective, data-driven approaches: PCA’s inability to account for measurement error and FA’s dependence on strict multivariate normality, as well as its susceptibility to convergence issues when applied to small indicator sets. By operating on the complete covariance structure of all indicators, PCFA inherently accounts for their combined or synergistic effects, as it derives the common latent factor from shared variance rather than individual contributions. PCFA thus enables the extraction of interpretable latent dimensions of vulnerability from the statistical relationships among indicators, enhancing both the robustness and credibility of the resulting index.

We began by applying PCA to the standardized indicator matrix to extract the dominant latent dimension. The first principal component (PC1), which explained between 46% and 55% of the total variance across scenarios (Suppl. Fig. 12), was used to derive the initial factor loadings for the PCFA. These loadings formed the basis for estimating each indicator’s uniqueness (the proportion of variance not explained by the common factor), by evaluating the residual covariance after accounting for the initial factor structure. To validate the robustness of the 1-factor PCFA vulnerability index, we compared its results with those from a 2-factor composite vulnerability index. For this, we estimated a 2-factor PCFA model and combined the standardized factor scores from the first and second factors using weights proportional to the variance explained by each factor in the PCA step, so that the composite metric reflects both dominant and secondary vulnerability gradients. The resulting 2-factor composite index was then normalized to the same scale as the original 1-factor PCFA index. While the 2-factor solution explained a larger share of variance (65%–76% across scenarios) (Suppl. Fig. 12), the resulting 2-factor PCFA index yielded very similar vulnerability patterns to the 1-factor index, with high and statistically significant correlations for all scenarios (Suppl. Fig. 13). We therefore retain the 1-factor PCFA vulnerability index as the main metric used in the analysis.

To improve the numerical stability and fit of the factor solution, we applied an iterative refinement process using weighted least squares optimization. This procedure jointly updated the factor loadings and uniqueness estimates to enhance the model’s fit to the correlation matrix while preserving numerical stability. Iterations continued until the convergence criteria were met. Complete implementation details appear in Supplementary Text 3.

To evaluate the robustness and representativeness of the PCFA-based vulnerability index, we compared it against the Notre Dame Global Adaptation Initiative (ND-GAIN)^119^ vulnerability index, which is among the most comprehensive and widely used indicators of socioeconomic vulnerability. The ND-GAIN index is available at the country level for the historical period and quantifies vulnerability across six sectors (food, water, health, ecosystem services, human habitat, and infrastructure) based on 36 indicators. For consistency, we compared the PCFA index first with the ND-GAIN food-sector vulnerability, which is most directly related to agricultural and food system sensitivity, and subsequently with the overall ND-GAIN vulnerability encompassing all sectors. Validation was conducted through correlation and regression analyses to assess agreement, and (ii) Bland–Altman analysis to evaluate systematic bias and limits of agreement.

Risk was calculated as the product of hazard, exposure, and vulnerability. Changes in risk were then quantified as the ratio between risk projected under each SSP scenario, using future hazard, exposure, and vulnerability, and historical risk based on historical hazard, exposure, and vulnerability.

### Reporting summary

Further information on research design is available in the Nature Portfolio Reporting Summary linked to this article.

## Supplementary information

Supplementary Information

Reporting Summary

Transparent Peer Review file

## Data Availability

The CMIP6 global climate model outputs used to quantify droughts, heatwaves, and compound hot–dry events are available through the Earth System Grid Federation (ESGF) at https://metagrid.esgf-west.org/search. Daily river discharge simulations used for flood assessment were obtained from the ISIMIP portal (https://www.isimip.org/). The AIM-SSP/RCP gridded emissions and land-use dataset is available at https://www-iam.nies.go.jp/aim/data_tools/aimssp/aimssp.html, and the Landuse 15 Crops dataset can also be accessed via ESGF. Population, age structure, educational attainment, and gender distribution data were sourced from the International Institute for Applied Systems Analysis (IIASA) at https://iiasa.ac.at/models-tools-data. All processed data generated in this study are publicly available at Zenodo: 10.5281/zenodo.19844833.

## Code availability

The code developed in this study for calculating the PCFA-based vulnerability index is publicly available at 10.5281/zenodo.19844833.
