# Supplementary material for: Multi-hazard exposure and vulnerability drive rising agricultural risk in global breadbasket regions
Source: Nat Commun. 2026 Jun 18;17:7706. doi: 10.1038/s41467-026-74617-5 (PMC13434463; doi:10.1038/s41467-026-74617-5)
Supplement: Supplementary file 1 [file 41467_2026_74617_MOESM1_ESM.pdf]

Supplementary Information

for

Multi-hazard exposure and vulnerability drive rising agricultural risk in global breadbasket regions

Hossein Tabari[1,2,3] and Parisa Hosseinzadehtalaei[4,5]

[1]Faculty of Applied Engineering, University of Antwerp, Antwerp, Belgium.

[2]Department of Meteorological and Climate Research, Royal Meteorological Institute of Belgium, Uccle, Belgium.

[3]United Nations University Institute for Water, Environment and Health (UNU-INWEH), Hamilton, Richmond Hill, Canada.

[4]Department of Physics and Astronomy, Ghent University, Ghent, Belgium.

[5]Sino-Belgian Joint Laboratory for Geo-Information, Ghent, Belgium

Corresponding author:

Hossein Tabari (hossein.tabari@uantwerpen.be)

Contents of this file

Supplementary Texts 1-3

Supplementary Figures 1-13

Supplementary Table 1

Supplementary Text 1. Calculation of climate extremes

Flood

To quantify flood, we extracted the annual block maxima of daily river discharge at each grid cell for both the historical (1971–2000) and future (2070–2099) periods. These maxima were fitted using the Generalized Extreme Value (GEV) distribution, which is widely recognized as the most appropriate model for block maxima series. Using the fitted GEV parameters—location (μ), scale (σ), and shape (ξ)—we calculated discharge values associated with return periods (T) of 100 years.

To evaluate how the frequency of historical flood magnitudes may shift in the future, we estimated the future return period corresponding to the discharge thresholds historically associated with T = 100 years. This was done using the GEV cumulative distribution function, $\psi(I_T)$, which gives the probability of a discharge not exceeding a given value I_T. The return period is then calculated as (Tabari et al., 2021):

$$T = \frac{1}{1-\psi(I_T)} \tag{1}$$

where

$$\psi(I_T) = exp\left\{-\left[1 + \xi\left(\frac{I_T-\mu}{\sigma}\right)\right]^{-\frac{1}{\xi}}\right\} \tag{2}$$

for $1 + \xi\left(\frac{I_T-\mu}{\sigma}\right) > 0$

The inverse of this relationship allows us to compute the discharge associated with a given return period T:

$$I_T = \begin{cases} \mu + \frac{\sigma}{\xi}\left\{1 - \left[-\ln\left(1 - \frac{1}{T}\right)\right]^{\xi}\right\} & if \quad \xi \neq 0 \\ \mu - \sigma ln\left[-\ln\left(1 - \frac{1}{T}\right)\right] & if \quad \xi = 0 \end{cases} \tag{3}$$

Heatwave

The combined influence of air temperature and humidity plays a critical role in shaping environmental conditions that contribute to heat-related stress on human health (Chen et al., 2020). High humidity impairs the body's ability to cool itself through evaporation—the primary mechanism for heat dissipation. When elevated humidity coincides with high air temperatures, the risk to human health and survival increases significantly.

To assess heatwaves, we used the wet bulb globe temperature (T_{WBG}), which integrates both thermal and moisture effects. It is calculated as a weighted average of the wet-bulb temperature (T_{WB}) and near-surface air temperature (T):

$$T_{WBG} = 0.7T_{WB} + 0.3T \quad (4)$$

T_{WB} is derived from air temperature, relative humidity, and pressure based on the equivalent potential temperature formulation (Schwingshackl et al., 2021). Relative humidity, in turn, is estimated from specific humidity, surface pressure, and air temperature using an enhanced version of the Magnus formula (Alduchov and Eskridge, 1996). We defined the probability of heatwaves at each model grid cell as the proportion of days during which T_WBG exceeds the 90th percentile of the historical T_{WBG} distribution from 1971 to 2000.

Drought

Assessing the impacts of climate change on drought is more robust when using direct outputs from climate models—such as soil moisture and runoff—which explicitly capture the underlying physical processes often absent in simpler index-based approaches like SPI and SPEI (Milly and Dunne, 2016, Greve et al., 2019). These traditional indices frequently underestimate drought severity, especially in arid regions that are particularly vulnerable to climate change (Hosseinzadehtalaei et al., 2021).

To better account for the interdependencies among key hydrological variables, we applied the Multivariate Standardized Drought Index (MSDI; Hao and AghaKouchak, 2013), a method increasingly adopted for global drought assessments under climate change. MSDI integrates information from precipitation, runoff, and soil moisture by analyzing their joint distribution using copula-based techniques (Tabari and Willems, 2023; Adeyeri et al., 2023).

For each grid cell in the CMIP6 global climate models, we constructed the joint cumulative distribution function using a nested copula framework. Specifically, the trivariate copula $C(u)$ captures the combined probability of low precipitation, low runoff, and low soil moisture conditions:

$$C(u_1, u_2, u_3) = P(F_{X_1}(X_1) \leq u_1, F_{X_2}(X_2) \leq u_2, F_{X_3}(X_3) \leq u_3) \quad (5)$$

where F_{X_i} are the marginal cumulative distribution functions for each variable.

We used a two-level nested copula (Tabari and Willems, 2022), where one copula governs the dependency between runoff and soil moisture, and another links this pair to precipitation:

$$C(u) = C_0\big(u_{1,}C_1(u_2, u_3)\big) \quad (6)$$

The resulting joint probability was then transformed into a standard normal score to obtain the MSDI:

$$MSDI = \Phi^{-1}(p) \quad (7)$$

where Φ^{-1} is the inverse of the standard normal cumulative distribution function.

A month was classified as a drought event if its MSDI dropped below -1. We then calculated the drought probability for historical (1971–2000) and future (2071–2100) time windows as the percentage of months falling below this threshold.

Compound hot-dry event

Understanding compound hot-dry extremes requires a framework that captures the interplay between heat and drought, as well as the contribution of multiple water balance components. To this end, we quantified these events by estimating the joint probability of high temperatures and drought using bivariate copulas applied to the Standardized Temperature Index (STI; Zscheischler et al., 2014) and the MSDI.

Three distinct copula types were used to represent different dependence structures between hot and dry conditions (Ribeiro et al., 2020):

- Gaussian copula, which characterizes symmetric dependence across the full distribution, offering a general overview of co-occurrence patterns.
- Gumbel copula, which emphasizes upper tail dependence, ideal for capturing the concurrence of extreme heat and drought.
- Clayton copula, which highlights lower tail dependence, suitable for detecting joint mild anomalies.

Despite these differences in tail sensitivity, the variations in output across copula types were minimal. Therefore, for clarity and consistency, we present results based solely on the Gaussian copula.

To create a unified index, we transformed the joint probability derived from the copula into a standard normal score—mirroring the approach used for the MSDI—and defined this as the Hot-Dry Index (CHDI). This index offers a standardized measure of compound hot-dry event severity. We classified an event as compound when the CHDI dropped below -0.8 (Wu et al., 2020). The probability of occurrence for each period—historical (1971–2000) and future (2071–2100)—was calculated as the proportion of months that met these criteria relative to the total number of months.

Supplementary Text 2. Normalization of vulnerability indicators

Since the six proxy indicators of vulnerability have different units, they were normalized using linear min–max rescaling to a common scale ranging from 0.05 (indicating the lowest vulnerability) to 0.95 (indicating the highest) (Hosseinzadehtalaei et al., 2020). Indicators inversely related to vulnerability (i.e., HDI, GI, and education) were transformed so that higher normalized values consistently represent higher vulnerability. The normalization was applied as follows:

$$Z_c = \begin{cases} 0.05 + (0.9 \times \frac{X_c - X_{min}}{X_{max} - X_{min}}) & for\ direct\ relationship \\ 1 - [0.05 + (0.9 \times \frac{X_c - X_{min}}{X_{max} - X_{min}})] & for\ indirect\ relationship \end{cases} \tag{8}$$

where Z_c and X_c represent the normalized and original values of the indicator for country *c* respectively, and X_{min} and X_{max} are the minimum and maximum values of that indicator across all countries.

Supplementary Text 3. Estimation of the vulnerability index using the Principal Component Factor Analysis (PCFA)

1. Theoretical framework

We employed Principal Component Factor Analysis (PCFA), which integrates the optimization principle of Principal Component Analysis (PCA) with the measurement model of Factor Analysis (FA). Specifically, PCFA maximizes the variance of a linear combination of observed variables, subject to a unit-norm constraint on the weights:

$$\underset{\omega}{\text{argmax}}\ \text{Var}(X\omega) \quad \text{subject to} \quad \omega^T\omega = 1 \tag{9}$$

This is combined with the FA model:

$$X = Lf + \varepsilon, \quad \text{with} \quad Cov(\varepsilon) = \Psi \tag{10}$$

where X is the standardized data matrix, L is the factor loading matrix, f is the matrix of latent factor scores, ε is the vector of unique errors, and Ψ is a diagonal matrix capturing the unique variances of the observed variables.

2. Computational steps

Step 1: PCA initialization

We begin by performing Singular Value Decomposition (SVD) on the standardized matrix X:

$$X = U \sum V^T \tag{11}$$

The initial estimate of the loading matrix is derived from the first principal component:

$$L^0 = v_1 \sqrt{\lambda_1} \tag{12}$$

where v_1 is the first right singular vector and λ_1 is the largest singular value (i.e., variance explained by PC1).

Step 2: Uniqueness matrix estimation

An initial estimate of the uniqueness matrix Ψ is calculated as:

$$\Psi^0 = \mathrm{diag}(R - L^0 L^{0T}) \tag{13}$$

where R is the sample correlation matrix of X.

Step 3: Iterative optimization

We iteratively update the loading matrix using the following update rule:

$$L^{(k+1)} = R(\Psi^{(k)})^{-1} L^{(k)} (I + L^{(k)T} (\Psi^{(k)})^{-1} L^{(k)})^{-1} \tag{14}$$

Iterations continue until convergence is achieved, defined by the Frobenius norm of the difference between successive loading matrices:

$$\left\| L^{(k+1)} - L^{(k)} \right\|_F < 10^{-6} \tag{15}$$

Step 4: Scoring

Once the final loading matrix L is obtained, factor scores are computed using:

$$F = XS^{-1}L \tag{16}$$

where S is the covariance matrix of the observed variables (identity matrix in case of standardization). The resulting matrix F provides the latent factor scores representing vulnerability.

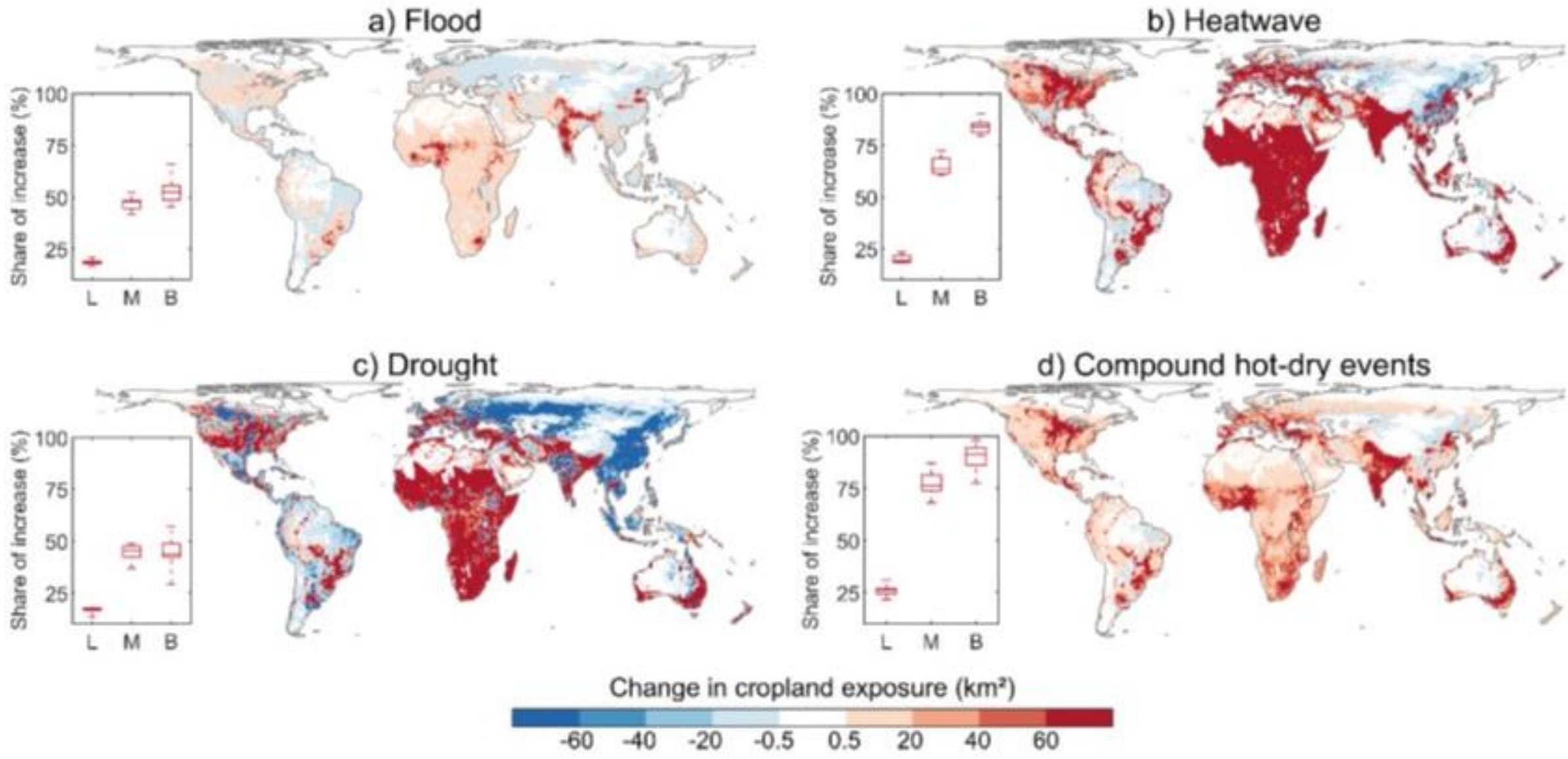

Supplementary Fig. 1 | Projected changes in global cropland exposure to climate extremes by the end of the 21st century under SSP1-2.6. The maps show the absolute change (km^2) in global cropland area exposed to (a) floods, (b) heatwaves, (c) droughts, and (d) compound hot-dry events between the late twentieth century and the late twenty-first century, based on the multi-model ensemble median. Cropland exposure is defined as the product of a climate-driven hazard and cropland proportion. For floods, the hazard is taken from RCP-forced ISIMIP2b simulations and combined with SSP-based cropland, whereas for the other extremes both hazard and land use are SSP-based. Insets in each panel summarize the spatial coverage of increasing exposure ("Share of increase (%)") across three farming regions: limited (L), intermediate (M) and breadbasket (B). Boxplots represent distributions across independent model ensemble members, with the center line indicating the median, boxes the interquartile range (25th–75th percentiles), and whiskers extending to the most extreme values within 1.5×IQR. Sample sizes are n = 28 (floods), 18 (heatwaves), 22 (droughts), and 22 (compound events).

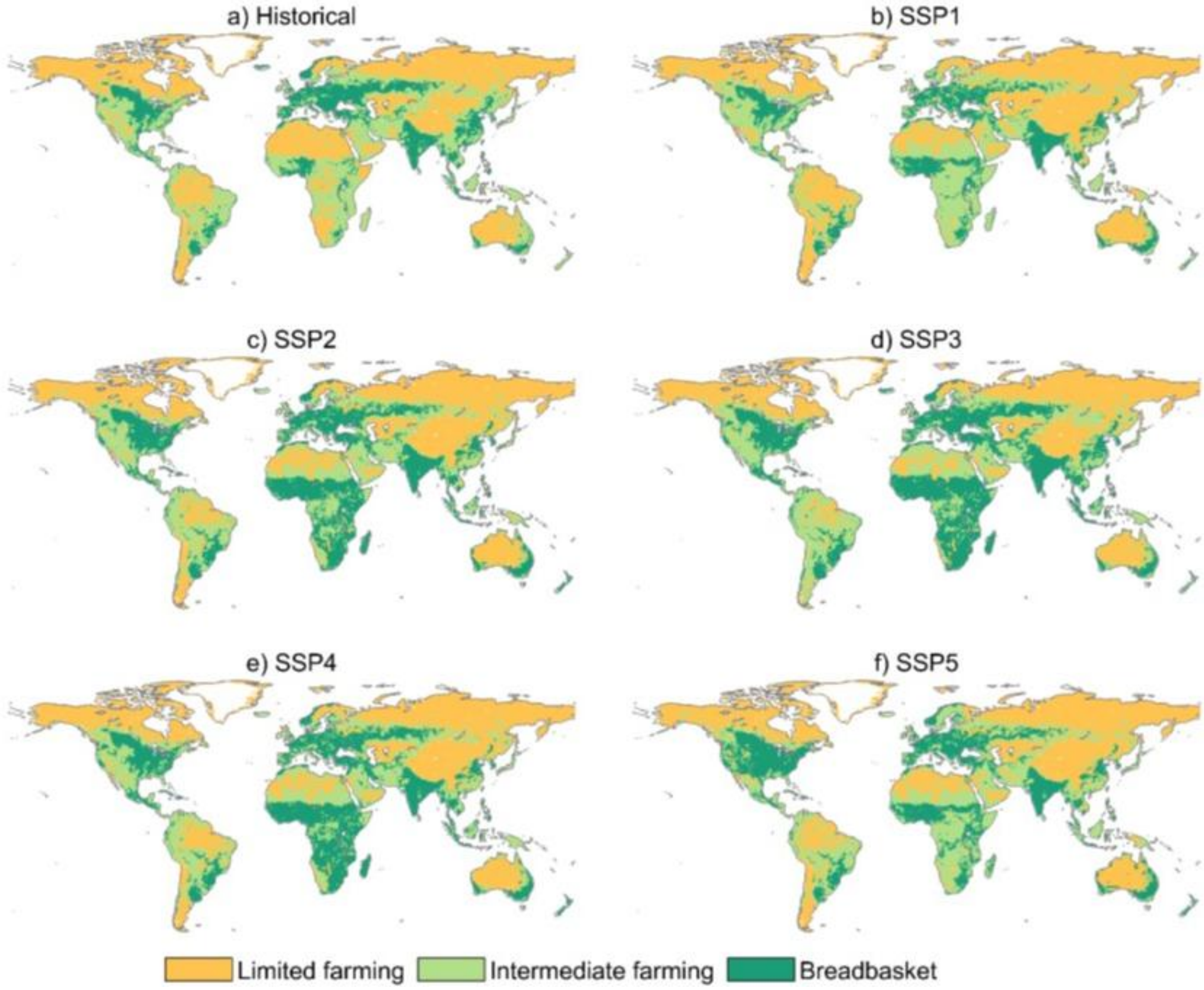

Supplementary Fig. 2 | Farming regions under historical and projected cropland scenarios. Maps show farming regions for (a) historical conditions and future projections under (b) SSP1, (c) SSP2, (d) SSP3, (e) SSP4, and (f) SSP5. Farming regions are classified according to cropland fraction into limited farming regions (grid cells below Q1), intermediate farming regions (grid cells between Q1 and Q3), and breadbasket regions (grid cells above Q3).

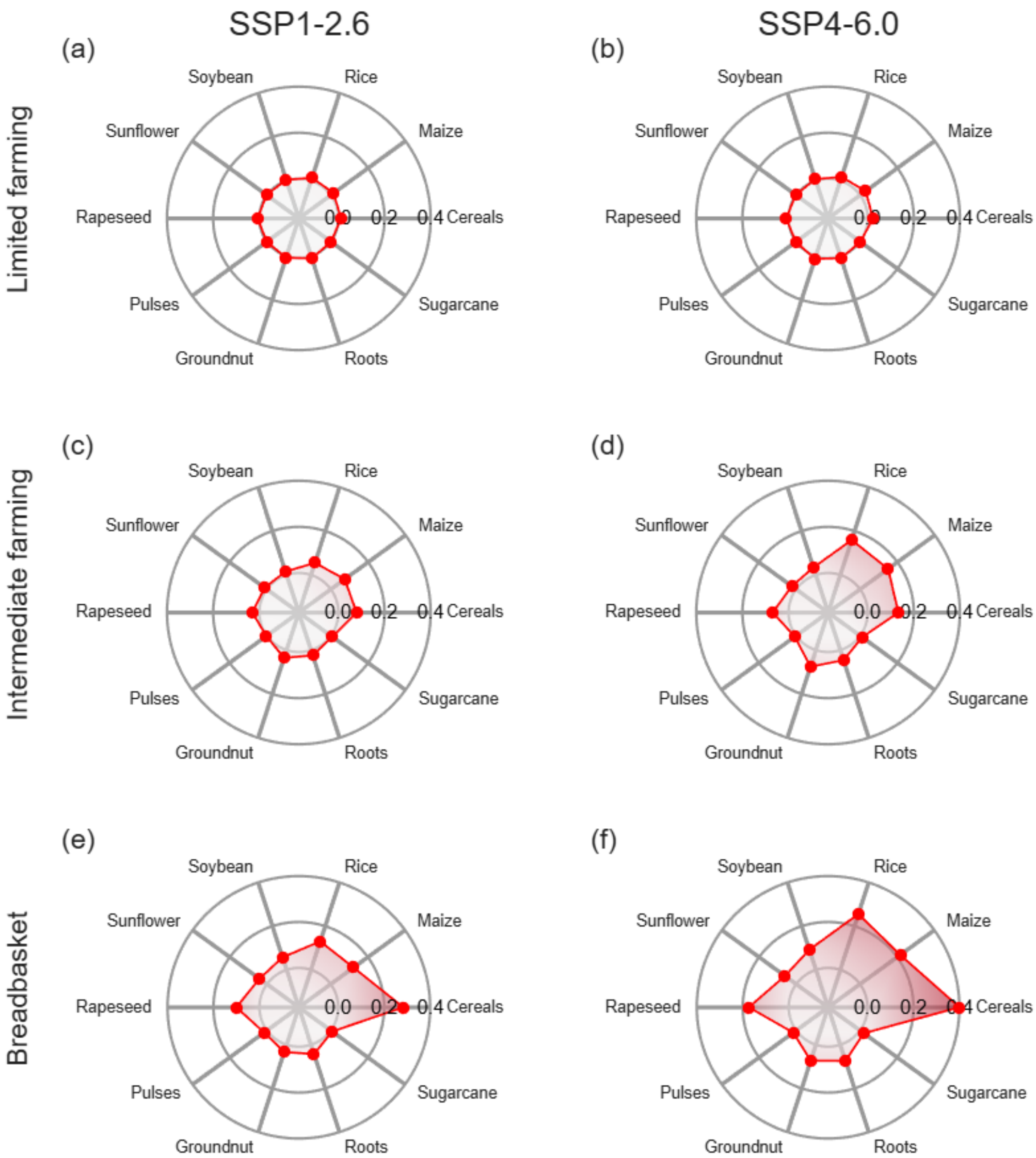

Supplementary Fig. 3 | Comparison of projected changes in global cropland exposure of different crops to flood across farming regions for SSP1-2.6 and SSP4-6.0. Shown are the differences in cropland exposure (×10^5 km^2) between the late 20th century and the late 21st century under SSP1-2.6 (a,c,e) and SSP4-6.0 (b,d,f) across limited farming (a,b), intermediate farming (c,d), and breadbasket regions (e,f). Values along the axes represent exposure changes for individual crops, and concentric circles indicate increasing levels of exposure change.

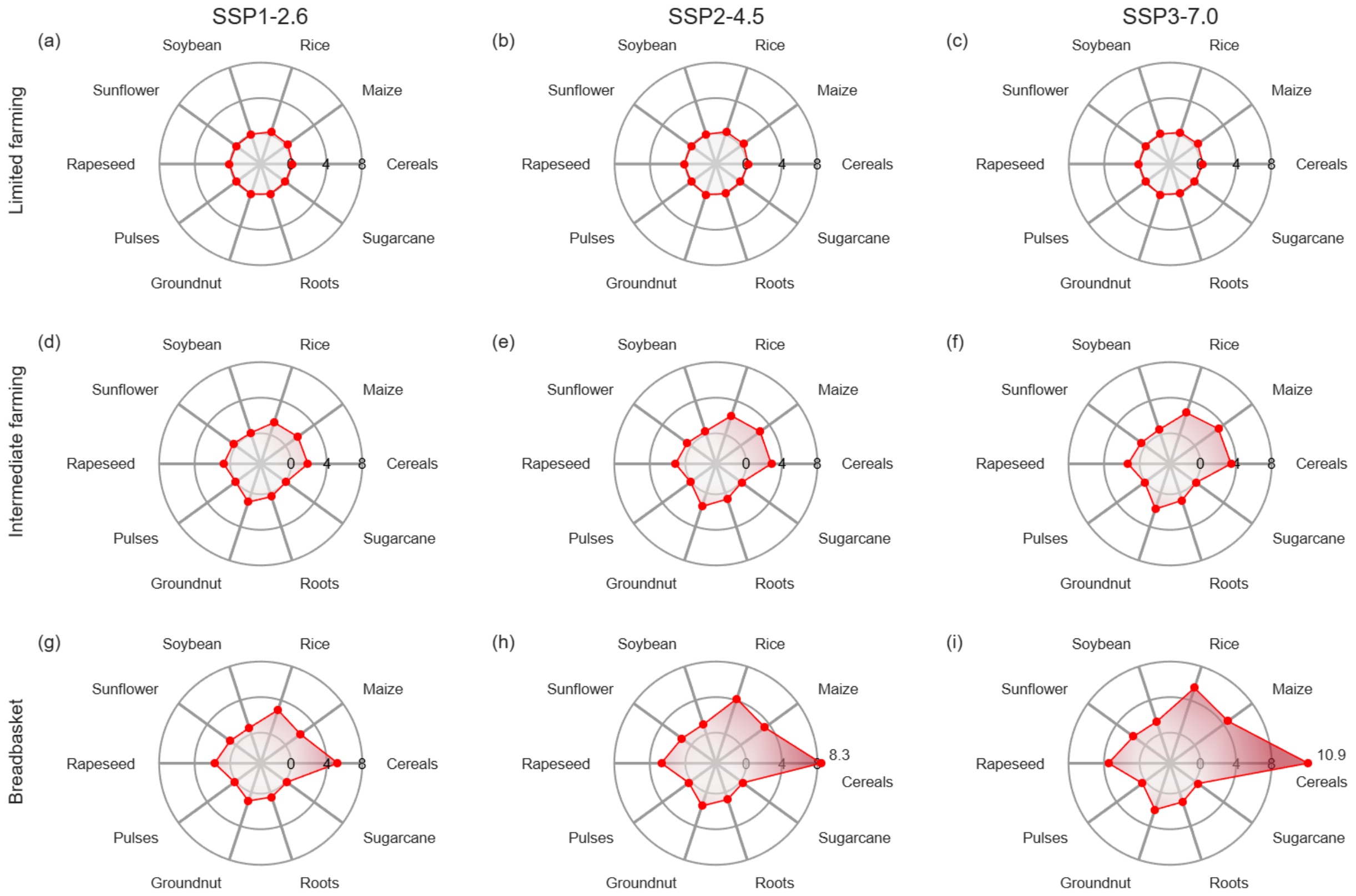

Supplementary Fig. 4 | Comparison of projected changes in global cropland exposure of different crops to heatwaves across farming regions for SSP1-2.6, SSP2-4.5, and SSP3-7.0. Shown are the differences in cropland exposure (×10^5 km^2) between the late 20th century and the late 21st century under SSP1-2.6 (a,d,g), SSP2-4.5 (b,e,h), and SSP3-7.0 (c,f,i) across limited farming (a–c), intermediate farming (d–f), and breadbasket regions (g–i). Values along the axes represent exposure changes for individual crops, and concentric circles indicate increasing levels of exposure change.

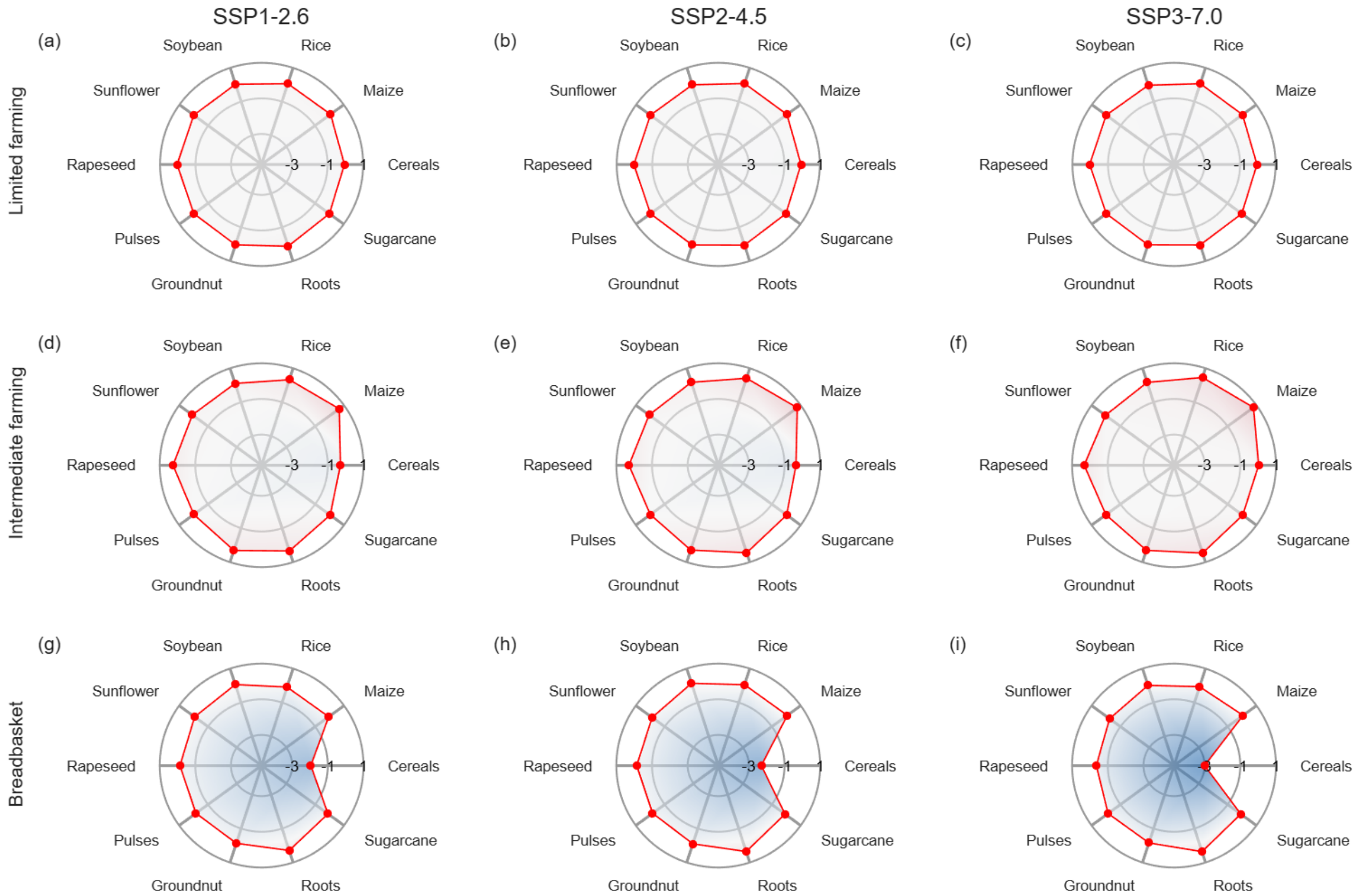

Supplementary Fig. 5 | Comparison of projected changes in global cropland exposure of different crops to drought across farming regions for SSP1-2.6, SSP2-4.5, and SSP3-7.0. Shown are the differences in cropland exposure (×10^5 km^2) between the late 20th century and the late 21st century under SSP1-2.6 (a,d,g), SSP2-4.5 (b,e,h), and SSP3-7.0 (c,f,i) across limited farming (a–c), intermediate farming (d–f), and breadbasket regions (g–i). Values along the axes represent exposure changes for individual crops, and concentric circles indicate increasing levels of exposure change.

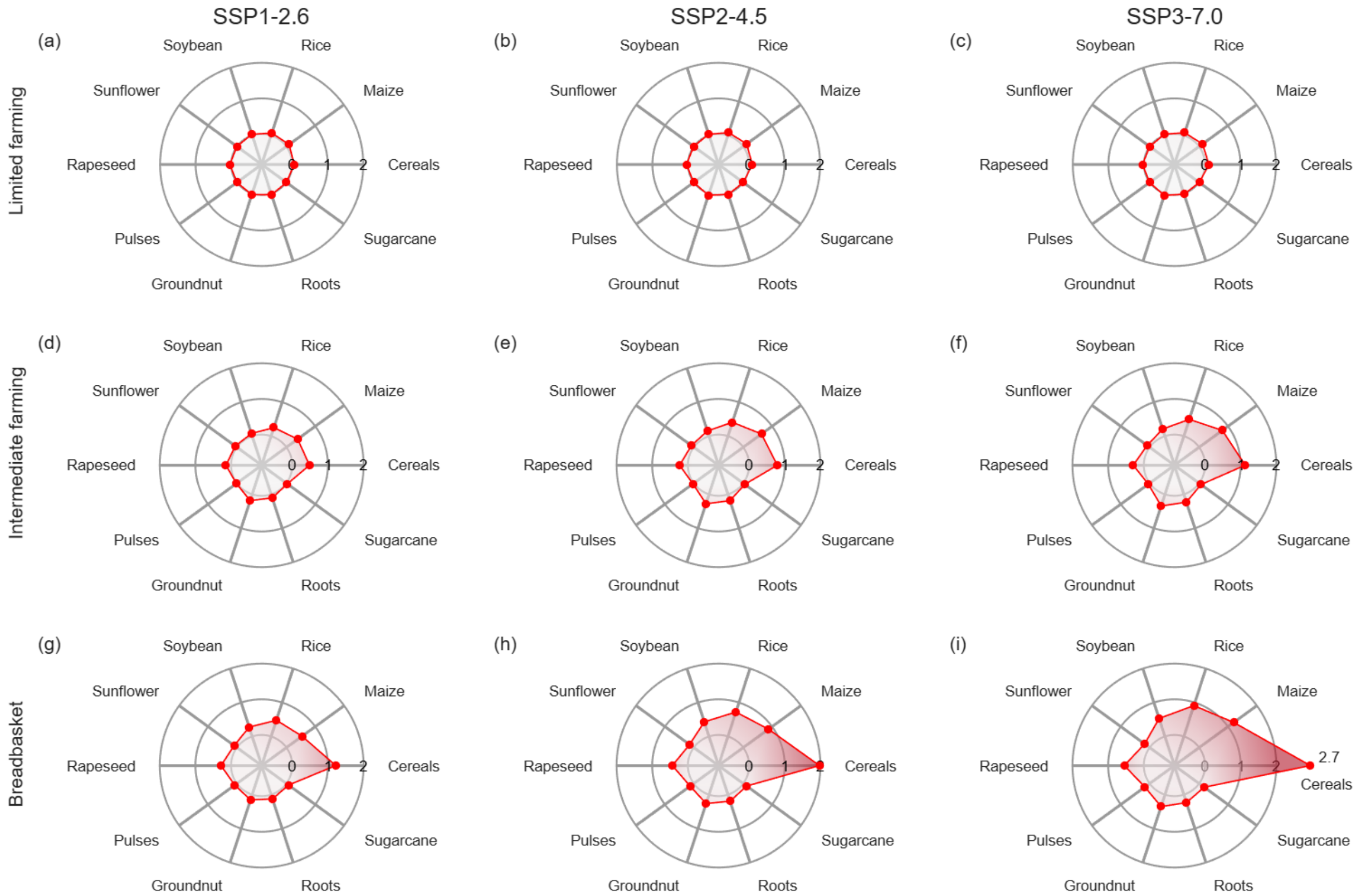

Supplementary Fig. 6 | Comparison of projected changes in global cropland exposure of different crops to compound hot-dry events across farming regions for SSP1-2.6, SSP2-4.5, and SSP3-7.0. Shown are the differences in cropland exposure (×10^5 km^2) between the late 20th century and the late 21st century under SSP1-2.6 (a,d,g), SSP2-4.5 (b,e,h), and SSP3-7.0 (c,f,i) across limited farming (a–c), intermediate farming (d–f), and breadbasket regions (g–i). Values along the axes represent exposure changes for individual crops, and concentric circles indicate increasing levels of exposure change

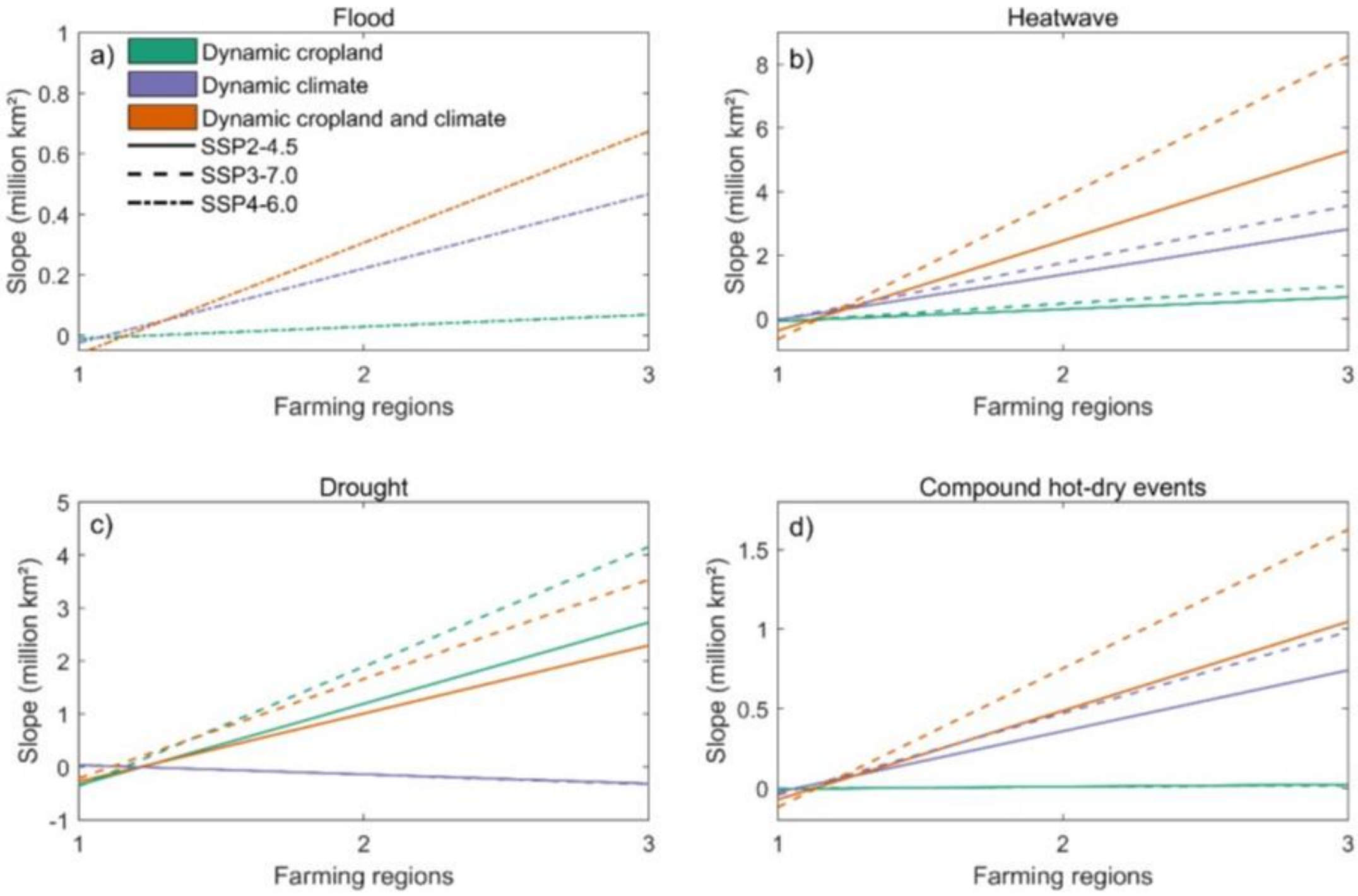

Supplementary Fig. 7 | Same as Fig. 5, but for scenarios SSP2-4.5, SSP3-7.0, and SSP4-6.0. Panel (a) presents flood results under SSP4-6.0, while panels (b), (c), and (d) show heatwave, drought, and compound hot-dry event results under SSP2-4.5 and SSP3-7.0.

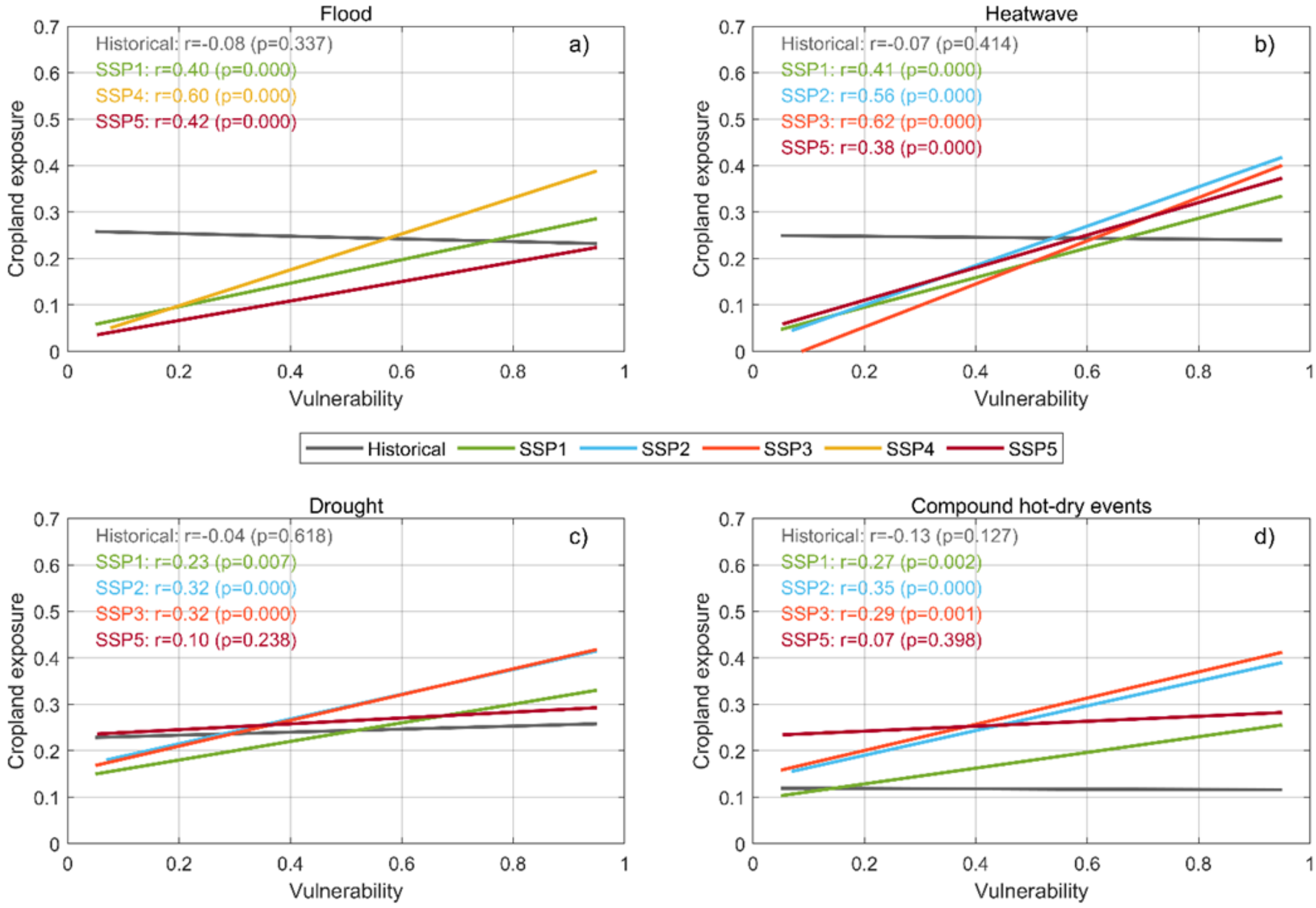

Supplementary Fig. 8 | Relationships between crop exposure to climate extremes and country vulnerability. Relationships between crop exposure and vulnerability for 151 countries under (a) flood, (b) heatwave, (c) drought, and (d) compound hot-dry events. Spearman correlation coefficients (r), together with their statistical significance (p values), are shown in each panel, with colors corresponding to the historical period and SSP scenarios.

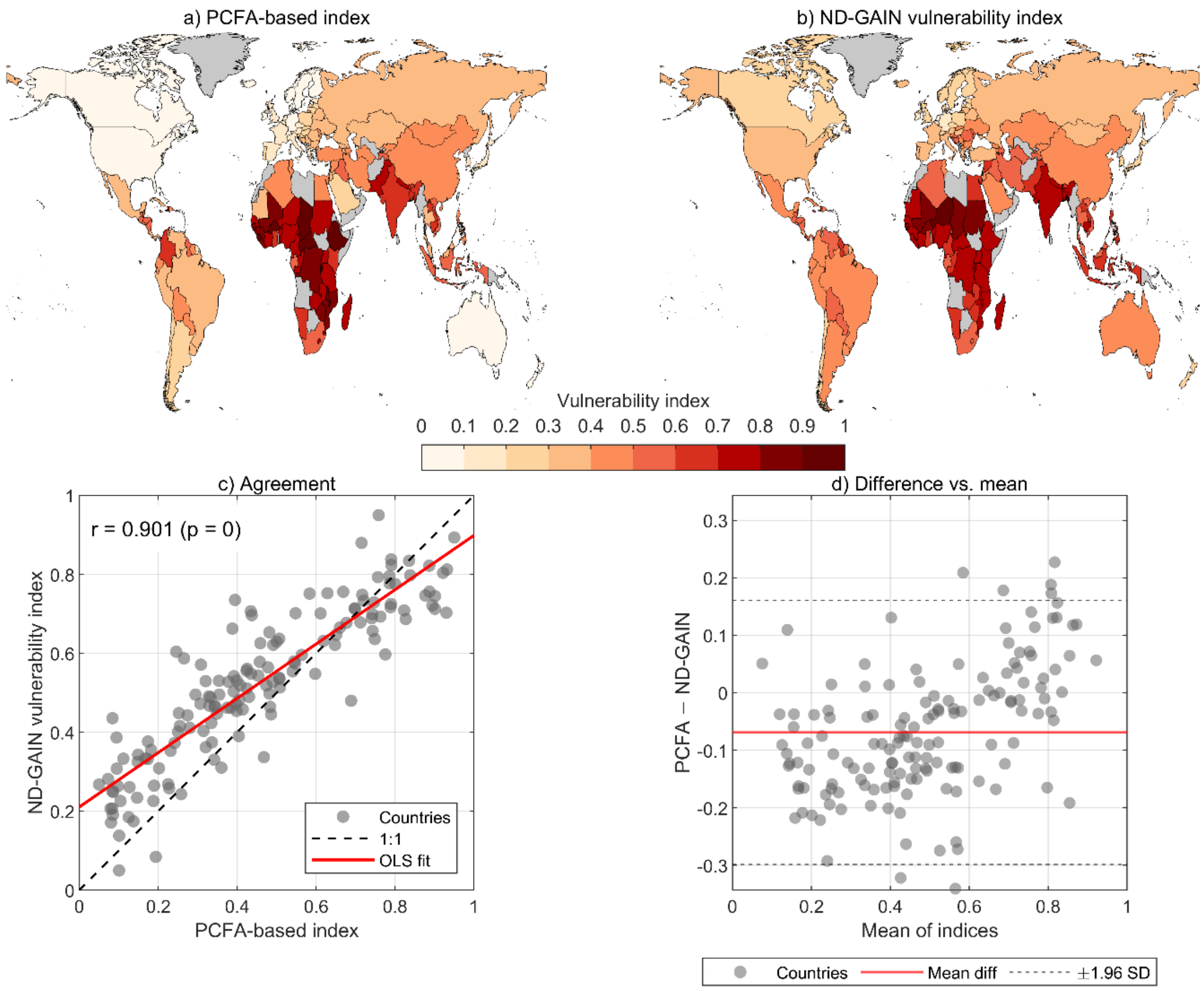

Supplementary Fig. 9 | Validation of the PCFA-based vulnerability index for the food sector. (a,b) Global maps of the PCFA-based vulnerability index (this study) and the ND-GAIN food-sector vulnerability for the historical period. (c) Country-level comparison with the 1:1 line (dashed) and ordinary least squares (OLS) fit (red). (d) Bland–Altman plot showing differences (PCFA − ND-GAIN) centered near zero and contained within ±1.96 SD.

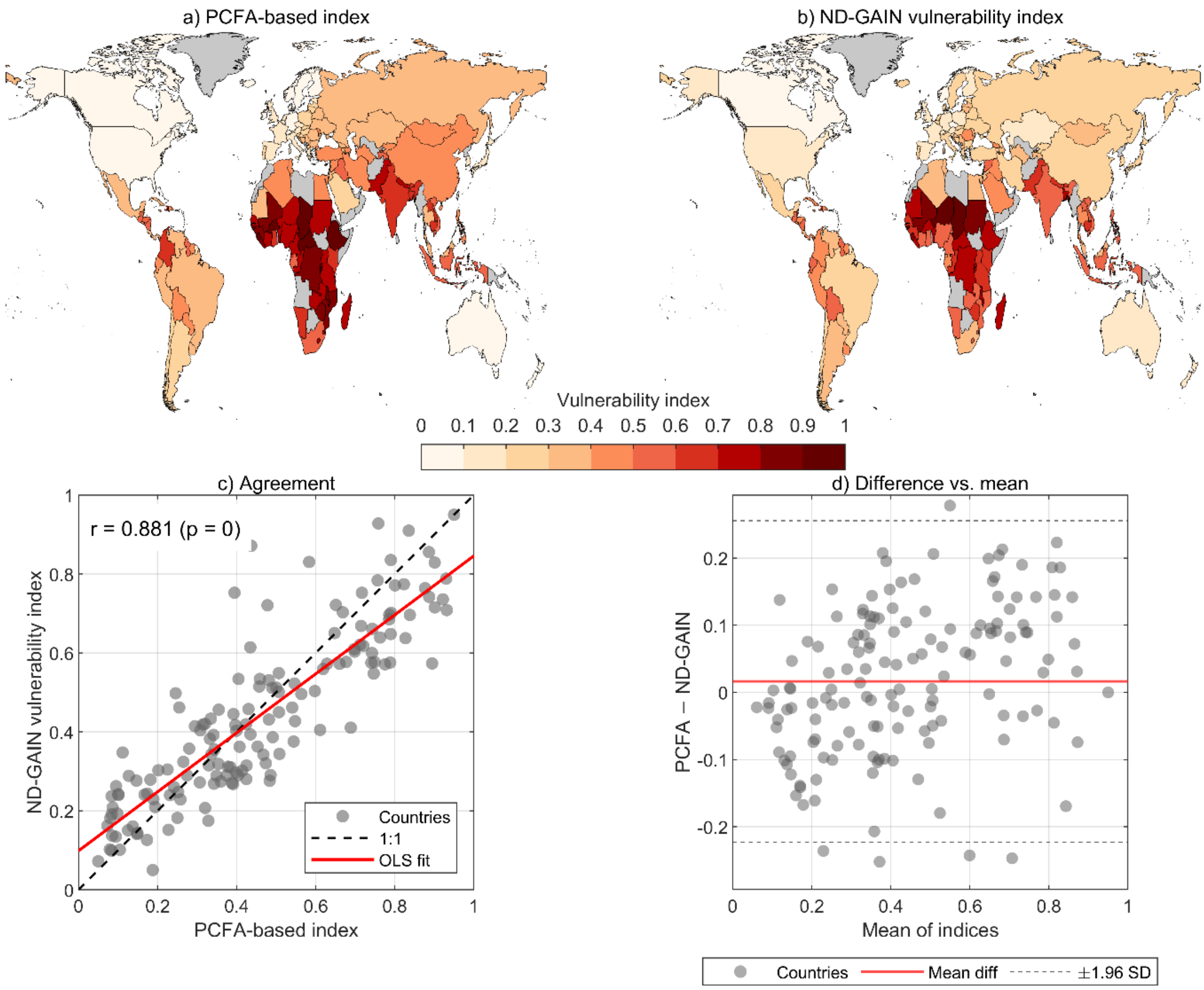

Supplementary Fig. 10 | Validation of the PCFA-based vulnerability index against overall ND-GAIN vulnerability (all sectors). (a,b) Global maps of the PCFA-based vulnerability index and the overall ND-GAIN vulnerability (all sectors) for the historical period. (c) Country-level comparison with the 1:1 line (dashed) and OLS fit (red). (d) Bland–Altman plot showing differences centered near zero and contained within ±1.96 SD.

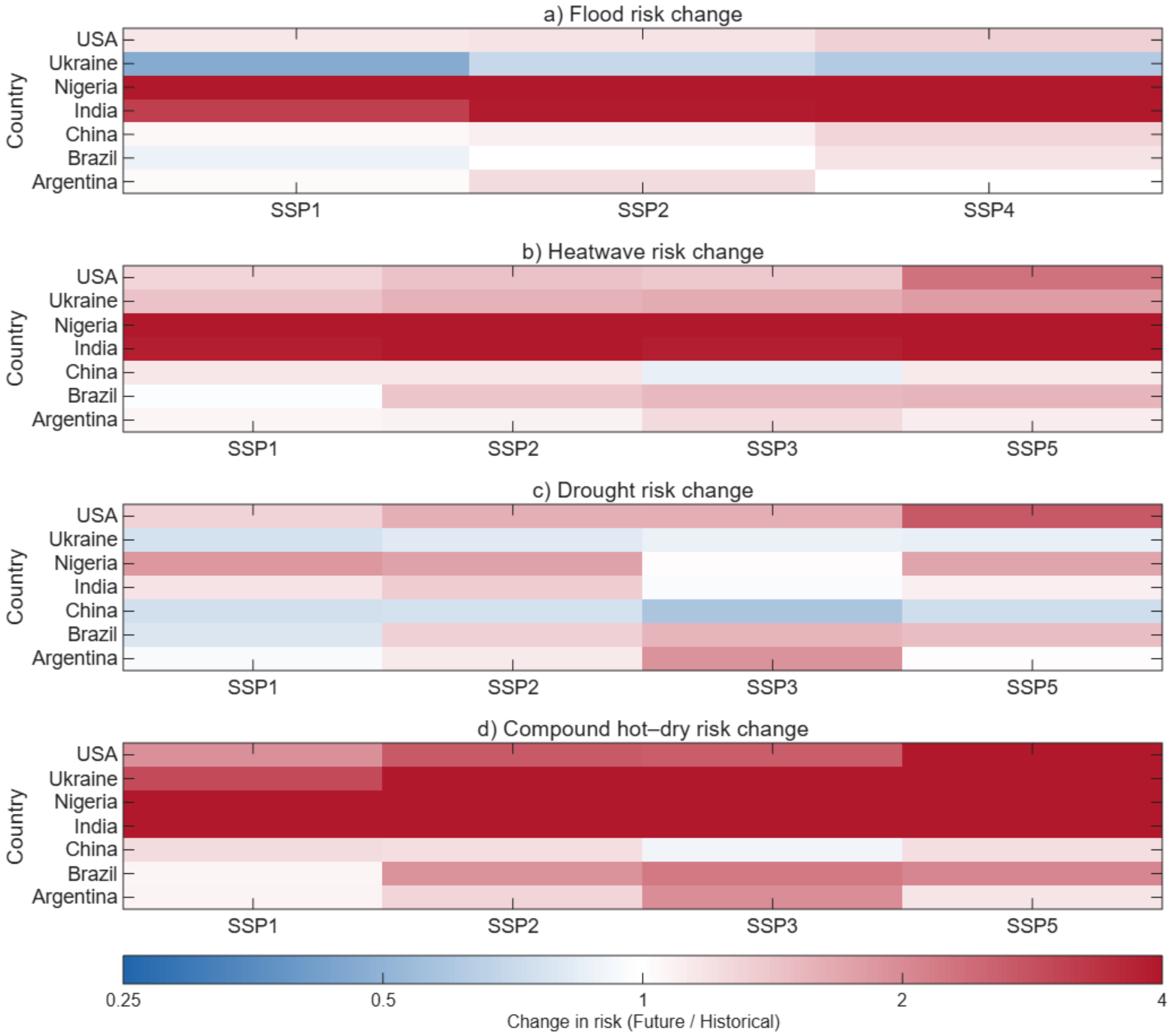

Supplementary Fig. 11 | Changes in agricultural risk across global breadbasket regions under different future scenarios. Heatmaps show the change in agricultural risk (Future / Historical) for a) floods, b) heatwaves, c) droughts and d) compound hot–dry events across seven major breadbasket countries (Argentina, Brazil, China, India, Nigeria, Ukraine and the USA). Risk is calculated following the IPCC risk assessment framework, as the product of hazard, exposure and vulnerability.

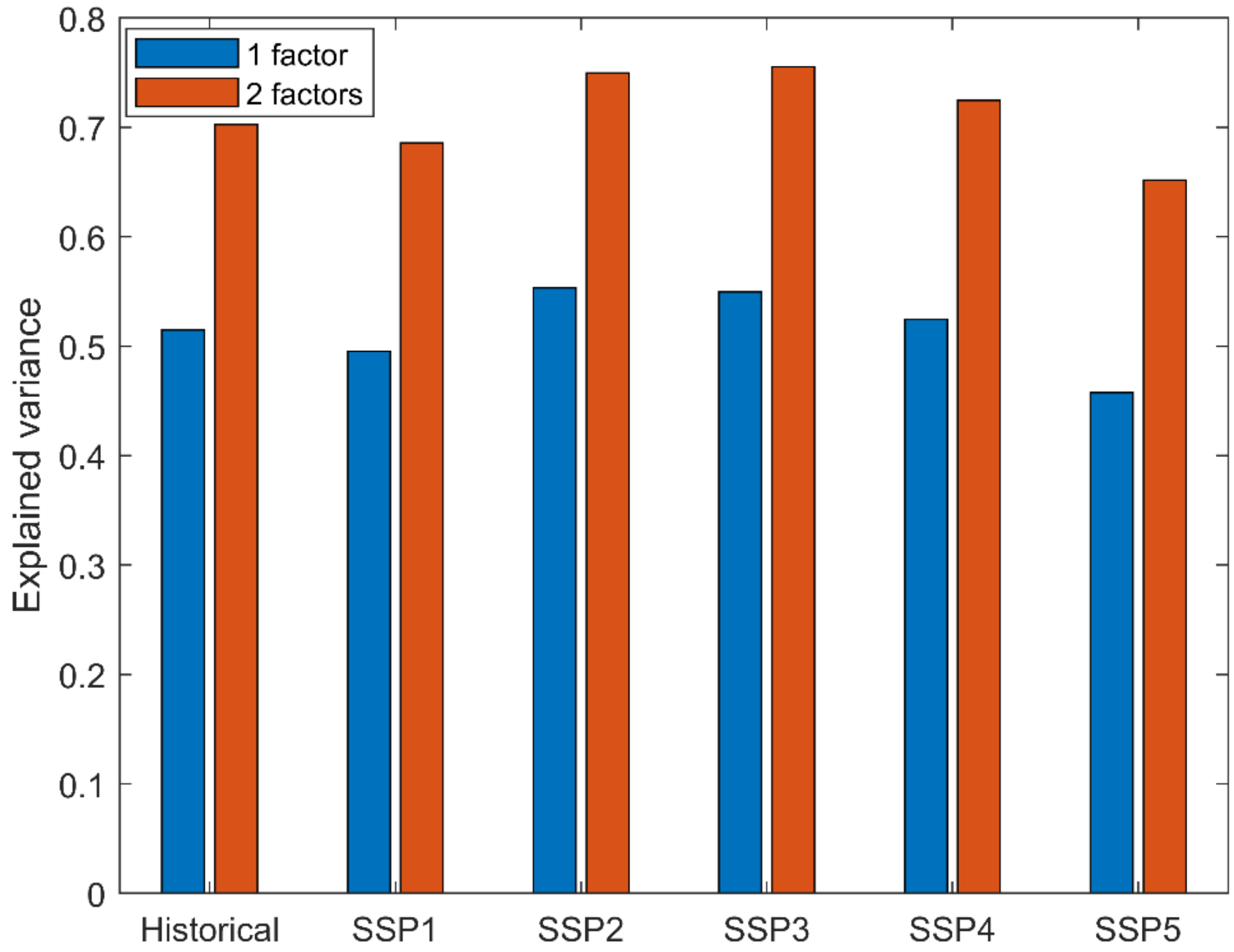

Supplementary Fig. 12 | Variance explained by 1-factor and 2-factor PCFA models. Explained variance for the 1-factor and 2-factor principal component and factor analysis (PCFA) models across the Historical and SSP scenarios. The 1-factor model captures the dominant latent structure of the socioeconomic indicators, while the 2-factor model substantially increases the proportion of variance explained, reflecting additional heterogeneity in the indicator set. The results confirm that although two factors describe more variance, a single factor captures the primary vulnerability gradient that drives cross-country differences.

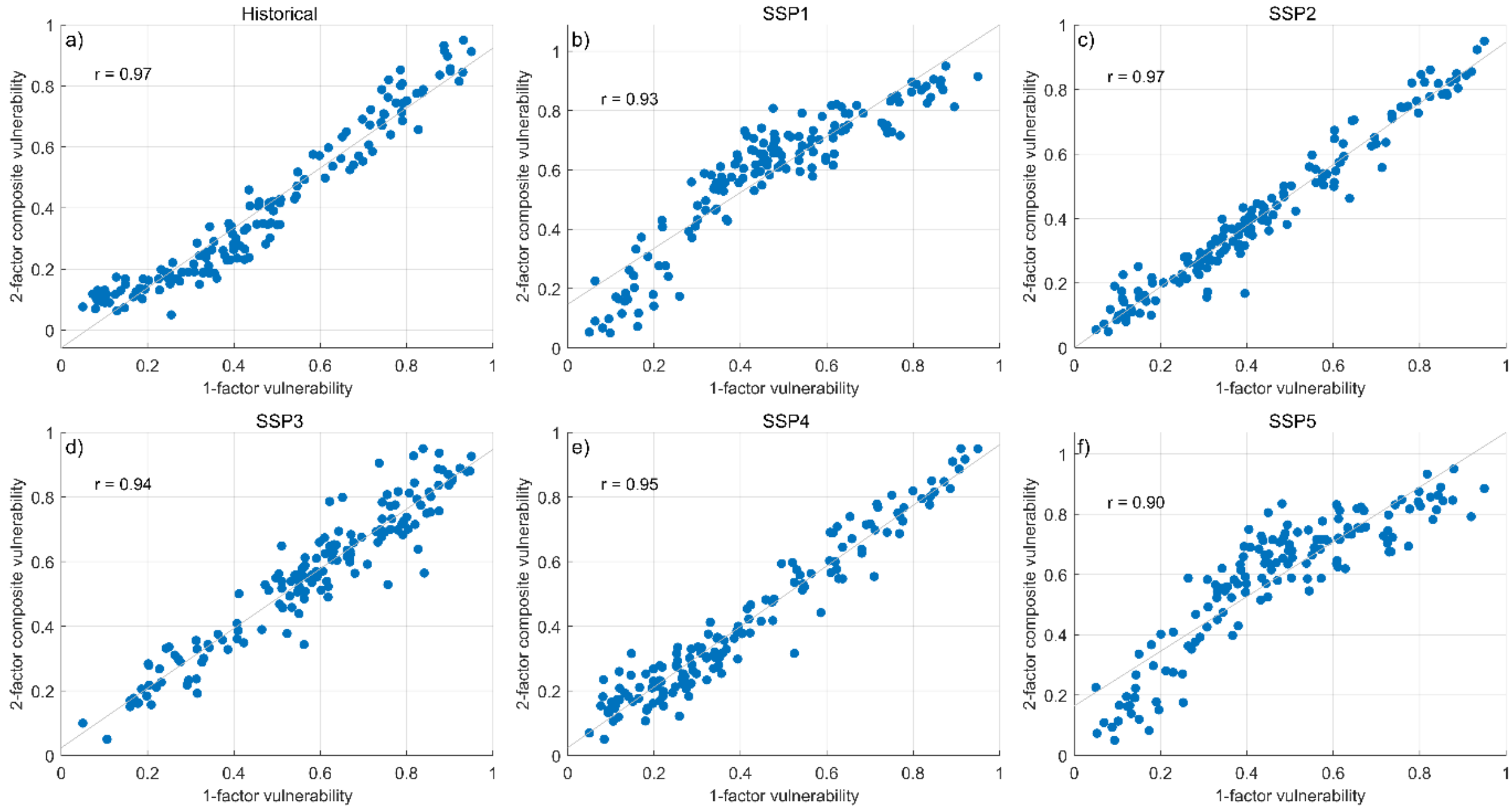

Supplementary Fig. 13 | Comparison between 1-factor and 2-factor PCFA vulnerability indices across scenarios. Panels a–f show the relationship between the 1-factor socioeconomic vulnerability index and the 2-factor composite vulnerability index, both derived using principal component and factor analysis (PCFA), for the historical, SSP1, SSP2, SSP3, SSP4, and SSP5 scenarios, respectively. Each point represents a country. Spearman rank correlation coefficients between the two indices are shown in each panel, and all correlations are statistically significant at the 0.01 level.

Supplementary Table 1. CMIP6 GCMs used in this study.

No.	Model name	Compound event	Drought	Heatwave
1	ACCESS-CM2	√	√	√
2	ACCESS-ESM1-5	√	√	√
3	BCC-CSM2-MR	√	√	
4	CAMS-CSM1-0	√	√	
5	CanESM5	√	√	√
6	CAS-ESM2-0	√	√	
7	CNRM-CM6-1	√	√	√
8	CNRM-ESM2-1	√	√	√
9	EC-Earth3			√
10	FGOALS-f3-L	√	√	
11	FGOALS-g3	√	√	√
12	GFDL-ESM4	√	√	√
13	INM-CM4-8	√	√	√
14	INM-CM5-0	√	√	√
15	KACE-1-0-G			√
16	IPSL-CM6A-LR	√	√	
17	MCM-UA-1-0	√	√	
18	MIROC6	√	√	
19	MIROC-ES2L			√
20	MPI-ESM1-2-HR	√	√	√
21	MPI-ESM1-2-LR	√	√	√
22	MRI-ESM2-0	√	√	√
23	NorESM2-LM	√	√	√
24	NorESM2-MM	√	√	√
25	TaiESM1	√	√	
26	UKESM1-0-LL			√

References

1. Adeyeri, O. E., Zhou, W., Laux, P., Ndehedehe, C. E., Wang, X., Usman, M., & Akinsanola, A. A. Multivariate drought monitoring, propagation, and projection using bias-corrected general circulation models. *Earth's Future* **11**(4), e2022EF003303(2023).
2. Alduchov, O. A. & Eskridge, R. E. Improved Magnus form approximation of saturation vapor pressure. *J. Appl. Meteorol.* **35**, 601-609 (1996).
3. Chen, X. et al. Changes in global and regional characteristics of heat stress waves in the 21st century. *Earth's Future* **8**, e2020EF001636 (2020).
4. Greve, P., Roderick, M. L., Ukkola, A. M., & Wada, Y. The aridity index under global warming. *Environ. Res. Lett.* **14**(12), 124006 (2019).
5. Hao, Z., & AghaKouchak, A. Multivariate standardized drought index: a parametric multi-index model. *Adv. Water Resour.* **57**, 12-18 (2013).
6. Hosseinzadehtalaei, P., Ishadi, N. K., Tabari, H. & Willems, P. Climate change impact assessment on pluvial flooding using a distribution-based bias correction of regional climate model simulations. *J. Hydrol.* **598**, 126239 (2021).
7. Hosseinzadehtalaei, P., Tabari, H., & Willems, P. (2020). Satellite-based data driven quantification of pluvial floods over Europe under future climatic and socioeconomic changes. Science of the Total Environment, 721, 137688.
8. Milly, P. C., & Dunne, K. A. Potential evapotranspiration and continental drying. *Nat. Clim. Change* **6**(10), 946-949 (2016).
9. Ribeiro, A. F., Russo, A., Gouveia, C. M. & Pires, C. A. Drought-related hot summers: A joint probability analysis in the Iberian Peninsula. *Weather Clim. Extremes* **30**, 100279 (2020).
10. Schwingshackl, C., Sillmann, J., Vicedo-Cabrera, A. M., Sandstad, M. & Aunan, K. Heat stress indicators in CMIP6: estimating future trends and exceedances of impact-relevant thresholds. *Earth's Future* **9**, e2020EF001885 (2021).

11. Tabari, H. & Willems, P. Sustainable development substantially reduces the risk of future drought impacts. *Commun. Earth Environ.* **4**, 180 (2023).

12. Tabari, H. & Willems, P. Trivariate analysis of changes in drought characteristics in the CMIP6 multimodel ensemble at global warming levels of 1.5, 2, and 3 °C. *J. Clim.* **35**, 5823–5837 (2022).

13. Tabari, H. Extreme value analysis dilemma for climate change impact assessment on global flood and extreme precipitation. *J. Hydrol.* **593**, 125932 (2021).

14. Wu, X., Hao, Z., Zhang, X., Li, C. & Hao, F. Evaluation of severity changes of compound dry and hot events in China based on a multivariate multi-index approach. *J. Hydrol.* **583**, 124580 (2020).

15. Zscheischler, J. et al. Impact of large-scale climate extremes on biospheric carbon fluxes: An intercomparison based on MsTMIP data. *Global Biogeochem. Cycles* **28**, 585-600 (2014).
