# Supplementary material for: Multi-hazard exposure and vulnerability drive rising agricultural risk in global breadbasket regions
Source: Nat Commun. 2026 Jun 18;17:7706. doi: 10.1038/s41467-026-74617-5 (PMC13434463; doi:10.1038/s41467-026-74617-5)
Supplement: Supplementary file 2 [file 41467_2026_74617_MOESM2_ESM.pdf]

nature portfolio

Corresponding author(s): Hossein Tabari

Last updated by author(s): Apr 29, 2026

Reporting Summary

Nature Portfolio wishes to improve the reproducibility of the work that we publish. This form provides structure for consistency and transparency in reporting. For further information on Nature Portfolio policies, see our Editorial Policies and the Editorial Policy Checklist.

Statistics

For all statistical analyses, confirm that the following items are present in the figure legend, table legend, main text, or Methods section.

n/a	Confirmed	
☐	☒	The exact sample size (*n*) for each experimental group/condition, given as a discrete number and unit of measurement
☒	☐	A statement on whether measurements were taken from distinct samples or whether the same sample was measured repeatedly
☐	☒	The statistical test(s) used AND whether they are one- or two-sided<br>*Only common tests should be described solely by name; describe more complex techniques in the Methods section.*
☒	☐	A description of all covariates tested
☒	☐	A description of any assumptions or corrections, such as tests of normality and adjustment for multiple comparisons
☐	☒	A full description of the statistical parameters including central tendency (e.g. means) or other basic estimates (e.g. regression coefficient) AND variation (e.g. standard deviation) or associated estimates of uncertainty (e.g. confidence intervals)
☐	☒	For null hypothesis testing, the test statistic (e.g. *F*, *t*, *r*) with confidence intervals, effect sizes, degrees of freedom and *P* value noted<br>*Give P values as exact values whenever suitable.*
☒	☐	For Bayesian analysis, information on the choice of priors and Markov chain Monte Carlo settings
☒	☐	For hierarchical and complex designs, identification of the appropriate level for tests and full reporting of outcomes
☒	☐	Estimates of effect sizes (e.g. Cohen's *d*, Pearson's *r*), indicating how they were calculated

Our web collection on statistics for biologists contains articles on many of the points above.

Software and code

Policy information about availability of computer code

Data collection	NA
Data analysis	MATLAB R2021a was used for all analyses in this paper. The code developed in this study for calculating the PCFA-based vulnerability index is publicly available at https://doi.org/10.5281/zenodo.19844833.

For manuscripts utilizing custom algorithms or software that are central to the research but not yet described in published literature, software must be made available to editors and reviewers. We strongly encourage code deposition in a community repository (e.g. GitHub). See the Nature Portfolio guidelines for submitting code & software for further information.

Data

Policy information about availability of data

All manuscripts must include a data availability statement. This statement should provide the following information, where applicable:

- Accession codes, unique identifiers, or web links for publicly available datasets
- A description of any restrictions on data availability
- For clinical datasets or third party data, please ensure that the statement adheres to our policy

The CMIP6 global climate model outputs used to quantify droughts, heatwaves, and compound hot–dry events are available through the Earth System Grid Federation (ESGF) at https://esgf-node.ipsl.upmc.fr/projects/cmip6-ipsl/. Daily river discharge simulations used for flood assessment were obtained from the ISIMIP portal (https://www.isimip.org/). The AIM-SSP/RCP gridded emissions and land-use dataset is available at https://www-iam.nies.go.jp/aim/data_tools/aimssp/aimssp.html, and the Landuse 15 Crops dataset can also be accessed via ESGF. Population, age structure, educational attainment, and gender distribution data were

sourced from the International Institute for Applied Systems Analysis (IIASA) at https://iiasa.ac.at/models-tools-data. All processed data generated in this study are publicly available at Zenodo: https://doi.org/10.5281/zenodo.19844833.

Research involving human participants, their data, or biological material

Policy information about studies with human participants or human data. See also policy information about sex, gender (identity/presentation), and sexual orientation and race, ethnicity and racism.

Reporting on sex and gender	Not applicable. This study does not involve human participants, individual-level human data, or analyses based on sex or gender.
Reporting on race, ethnicity, or other socially relevant groupings	Not applicable. No human participants or individual-level demographic data were used.
Population characteristics	Not applicable. The study does not include human research participants.
Recruitment	Not applicable
Ethics oversight	Not applicable

Note that full information on the approval of the study protocol must also be provided in the manuscript.

Field-specific reporting

Please select the one below that is the best fit for your research. If you are not sure, read the appropriate sections before making your selection.

☐ Life sciences ☐ Behavioural & social sciences ☒ Ecological, evolutionary & environmental sciences

For a reference copy of the document with all sections, see nature.com/documents/nr-reporting-summary-flat.pdf

Ecological, evolutionary & environmental sciences study design

All studies must disclose on these points even when the disclosure is negative.

Study description	This study is based entirely on existing, publicly available simulation datasets; no new experiments were conducted. Climate hazards (droughts, heatwaves, and compound hot–dry events) were derived from CMIP6 outputs available via the Earth System Grid Federation (ESGF). Flood hazards were based on daily river discharge simulations from the ISIMIP framework. Land-use data were obtained from the AIM-SSP/RCP and LUH2 datasets, and socioeconomic indicators (population, age structure, education, and gender distribution) were sourced from IIASA.
Research sample	The study does not involve human or biological samples. The unit of analysis is spatially gridded data representing cropland exposure and associated socioeconomic indicators.
Sampling strategy	Not applicable. The analysis uses complete multi-model ensemble datasets rather than sampled observations.
Data collection	All data were obtained from publicly available databases (ESGF, ISIMIP, AIM-SSP/RCP, LUH2, and IIASA). No primary data collection was performed.
Timing and spatial scale	NA
Data exclusions	NA
Reproducibility	NA
Randomization	NA
Blinding	NA

Did the study involve field work? ☐ Yes ☒ No

Reporting for specific materials, systems and methods

We require information from authors about some types of materials, experimental systems and methods used in many studies. Here, indicate whether each material, system or method listed is relevant to your study. If you are not sure if a list item applies to your research, read the appropriate section before selecting a response.

Materials & experimental systems

n/a	Involved in the study
[x]	[ ] Antibodies
[x]	[ ] Eukaryotic cell lines
[x]	[ ] Palaeontology and archaeology
[x]	[ ] Animals and other organisms
[x]	[ ] Clinical data
[x]	[ ] Dual use research of concern
[x]	[ ] Plants

Methods

n/a	Involved in the study
[x]	[ ] ChIP-seq
[x]	[ ] Flow cytometry
[x]	[ ] MRI-based neuroimaging

Plants

Seed stocks	NA
Novel plant genotypes	NA
Authentication	NA
