# Supplementary material for: Multi-hazard exposure and vulnerability drive rising agricultural risk in global breadbasket regions
Source: Nat Commun. 2026 Jun 18;17:7706. doi: 10.1038/s41467-026-74617-5 (PMC13434463; doi:10.1038/s41467-026-74617-5)
Supplement: Supplementary file 3 [file 41467_2026_74617_MOESM3_ESM.pdf]

nature portfolio

Peer Review File

Multi-hazard exposure and vulnerability drive rising agricultural risk in global breadbasket regions

Corresponding Author: Professor Hossein Tabari

Parts of this Peer Review File have been redacted as indicated to remove third-party material.

This file contains all reviewer reports in order by version, followed by all author rebuttals in order by version.

Attachments originally included by the reviewers as part of their assessment can be found at the end of this file.

Version 0:

Reviewer comments:

Reviewer #1

(Remarks to the Author)
This study presents a global assessment of how climate extremes—specifically heatwaves, droughts, floods, and compound hot-dry events—threaten agricultural production across global cropland and ten major crop types. Using climate and hydrological model projections under multiple Shared Socioeconomic Pathways (SSPs), the authors quantify future cropland exposure and overlay it with an adaptive capacity index derived from six socio-institutional indicators using Principal Component Factor Analysis (PCFA). The results show that cropland exposure to climate extremes is projected to increase significantly, particularly in key breadbasket regions such as South Asia, Sub-Saharan Africa, and the United States. Cereal crops, especially maize and rice, are among the most exposed. A key finding is the emergence of a strong inverse relationship between adaptive capacity and exposure in future scenarios—regions with the highest exposure often have the lowest capacity to adapt. This pattern was not observed historically, indicating that future vulnerability will be increasingly shaped by socioeconomic and institutional readiness rather than just physical hazard exposure. The study concludes that safeguarding global food security under climate change requires not only reducing emissions and physical risks but also investing in adaptive capacity—particularly in vulnerable breadbasket regions. It calls for equity-focused, proactive adaptation strategies and highlights the importance of integrating social resilience into climate risk assessments.
This is a decent paper and huge datasets as inherent in any global study are a challenge. The findings also are interesting. However, I do have a few concerns and suggestion for improvement:
1. My biggest concern is the uncertainty and sensitivity of the results. There are so many inherent uncertainty within the studies – from the climate projections, to the future estimations of the four climate extremes, to the cropland/crop types dynamics in the future, to the adaptation capacities of different countries – that the projected changes in global cropland exposure to climate extremes may be even be smaller than the variabilities due to these uncertainties. This would make this study (and the findings) is irrelevant or even meaningless.

2. The authors emphasize a lot on their spatially explicit analysis, and yet the results are mostly on country level or even on regional (e.g. South Asia) level. Nothing particular or interesting are found or presented in e.g. sub-national levels (which would be the strength of spatially explicit analysis).
3. The use of CMIP6 and ISIMIP model outputs, along with harmonized land-use projections, lends credibility to the findings. However, while the manuscript effectively identifies correlations between adaptive capacity and exposure, it would benefit from a more explicit discussion of the causal mechanisms. For example, elaborating on how specific institutional or demographic factors mediate the relationship between exposure and vulnerability could strengthen the interpretation of results. Additionally, although the manuscript acknowledges the absence of crop yield modeling, it might be helpful to include a brief illustrative example or reference to existing literature that estimates yield losses under similar exposure conditions. This would help readers better understand the practical implications of increased exposure for food security.

---

4. The manuscript is well-organized and follows a logical progression from introduction to methods, results, and discussion. However, the results section, while rich in detail, can be overwhelming at times due to the volume of scenario-specific data. To improve readability, consider summarizing key findings in a table or schematic before delving into the specifics. This would allow readers to grasp the main messages more quickly and refer back to the summary as needed. The discussion

section is generally strong but could more explicitly tie back to the framing in the introduction. For instance, reiterating the implications of simultaneous breadbasket failures and their potential to destabilize global food systems would reinforce the urgency and relevance of your findings.

5. The emphasis on equity-focused adaptation and the role of institutional capacity is particularly relevant for decision-makers. To enhance the policy relevance further, consider adding a dedicated paragraph that outlines concrete recommendations for governments, development agencies, and multilateral institutions. These could include actions such as investing in climate-resilient infrastructure, strengthening governance systems, and supporting education and capacity-building in vulnerable regions.

Moreover, while the analysis is conducted at the national and global levels, a brief discussion of subnational disparities—such as differences within large countries like India or the United States—would make the findings more actionable. Highlighting the need for localized adaptation strategies could help bridge the gap between global assessments and on-the-ground policy implementation.

---

6. Minor Suggestions for Improvement: A brief validation or robustness check of the adaptive capacity index—perhaps by comparing it with existing indices—would add credibility to your approach. Second, expanding the discussion of mechanisms linking adaptive capacity to exposure outcomes would deepen the reader's understanding of the results. For example, you could explore how governance quality or education levels influence a region's ability to respond to climate shocks. Third, including a short "Policy Implications" box with 3–5 actionable recommendations would make the manuscript more useful for practitioners and policymakers.

Reviewer #2

(Remarks to the Author)
Thank you for the opportunity to review this study entitled "Compounding effects of high exposure and low adaptive capacity intensify climate risks in breadbasket regions".
Unfortunately, I have several major concerns regarding this study:
- Novelty: the originality of this work is unclear. The authors state that "Although significant progress has been made in understanding the impacts of long-term mean climate change, the ability to assess risks from these increasingly severe extremes remains limited". However, this claim is overly general and not entirely accurate, as numerous global studies have already examined crop-climate interactions. The present study appears to simply overlay projections of floods, heatwaves, droughts, and compound hot-dry events onto cropland. It is not evident how this relatively simplistic approach advances beyond existing studies that employ crop models and account for CO_2 fertilization, temperature, water availability, management practices, and crop growth stages.
- Adaptive capacity: The only potential innovative aspect is the inclusion of "adaptive capacity", which I do not recall being incorporated in previous studies. While promising, its derivation, rationale, validation, and influence on crop risk are insufficiently explained. The PCFA method, used to derive adaptive capacity, is a standard method in climate vulnerability and risk assessments but has inherent limitations: subjectivity in choosing indicators (here 6), number of factors, and interpretation none of which are discussed in the article. As currently framed, it is unclear how this metric meaningfully alters the risk assessment. In principle, crop modeling could reveal cases where regions with high exposure (i.e., to floods or droughts) might reduce risk due to low vulnerability, but such insights are absent here. Fig. 6, the only figure showing "adaptive-capacity", reduce adaptive capacity (in the form of the 6 indicators) and risk into country-level aggregates, then correlate them. That is a very coarse representation, and it doesn't really capture how adaptive capacity modulates crop risk under extremes.
- Use of the term "compounding": The title is misleading. i) At no point do the authors analyze interactions between the four extremes. Instead, the study presents spatial pattern of risk associated with each climate extreme separately toward the end of the century. ii) Looking specifically at climate extremes in relation to both exposure and adaptive capacity, such analysis is again not demonstrated. The figures (e.g., the network analysis) appear to show only correlations between aggregated country-level indicators and individual climate extremes, rather than interactions among extremes, exposure, and adaptive capacity. No evidence is provided that the combined or synergistic effects of these factors were quantified.
- Risk terminology: The study does not employ an established risk framework such as that of the IPCC. As a result, the terminology is unclear and at times used interchangeably, which weakens the conceptual foundation of the analysis.
- Discussion: The discussion section is overly general and descriptive, and it lacks quantitative metrics and uncertainty information. This further reinforces the impression that the study does not provide significant novelty.

As a result, I cannot recommend this study for publication. (see attached file for specific comments)

Reviewer #3

(Remarks to the Author)
This is an valuable and timely works that brings together climate extremes and adaptive capacity in a global agricultural vulnerability framework. The integration of multiple hazards with socio-institutional indicators is valuable, and the use of the existing model is innovative. The manuscript is clear and well organized, but several areas need improvement to strengthen its clarity, methodological robustness, and policy relevance. Paper can be accepted after addressing following major revision comments.

The research gap could be stated more explicitly. The introduction provides useful context but does not clearly explain how

this study advances beyond earlier work on compound extremes or adaptive capacity assessments.

The choice of six adaptive capacity indicators is reasonable, but the rationale for excluding other relevant factors such as infrastructure, inequality, or access to technology needs to be discussed. Even a brief sensitivity test (not compulsory but recommended) or acknowledgement of this limitation would increase confidence in the index.

The handling of model uncertainty deserves more attention. The paper draws on outputs from CMIP6 and ISIMIP, yet it is not clear how inter-model spread, bias correction, or weighting influenced the results. More transparency on these points would improve robustness.

The analysis is performed at 0.5° spatial resolution, which inevitably masks sub-national heterogeneity within breadbasket regions. This limitation should be acknowledged more explicitly, especially given the importance of local-level adaptation.

The adaptive capacity index, while methodologically interesting, would benefit from additional robustness comparison. For example, a comparison with existing vulnerability indices would help validate the results.

The focus is largely on breadbasket regions, yet intermediate regions also show substantial increases in exposure. These areas may play a buffering role in global supply chains, and a more balanced discussion of their significance would be good.

The policy implications remain quite general. The discussion would be stronger if it offered concrete examples of adaptation options tailored to the identified hotspots, such as crop diversification, irrigation efficiency, or regional trade measures.

Version 1:

Reviewer comments:

Reviewer #1

(Remarks to the Author)
Thank you for the careful and diligent revisions. I don't have any more comments and would recommend for acceptance of your paper. Congratulations!

Reviewer #3

(Remarks to the Author)
Authors have addressed the comments properly. The manuscript can be accepted for publication.

Open Access This Peer Review File is licensed under a Creative Commons Attribution 4.0 International License, which permits use, sharing, adaptation, distribution and reproduction in any medium or format, as long as you give appropriate

credit to the original author(s) and the source, provide a link to the Creative Commons license, and indicate if changes were made.
In cases where reviewers are anonymous, credit should be given to 'Anonymous Referee' and the source.
The images or other third party material in this Peer Review File are included in the article's Creative Commons license, unless indicated otherwise in a credit line to the material. If material is not included in the article's Creative Commons license and your intended use is not permitted by statutory regulation or exceeds the permitted use, you will need to obtain permission directly from the copyright holder.
To view a copy of this license, visit https://creativecommons.org/licenses/by/4.0/

Multi-hazard exposure and vulnerability drive rising agricultural risk in global breadbasket regions

Response to reviewers

November 28, 2025

Contents

1 Reviewer 1 5

Reviewer 1 General Comments 5

Reviewer 1 Comment 1........ 7

Reviewer 1 Comment 2........ 9

Reviewer 1 Comment 3........ 10

Reviewer 1 Comment 4........ 12

Reviewer 1 Comment 5........ 14

Reviewer 1 Comment 6........ 17

2 Reviewer 2 23

Reviewer 2 General Comments 23

Reviewer 2 Major comment 1 23

Reviewer 2 Major comment 2 26

Reviewer 2 Major comment 3 36

Reviewer 2 Major comment 4 40

Reviewer 2 Major comment 5 40

Reviewer 2 Specific comment 1........ 41

Reviewer 2 Specific comment 2........ 42

Reviewer 2 Specific comment 3 43
Reviewer 2 Specific comment 4 44
Reviewer 2 Specific comment 5 45
Reviewer 2 Specific comment 6 45
Reviewer 2 Specific comment 7 46
Reviewer 2 Specific comment 8 48
Reviewer 2 Specific comment 9 48
Reviewer 2 Specific comment 10 48
Reviewer 2 Specific comment 11 49
Reviewer 2 Specific comment 12 49
Reviewer 2 Specific comment 13 50
Reviewer 2 Specific comment 14 50
Reviewer 2 Specific comment 15 51
Reviewer 2 Specific comment 16 52
Reviewer 2 Specific comment 17 53
Reviewer 2 Specific comment 18 53
Reviewer 2 Specific comment 19 53
Reviewer 2 Specific comment 20 54
Reviewer 2 Specific comment 21 54
Reviewer 2 Specific comment 22 55
Reviewer 2 Specific comment 23 56
Reviewer 2 Specific comment 24 56
Reviewer 2 Specific comment 25 57
Reviewer 2 Specific comment 26 58

Reviewer 2 Specific comment 27 59

Reviewer 2 Specific comment 28 59

Reviewer 2 Specific comment 29 62

Reviewer 2 Specific comment 30 64

Reviewer 2 Specific comment 31 64

Reviewer 2 Specific comment 32 67

Reviewer 2 Specific comment 33 68

Reviewer 2 Specific comment 34 69

Reviewer 2 Specific comment 35 69

Reviewer 2 Specific comment 36 70

Reviewer 2 Specific comment 37 70

Reviewer 2 Specific comment 38 72

Reviewer 2 Specific comment 39 73

3 Reviewer 3 77

Reviewer 3 General Comments 77

Reviewer 3 Comment 1 78

Reviewer 3 Comment 2 80

Reviewer 3 Comment 3 85

Reviewer 3 Comment 4 88

Reviewer 3 Comment 5 89

Reviewer 3 Comment 6 90

Reviewer 3 Comment 7 91

Abstract

We express our sincere gratitude to all the reviewers for taking the time to review our manuscript and providing us with valuable and constructive feedback. We have taken great care in addressing all of their comments, which have significantly enhanced the quality of our work. In the following sections, we will provide a clear and detailed explanation of the modifications we have made in response to each reviewer's feedback. To facilitate readability, all comments are marked in grey boxes, and we have used the convention of denoting modifications in the original manuscript with blue color.

1 Reviewer 1

Reviewer 1 General Comments
This study presents a global assessment of how climate extremes—specifically heatwaves, droughts, floods, and compound hot-dry events—threaten agricultural production across global cropland and ten major crop types. Using climate and hydrological model projections under multiple Shared Socioeconomic Pathways (SSPs), the authors quantify future cropland exposure and overlay it with an adaptive capacity index derived from six socio-institutional indicators using Principal Component Factor Analysis (PCFA). The results show that cropland exposure to climate extremes is projected to increase significantly, particularly in key breadbasket regions such as South Asia, Sub-Saharan Africa, and the United States. Cereal crops, especially maize and rice, are among the most exposed. A key finding is the emergence of a strong inverse relationship between adaptive capacity and exposure in future scenarios—regions with the highest exposure often have the lowest capacity to adapt. This pattern was not observed historically, indicating that future vulnerability will be increasingly shaped by socioeconomic and institutional readiness rather than just physical hazard exposure. The study concludes that safeguarding global food security under climate change requires not only reducing emissions and physical risks but also investing in adaptive capacity—particularly in vulnerable breadbasket regions. It calls for equity-focused, proactive adaptation strategies and highlights the importance of integrating social resilience into climate risk assessments.<br>This is a decent paper and huge datasets as inherent in any global study are a challenge. The findings also are interesting. However, I do have a few concerns and suggestion for improvement:

Response

We sincerely thank the reviewer for their careful reading, constructive summary, and insightful suggestions, which have greatly improved the clarity and robustness of our manuscript. We carefully addressed all major concerns raised in the detailed comments as follows:

- **Uncertainty and sensitivity**: We explicitly quantified uncertainty across multiple dimensions (model, scenario, and vulnerability representation) and demonstrated the robustness of our findings through ensemble medians, inter-model spreads, and benchmarking of the PCFA-based vulnerability index against the ND-GAIN indices. The Discussion now includes a concise statement emphasizing that, while uncertainty is intrinsic to forward-looking assessments, the identified exposure patterns are consistent and statistically significant across models, crops, and SSP–RCP combinations.
- **Spatial detail**: We expanded the Results section to highlight subnational contrasts within key producing countries (e.g., the Indo-Gangetic Plain, U.S. Midwest, and southern Brazil), thereby clarifying the spatially explicit nature of our analysis.
- **Causal interpretation**: The Discussion now explicitly explains the mechanisms through which governance, education, and institutional capacity mediate the translation of physical hazards into agricultural risk, providing a clearer causal interpretation of the adaptive capacity–exposure relationship.
- **Policy relevance**: We expanded the concluding part of the Discussion to include concrete, region-specific recommendations for governments, development agencies, and multilateral organizations. These emphasize actionable pathways such as strengthening governance systems, investing in irrigation and early-warning infrastructure, and promoting climate-smart and equity-focused adaptation strategies.

Reviewer 1 Comment 1
My biggest concern is the uncertainty and sensitivity of the results. There are so many inherent uncertainty within the studies – from the climate projections, to the future estimations of the four climate extremes, to the cropland/crop types dynamics in the future, to the adaptation capacities of different countries – that the projected changes in global cropland exposure to climate extremes may be even be smaller than the variabilities due to these uncertainties. This would make this study (and the findings) is irrelevant or even meaningless.

Response

We thank the reviewer for raising this important point. We fully acknowledge that uncertainty is inherent in projecting future climate impacts on cropland exposure. However, such uncertainty does not render the results irrelevant or meaningless; rather, it highlights the need for transparent quantification and robustness assessment. As illustrated in the well-established cascade of uncertainty framework (e.g., see Figure RL1; RL: response letter), uncertainty naturally increases from emissions and climate modeling to impact-level projections. In this study, we explicitly embrace this uncertainty by systematically analyzing its main components and ensuring that our conclusions are supported by consistent, ensemble-based evidence across all scenarios.

Specifically, we accounted for and communicated uncertainty in multiple dimensions:

- **Model uncertainty** in total cropland exposure projections is explicitly represented in Figures 2 and 3 through inter-model spreads across the CMIP6 ensemble. This quantifies variability in the magnitude of exposure changes for each hazard and scenario, showing that despite variability, the ensemble medians consistently indicate substantial increases in exposure. Furthermore, model uncertainty in crop-specific exposure projections is addressed in Figure 3, which presents the inter-model spread of exposure changes for major crop types. The consistent signal of

increase across models and crops demonstrates that our findings are robust to individual model differences.

- **Scenario uncertainty** is systematically treated by evaluating distinct SSP–RCP combinations (SSP1-2.6, SSP2-4.5, SSP3-7.0, SSP4-6.0, SSP5-8.5) that capture a broad range of future emissions, socioeconomic, and land-use pathways. The differences between these scenarios are clearly described in the paper.
- **Sensitivity and validity** of the vulnerability framework were explicitly tested through benchmarking our Principal Component Factor Analysis (PCFA)-based vulnerability index against the established ND-GAIN indices. The strong cross-country correlation ($r = 0.881$, $p < 0.001$) and unbiased Bland–Altman results confirm both the validity and robustness of our vulnerability characterization.

Together, these analyses demonstrate that while uncertainty is inherent in forward-looking assessments, the signal of increasing cropland exposure and its strong dependence on socioeconomic pathways and vulnerability is consistent, statistically significant, and physically interpretable across all tests. We have added the below statement in the Discussion explicitly clarifying that the study's conclusions remain robust despite the quantified uncertainties.

"While all future impact assessments inevitably involve uncertainty, our analysis explicitly quantifies and communicates these uncertainties across multiple dimensions. Despite variability, ensemble medians consistently indicate significant increases in cropland exposure across hazards and regions, demonstrating that the identified patterns are robust across models, crops, and scenarios."

[REDACTED]

Figure RL 1. Cascade of uncertainty in impact assessments of climate change (Wilby and Dessai, 2010).

Reviewer 1 Comment 2

The authors emphasize a lot on their spatially explicit analysis, and yet the results are mostly on country level or even on regional (e.g. South Asia) level. Nothing particular or interesting are found or presented in e.g. sub-national levels (which would be the strength of spatially explicit analysis).

Response

While our risk and vulnerability analysis is constrained to the country level due to the availability of socio-institutional indicators, the exposure component of our framework is fully spatially explicit at the grid scale, as presented in Figure 1 and Supplementary Figure 1. We agree with the reviewer on the importance of presenting results at subnational levels, which is indeed a key strength of a spatially explicit analysis. To address this, we expanded the Results section by adding a paragraph that highlights subnational

contrasts within major crop-producing nations. This new text identifies key intra-country hotspots where cropland exposure to multiple extremes intensifies such as the Indo-Gangetic Plain in South Asia, the Midwest and southern Great Plains in the United States, and the southern states of Brazil (Paraná, Rio Grande do Sul, and Santa Catarina):

"Beyond global and regional aggregates, our gridded analysis (Fig. 1 and Supplementary Fig. 1) reveals pronounced subnational contrasts within major agricultural producers. The Indo-Gangetic Plain, spanning northern India, southern Nepal, eastern Pakistan and Bangladesh, consistently emerges as a multi-hazard hotspot, with cropland highly exposed to floods, droughts, heatwaves and compound hot-dry events. In the United States, elevated exposure to heatwaves, droughts and compound events is concentrated across the Midwest and southern Great Plains. In Brazil, exposure increases most sharply in the southern states (Paraná, Rio Grande do Sul and Santa Catarina), where cropland faces rising risks from all four extremes, whereas northern agricultural frontiers show an intensification of heat and compound events. These patterns demonstrate that exposure is far from spatially uniform, even within top crop-producing nations."

Reviewer 1 Comment 3

The use of CMIP6 and ISIMIP model outputs, along with harmonized land-use projections, lends credibility to the findings. However, while the manuscript effectively identifies correlations between adaptive capacity and exposure, it would benefit from a more explicit discussion of the causal mechanisms. For example, elaborating on how specific institutional or demographic factors mediate the relationship between exposure and vulnerability could strengthen the interpretation of results. Additionally, although the manuscript acknowledges the absence of crop yield modeling, it might be helpful to include a brief illustrative example or reference to existing literature that estimates yield

losses under similar exposure conditions. This would help readers better understand the practical implications of increased exposure for food security.

Response

We agree and added an explicit discussion of the mechanisms linking vulnerability to exposure outcomes in the Discussion section. The new paragraph explains how governance quality, education/extension, and coordination capacity mediate risk, either buffering or amplifying the translation of physical hazards into agricultural losses:

"The mechanisms underlying this relationship clarify how institutional and human capacities shape regional responses to climate shocks. Strong governance enables timely resource allocation for risk management such as maintaining irrigation and drainage, operating early-warning systems, coordinating disaster response, and enforcing land use regulations that limit exposure. Higher education and extension access enhance adaptive decision-making by improving risk awareness, facilitating technology adoption, and fostering innovation in farm management. By contrast, weak institutions and low human capital often lead to fragmented coordination and delayed or inadequate responses to extremes. These institutional and human dimensions therefore act as buffers or amplifiers of physical exposure, determining whether climate shocks translate into systemic agricultural losses."

As suggested, we also added a synthesis of existing literature to illustrate the practical implications of increased exposure for food security:

"Previous global crop modeling studies for future climates have predominantly assessed the effects of mean global warming or CO_2 fertilization[12,14,15,27,31] rather than future climate extremes[21,24]. These studies consistently report average yield reductions of 6.0% for wheat, 3.2% for rice, 7.4% for maize, and 3.1% for soybean per degree of global warming[54,55], and project a 24% decline in global maize production by the

end of the century under SSP5-8.5[21]. For the historical period, Heinicke et al.[56] evaluated 13 crop yield models and found mean relative yield declines under drought of 4.3% (maize), 1.3% (rice), 3.4% (soybean), and 6.7% (wheat), and under heatwaves of 8.2% (maize), 2.2% (soybean), and 4.5% (wheat). Empirical analyses of national cereal production likewise show 9–10% losses during droughts and extreme heat[57]. Yield losses are most pronounced when high temperatures coincide with dry conditions (compound hot–dry events), particularly during sensitive growth stages[48,58-60], with temperature effects further modulated by precipitation[61] and soil moisture[62]. For example, during the 2003 European compound heatwave and drought event, wheat and maize yields declined by 11% and 21%, respectively[63]. Zhao et al.[49] also show that compound hot–dry–windy events are particularly damaging to U.S. wheat, causing an average 4% reduction per 10 hours of exposure during heading to maturity. Building on this historical and modeling evidence, the projected increases in cropland exposure to climate extremes identified here are therefore expected to translate into substantial yield reductions, especially in major breadbasket regions. While all future impact assessments inevitably involve uncertainty, our analysis explicitly quantifies and communicates these uncertainties across multiple dimensions. Despite variability, ensemble medians consistently indicate significant increases in cropland exposure across hazards and regions, demonstrating that the identified patterns are robust across models, crops, and scenarios."

Reviewer 1 Comment 4
The manuscript is well-organized and follows a logical progression from introduction to methods, results, and discussion. However, the results section, while rich in detail, can be overwhelming at times due to the volume of scenario-specific data. To improve readability, consider summarizing key findings in a table or schematic before delving into the specifics. This would allow readers to grasp the main messages more quickly and refer back to the summary as needed. The discussion section is generally

strong but could more explicitly tie back to the framing in the introduction. For instance, reiterating the implications of simultaneous breadbasket failures and their potential to destabilize global food systems would reinforce the urgency and relevance of your findings.

Response

In Nature Communications, the final paragraph of the Introduction already serves to summarize the main findings and guide the reader into the Results. Adding a second summary immediately afterward would lead to back-to-back redundancy and reduce clarity without providing additional value. Instead, we have revised the final paragraph of the Introduction to more clearly synthesize the key results and improve the logical flow of the paper. The revised paragraph now reads as follows:

"Here, we address these gaps by developing the first global risk assessment of agricultural crops that integrates multi-hazard exposure and socioeconomic vulnerability. We quantify the exposure of total cropland and ten major crop groups to four key hazards (heatwaves, droughts, floods, and compound hot–dry events) under multiple future socioeconomic and land-use scenarios. The projected cropland exposure is compared among farming regions (breadbaskets, intermediate, and limited farming zones, defined by their cropland fraction). We then develop a vulnerability index using unsupervised latent factor modeling that objectively weights and synthesizes multidimensional demographic, development, and governance indicators into interpretable resilience dimensions (see Methods). By integrating this index with cropland exposure to climate extremes, we assess the risk of farming regions to future climate shocks. Our results show that breadbasket regions are projected to experience the largest increases in cropland exposure to climate extremes, with particularly strong signals in South Asia, Sub-Saharan Africa, and the United States. Differences across farming regions are driven more by climate change than by changes in cropland area. A statistically significant relationship between vulnerability and cropland exposure emerges in future

scenarios but is absent historically, indicating a growing role for vulnerability in shaping future agricultural risk."

In addition, as suggested by the reviewer, we strengthened the Discussion by explicitly reconnecting the findings to the framing in the Introduction. Specifically, we now emphasize the implications of concurrent or compounding breadbasket disruptions for global food-system stability and the cascading risks they pose to international trade and food security. The revised text includes the following addition:

"The spatial convergence of high exposure and high vulnerability identified in this study underscores the potential for simultaneous disruptions across major breadbasket regions. Such co-occurring or sequential failures could amplify volatility in global food markets, threaten trade-dependent nations, and strain humanitarian response systems. By linking physical climate risks with socio-institutional capacity, our analysis provides a framework to anticipate where global food system instability may first emerge under intensifying extremes. In doing so, this study delivers the first global, multi-hazard assessment that explicitly integrates spatially explicit cropland exposure with a validated, data-driven vulnerability index, thereby revealing future exposure–vulnerability relationships that are absent in the historical period."

Reviewer 1 Comment 5
The emphasis on equity-focused adaptation and the role of institutional capacity is particularly relevant for decision-makers. To enhance the policy relevance further, consider adding a dedicated paragraph that outlines concrete recommendations for governments, development agencies, and multilateral institutions. These could include actions such as investing in climate-resilient infrastructure,

strengthening governance systems, and supporting education and capacity-building in vulnerable regions.

Moreover, while the analysis is conducted at the national and global levels, a brief discussion of subnational disparities—such as differences within large countries like India or the United States—would make the findings more actionable. Highlighting the need for localized adaptation strategies could help bridge the gap between global assessments and on-the-ground policy implementation.

Response

We expanded the Discussion to emphasize the need for finer-scale vulnerability assessments that capture localized adaptive capacity and intra-country differences. The revised text now reads:

"Similar to our exposure analysis at grid scale (Fig. 1), future research could also explore vulnerability at finer spatial scales to capture subnational disparities and localized adaptation potential. Because vulnerability is computed at the national scale, substantial within-country heterogeneity in socioeconomic and institutional conditions is not represented. Developing subnational vulnerability indices (e.g., at state/province/district levels) that incorporate region-specific determinants, such as irrigation and drainage access, market connectivity and post-harvest storage capacity, agricultural extension services, and access to credit/insurance, would allow the index to reflect local adaptive capacity rather than national averages. This finer resolution would reveal within-country risk hotspots in major producers (e.g., the Indo-Gangetic Plain in India, the U.S. Midwest/Mississippi Basin, and MATOPIBA in Brazil) and better align risk assessment with jurisdictions that implement adaptation. Such refinement would support targeted, equity-focused planning, by directing resources to places and populations that are both highly exposed and least able to cope. In addition, several vulnerability indicators such as governance-related metrics (e.g., institutional capacity and policy implementation effectiveness) are difficult to quantify

systematically and often rely on qualitative assessments, which introduces further uncertainty into the vulnerability estimates."

In addition, we revised the concluding paragraph to provide concrete adaptation and policy recommendations for governments, development agencies, and multilateral organizations, as follows:

"Governments, development agencies, and multilateral institutions should therefore prioritize context-specific and inclusive adaptation actions in the regional hotspots identified by emerging risk assessments. In South Asia, investments in irrigation efficiency, groundwater recharge, and drought-resilient crop varieties can buffer yield losses associated with recurrent heat and water stress. Expanding access to climate-smart irrigation and precision agriculture technologies will also enhance long-term water-use sustainability. In Sub-Saharan Africa, where limited institutional capacity exacerbates exposure, priority should be given to crop diversification, small-scale water storage, and farmer-led insurance and credit systems. These approaches strengthen resilience by stabilizing production, improving local food access, and supporting smallholder autonomy under climate uncertainty. In the United States, exposure is driven primarily by climate change rather than structural adaptive deficits. Adaptation should therefore focus on regional trade adjustments, modernization of irrigation infrastructure, and flexible water-allocation policies aligned with state and federal adaptation plans.

Across all breadbasket regions, accelerating early-warning systems, agricultural extension services, and climate-informed decision-support tools will be essential to translate adaptive capacity into tangible risk reduction. Evidence shows that early-warning investments yield "triple dividends"[78] by saving lives, reducing economic losses, and strengthening long-term resilience particularly when linked to community-based and digital advisory platforms. Crucially, coordinating adaptation across multi-breadbasket systems via interoperable early-warning and market information platforms, contingency trade arrangements, and

regional risk-pooling is essential to limit cascading food insecurity risks and dampen systemic shocks to global supply chains.

Finally, because investments in capacity building and institutional strength often exhibit a temporal lag before reducing exposure, early, sustained, and well-targeted implementation is crucial. Safeguarding global food security under climate change thus requires a dual strategy: reducing physical hazards through technological and ecological innovation, while advancing socio-institutional transformation that directs financial, informational, and infrastructural resources toward those who are most exposed and have the weakest adaptive capacity."

Reviewer 1 Comment 6
A brief validation or robustness check of the adaptive capacity index—perhaps by comparing it with existing indices—would add credibility to your approach. Second, expanding the discussion of mechanisms linking adaptive capacity to exposure outcomes would deepen the reader's understanding of the results. For example, you could explore how governance quality or education levels influence a region's ability to respond to climate shocks. Third, including a short "Policy Implications" box with 3–5 actionable recommendations would make the manuscript more useful for practitioners and policymakers.

Response

Following the suggestion, we performed a validation analysis to evaluate the robustness of our adaptive capacity index. Specifically, we compared the adaptive capacity component of our PCFA-based vulnerability index with the Notre Dame Global Adaptation Initiative (ND-GAIN) vulnerability index, one of the most comprehensive and widely used global indicators of socioeconomic vulnerability that is available only for the historical period. The comparison shows high consistency in both spatial patterns and country-level correlations, confirming that our representation of adaptive capacity aligns with

established assessments. The results are now presented in Supplementary Figs. S9–S10, and the following text was added to the Results and Methods sections, respectively:

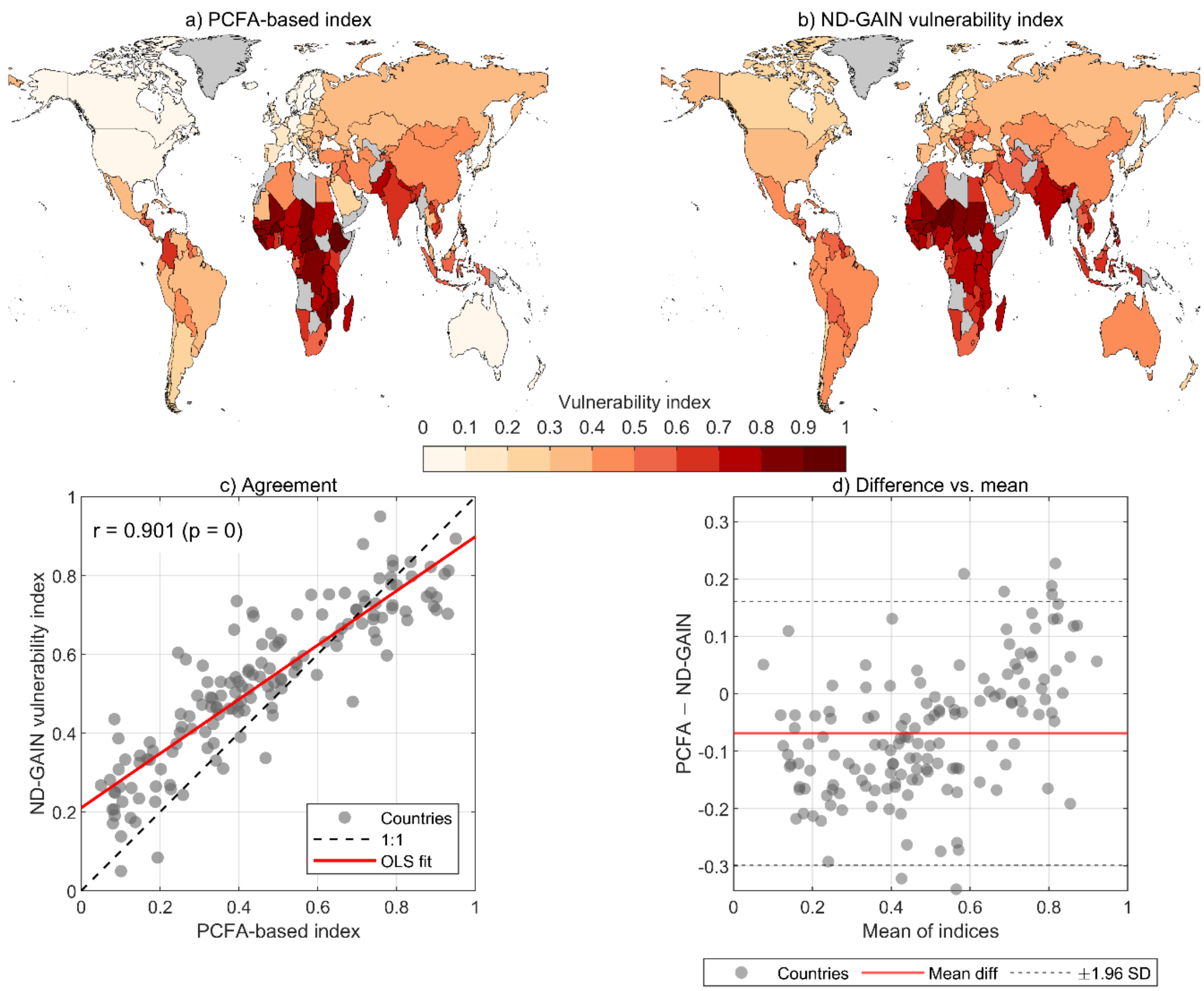

Supplementary Fig. 9 | Validation of the PCFA-based vulnerability index for the food sector. (a,b) Global maps of the PCFA-based vulnerability index (this study) and the ND-GAIN food-sector vulnerability for the historical period. (c) Country-level comparison with the 1:1 line (dashed) and ordinary least squares (OLS) fit (red). (d) Bland–Altman plot showing differences (PCFA − ND-GAIN) centered near zero and contained within ±1.96 SD.

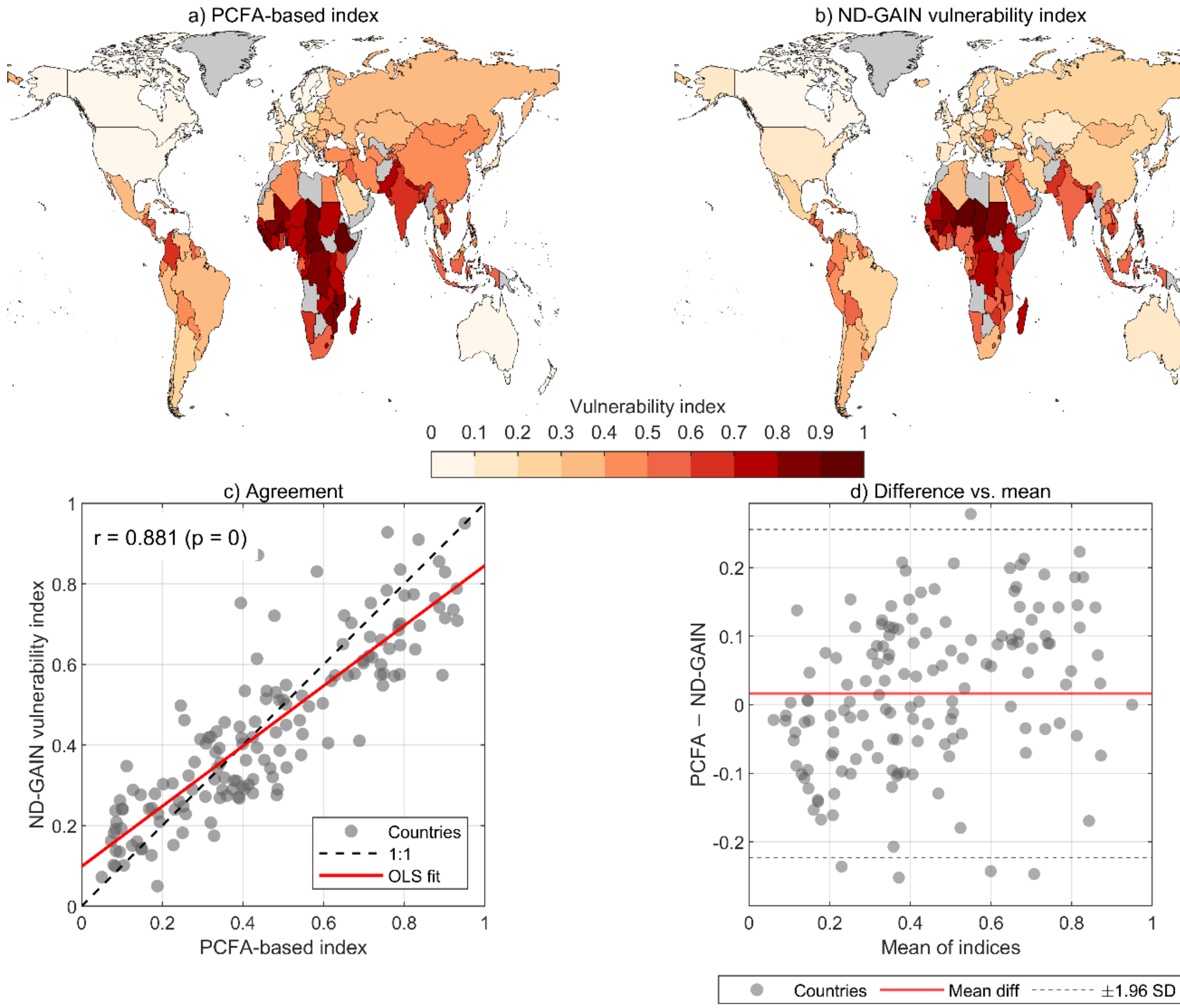

Supplementary Fig. 10 | Validation of the PCFA-based vulnerability index against overall ND-GAIN vulnerability (all sectors). (a,b) Global maps of the PCFA-based vulnerability index and the overall ND-GAIN vulnerability (all sectors) for the historical period. (c) Country-level comparison with the 1:1 line (dashed) and OLS fit (red). (d) Bland–Altman plot showing differences centered near zero and contained within ±1.96 SD.

"To assess the robustness, we benchmarked the PCFA vulnerability index against the Notre Dame Global Adaptation Initiative (ND-GAIN), a widely used and comprehensive composite of socioeconomic vulnerability that lacks future projections. Compared with the ND-GAIN food-sector vulnerability, spatial patterns align across countries (Supplementary Fig. S9a,b) and a strong linear association emerges in the

country-by-country comparison (r = 0.881, p < 0.001), with most countries near the 1:1 line (Supplementary Fig. S9c). A Bland–Altman analysis shows differences cantered near zero and bounded within ±1.96 SD with no trend versus the mean (Supplementary Fig. S9d), indicating no systematic bias. To test generalizability beyond the food sector, we compare the PCFA index with overall ND-GAIN vulnerability (36 indicators across six sectors); agreement remains high in both spatial patterns and cross-country correlations (Supplementary Fig. S10), with similarly small, unbiased differences in Bland–Altman space. Consistency with both sector-specific and overall ND-GAIN indices indicates that the PCFA index captures core structural dimensions of socioeconomic vulnerability and supports its use for future projections."

"To evaluate the robustness and representativeness of the PCFA-based vulnerability index, we compared it against the Notre Dame Global Adaptation Initiative (ND-GAIN)[114] vulnerability index, which is among the most comprehensive and widely used indicators of socioeconomic vulnerability. The ND-GAIN index is available at the country level for the historical period and quantifies vulnerability across six sectors (food, water, health, ecosystem services, human habitat, and infrastructure) based on 36 indicators. For consistency, we compared the PCFA index first with the ND-GAIN food-sector vulnerability, which is most directly related to agricultural and food system sensitivity, and subsequently with the overall ND-GAIN vulnerability encompassing all sectors. Validation was conducted through correlation and regression analyses to assess agreement, and (ii) Bland–Altman analysis to evaluate systematic bias and limits of agreement."

We also expanded the Discussion to further elaborate on the mechanisms linking adaptive capacity to exposure outcomes:

"The mechanisms underlying this relationship clarify how institutional and human capacities shape regional responses to climate shocks. Strong governance enables timely resource allocation for risk management such as maintaining irrigation and drainage, operating early-warning systems, coordinating disaster response, and enforcing land use regulations that limit exposure. Higher education and extension access enhance adaptive decision-making by improving risk awareness, facilitating technology adoption, and fostering innovation in farm management. By contrast, weak institutions and low human capital often lead to fragmented coordination and delayed or inadequate responses to extremes. These institutional and human dimensions therefore act as buffers or amplifiers of physical exposure, determining whether climate shocks translate into systemic agricultural losses."

Finally, we agree that summarizing key actionable insights enhances the manuscript's practical value. Accordingly, we added a "Policy Implications" box at the end of the Discussion that synthesizes key recommendations for practitioners and policymakers:

Policy Implications:

- ***Invest*** *in climate-resilient infrastructure (e.g., irrigation networks, on-farm storage, drainage, and floodplain management) to reduce direct exposure in high-risk regions.*
- ***Strengthen*** *governance and institutional coordination between national climate services and local agencies to improve risk communication and adaptive response.*
- ***Enhance*** *education, extension, and digital advisory systems to translate climate information into adaptive action at the farm level.*
- ***Expand*** *access to finance and insurance to buffer farmers against income shocks and enable recovery.*
- ***Prioritize*** *adaptation finance for regions where high exposure coincides with low adaptive capacity, ensuring equity in resilience-building.*

Reference

Wilby, R. L. & Dessai, S. Robust adaptation to climate change. Weather 65, 180–185 (2010).

2 Reviewer 2

Reviewer 2 General Comments
Thank you for the opportunity to review this study entitled “Compounding effects of high exposure and low adaptive capacity intensify climate risks in breadbasket regions”.<br>Unfortunately, I have several major concerns regarding this study:

Response

We thank the reviewer for carefully evaluating our work and for raising several important points that helped us strengthen both the conceptual framing and the clarity of our contribution. Below we respond to each concern in detail. Importantly, in revising the manuscript we ensured that the innovations and quantitative advances of this study are more clearly articulated in the Introduction, Results, and Discussion, and that uncertainty and methodological transparency are fully addressed.

Reviewer 2 Major comment 1
- Novelty: the originality of this work is unclear. The authors state that “Although significant progress has been made in understanding the impacts of long-term mean climate change, the ability to assess risks from these increasingly severe extremes remains limited”. However, this claim is overly general and not entirely accurate, as numerous global studies have already examined crop-climate interactions. The present study appears to simply overlay projections of floods, heatwaves, droughts, and compound hot-dry events onto cropland. It is not evident how this relatively simplistic approach advances beyond existing studies that employ crop models and account for CO2 fertilization, temperature, water availability, management practices, and crop growth stages.

Response

We appreciate the reviewer's comment and the opportunity to clarify how our study advances beyond existing crop–climate analyses. While numerous global studies have examined gradual climate change impacts, typically focusing on mean temperature, precipitation, and CO_2, using process-based or statistical crop models, these approaches primarily assess yield sensitivity to mean climate forcing. In contrast, our study adopts a risk-based, multi-hazard framework centered on climate extremes (heatwaves, droughts, floods, and compound hot–dry events) which are among the most critical yet least represented drivers of agricultural disruption and risk amplification.

Importantly, our analysis is underpinned by new, high-resolution projections of four types of climate extremes, derived from ensembles of climate and hydrological models under multiple future scenarios. Producing these multi-hazard projections in a consistent global framework required extensive, hazard-specific statistical modeling and validation, resulting in a unique, internally consistent suite that captures both single and compound extremes. To our knowledge, this represents the first global effort to jointly quantify the future exposure of cropland and crop types to multiple climate hazards, including compound events, within a unified modeling framework.

Beyond exposure mapping, our approach explicitly integrates the socioeconomic dimension of risk. Rather than modelling physiological yield responses, we quantify where and how much cropland and crop types will be exposed to multiple hazards and combine this with vulnerability and adaptive capacity indicators derived through latent-factor modeling. This integration enables us to identify true risk hotspots, where physical exposure and limited coping capacity intersect, an aspect not addressed by previous studies.

Thus, our contribution is both methodological and conceptual. Methodologically, we generate global multi-hazard projections, including compound extremes, from a large ensemble of climate and hydrological models, providing a unique dataset for the community. We then develop a PCFA-based

vulnerability index and integrate it with exposure to quantify agricultural risk. Conceptually, we bridge physical hazard exposure with socioeconomic vulnerability, moving beyond impact quantification toward a risk-informed resilience assessment. This provides actionable insight for prioritizing regions and crop systems where detailed process-based crop modeling or targeted adaptation efforts are most needed.

We revised the Introduction to explicitly state the study's novelty as the first global risk assessment of agricultural crops that integrates multi-hazard exposure and socioeconomic vulnerability.

"Over the past decade, major advances have been made in quantifying the impacts of gradual warming and shifting climate patterns on agricultural productivity. Process-based crop models have been used to project yields under different global warming levels[14], rising atmospheric CO_2[15], and regional climate conditions[16-20]. Global multi-model ensembles now estimate mean yield responses for major crops under various emission scenarios[21]. Similarly, empirical approaches link historical crop yields to observed climate anomalies[1,5,22-26] or explore adaptation measures such as adjusting sowing dates and cultivar maturity classes[27], as well as breeding advances[28,29]. These studies have provided valuable insights into long-term changes in crop productivity under gradual climate forcing.

Yet major gaps remain. Most global process-based assessments of agricultural impacts of climate change[14,15,21,23,30,31,32] focus on mean temperature, precipitation, and CO_2 trends or on simple anomalies rather than explicitly quantifying climate extremes (floods, droughts, and heatwaves) that directly drive agricultural losses. In addition, global empirical and statistical analyses typically consider only one hazard at a time[33-35], and future multi-hazard assessments, especially those that include compound events across diverse crop types, are still missing. The absence of such a framework limits understanding of how different hazards and their concurrent or sequential interactions, such as compound hot–dry events, exacerbate agricultural impacts[4,24,36]. Moreover, global crop risk analyses rarely include vulnerability, despite evidence that socioeconomic and institutional conditions strongly modulate impacts[37,38]. As a result, true global

agricultural risk hotspots, where high exposure coincides with limited adaptive capacity, remain poorly identified."

Reviewer 2 Major comment 2
- Adaptive capacity: The only potential innovative aspect is the inclusion of "adaptive capacity", which I do not recall being incorporated in previous studies. While promising, its derivation, rationale, validation, and influence on crop risk are insufficiently explained. The PCFA method, used to derive adaptive capacity, is a standard method in climate vulnerability and risk assessments but has inherent limitations: subjectivity in choosing indicators (here 6), number of factors, and interpretation none of which are discussed in the article. As currently framed, it is unclear how this metric meaningfully alters the risk assessment. In principle, crop modeling could reveal cases where regions with high exposure (i.e., to floods or droughts) might reduce risk due to low vulnerability, but such insights are absent here. Fig. 6, the only figure showing "adaptive-capacity", reduce adaptive capacity (in the form of the 6 indicators) and risk into country-level aggregates, then correlate them. That is a very coarse representation, and it doesn't really capture how adaptive capacity modulates crop risk under extremes.

Response

We thank the reviewer for this thoughtful comment, which helped us improve the clarity and justification of the adaptive capacity/vulnerability component within our risk framework. We fully agree that the derivation, rationale, validation, and influence of this metric need to be transparent, and we substantially strengthened these aspects in the revised manuscript.

1. Conceptual rationale and methodological transparency

Adaptive capacity reflects the socioeconomic and institutional conditions that shape how agricultural systems prepare for, respond to, and recover from climate extremes. The six indicators used in this study (governance quality, Human Development Index, tertiary education attainment, gender ratio, age dependency ratio, and population size) were selected because (i) they capture core dimensions of socioeconomic vulnerability identified in the literature, and (ii) they are among the few indicators for which consistent future projections exist under all SSPs. This ensures methodological continuity between historical and future assessments.

To synthesize these indicators, we implemented a Principal Component–Factor Analysis (PCFA) approach. PCFA combines the dimensionality-reduction strengths of PCA with the latent-structure interpretation of factor analysis, allowing us to derive an internally consistent, statistically grounded vulnerability index without imposing arbitrary or equal weights. We have expanded the Methodology section to provide a clearer explanation of how factor loadings, uniqueness, and factor scores were obtained and why this hybrid approach is well suited for multi-indicator vulnerability assessment.

2. Addressing methodological subjectivity and validating the approach

We acknowledge the reviewer's concern that indicator-based indices inevitably carry some subjectivity regarding indicator selection and the number of latent factors. We now explicitly discuss these limitations and provide two forms of validation.

First, we benchmark the PCFA-based index against the Notre Dame Global Adaptation Initiative (ND-GAIN) vulnerability index, which is one of the most widely used and comprehensive global indicators of socioeconomic vulnerability. As shown in Supplementary Figs. S9–S10, the PCFA index shows strong agreement with ND-GAIN in both spatial patterns and cross-country ranking ($r = 0.881$, $p < 0.001$), with Bland–Altman differences centered near zero. This demonstrates that our approach captures the key

structural dimensions of vulnerability represented in ND-GAIN while enabling forward projections under SSPs.

Second, following the reviewer's suggestion, we added an internal robustness test using a 2-factor PCFA model. As described in the revised Methods and Supplementary Fig. S13, the second factor increases the explained variance but does not materially change country rankings (all Spearman correlations significant at $p < 0.01$). This confirms that the 1-factor index captures the dominant vulnerability gradient and is sufficient for the purposes of this analysis.

3. Influence of adaptive capacity on agricultural risk

We agree that the original submission did not sufficiently illustrate how adaptive capacity modifies risk. To address this, we added a new analysis (Fig. 6) that explicitly stratifies the exposure–risk relationship by vulnerability quartiles across all hazards and scenarios. The systematically steeper slopes from low- to high-vulnerability quartiles show that for the same physical exposure, countries with lower adaptive capacity experience substantially higher risk. This demonstrates that vulnerability/adaptive capacity meaningfully alters the risk outcomes and is not a passive or redundant component of the framework.

Key revisions implemented

Methods (Assessing vulnerability and risk):

"Following IPCC AR5 risk framework[109], we defined risk as a function of hazard, exposure, and vulnerability/coping-capacity."

"We selected these indicators because future projections are available under SSPs 1–5 and they robustly capture socio-demographic and institutional dimensions of vulnerability, as detailed below."

"We began by applying PCA to the standardized indicator matrix to extract the dominant latent dimension. The first principal component (PC1), which explained between 46% and 55% of the total variance across scenarios (Supplementary Fig. 12), was used to derive the initial factor loadings for the PCFA. These loadings formed the basis for estimating each indicator's uniqueness (the proportion of variance not explained by the common factor), by evaluating the residual covariance after accounting for the initial factor structure. To validate the robustness of the 1-factor PCFA vulnerability index, we compared its results with those from a 2-factor composite vulnerability index. For this, we estimated a 2-factor PCFA model and combined the standardized factor scores from the first and second factors using weights proportional to the variance explained by each factor in the PCA step, so that the composite metric reflects both dominant and secondary vulnerability gradients. The resulting 2-factor composite index was then normalized to the same scale as the original 1-factor PCFA index. While the 2-factor solution explained a larger share of variance (65%–76% across scenarios) (Supplementary Fig. 12), the resulting 2-factor PCFA index yielded very similar vulnerability patterns to the 1-factor index, with high and statistically significant correlations for all scenarios (Supplementary Fig. 13). We therefore retain the 1-factor PCFA vulnerability index as the main metric used in the analysis."

"To evaluate the robustness and representativeness of the PCFA-based vulnerability index, we compared it against the Notre Dame Global Adaptation Initiative (ND-GAIN)[114] vulnerability index, which is among the most comprehensive and widely used indicators of socioeconomic vulnerability. The ND-GAIN index is available at the country level for the historical period and quantifies vulnerability across six sectors (food, water, health, ecosystem services, human habitat, and infrastructure) based on 36 indicators. For consistency, we compared the PCFA index first with the ND-GAIN food-sector vulnerability, which is most directly related to agricultural and food system sensitivity, and subsequently with the overall ND-GAIN vulnerability encompassing all sectors. Validation was conducted through correlation and regression

analyses to assess agreement, and (ii) Bland–Altman analysis to evaluate systematic bias and limits of agreement.”

“Risk was calculated as the product of hazard, exposure and vulnerability. Changes in risk were then quantified as the ratio between risk projected under each SSP scenario, using future hazard, exposure and vulnerability, and historical risk based on historical hazard, exposure and vulnerability.”

Results (Validation):

“To assess the robustness, we benchmarked the PCFA vulnerability index against the Notre Dame Global Adaptation Initiative (ND-GAIN), a widely used and comprehensive composite of socioeconomic vulnerability that lacks future projections. Compared with the ND-GAIN food-sector vulnerability, spatial patterns align across countries (Supplementary Fig. S9a,b) and a strong linear association emerges in the country-by-country comparison ($r = 0.881$, $p < 0.001$), with most countries near the 1:1 line (Supplementary Fig. S9c). A Bland–Altman analysis shows differences cantered near zero and bounded within ±1.96 SD with no trend versus the mean (Supplementary Fig. S9d), indicating no systematic bias. To test generalizability beyond the food sector, we compare the PCFA index with overall ND-GAIN vulnerability (36 indicators across six sectors); agreement remains high in both spatial patterns and cross-country correlations (Supplementary Fig. S10), with similarly small, unbiased differences in Bland–Altman space. Consistency with both sector-specific and overall ND-GAIN indices indicates that the PCFA index captures core structural dimensions of socioeconomic vulnerability and supports its use for future projections.”

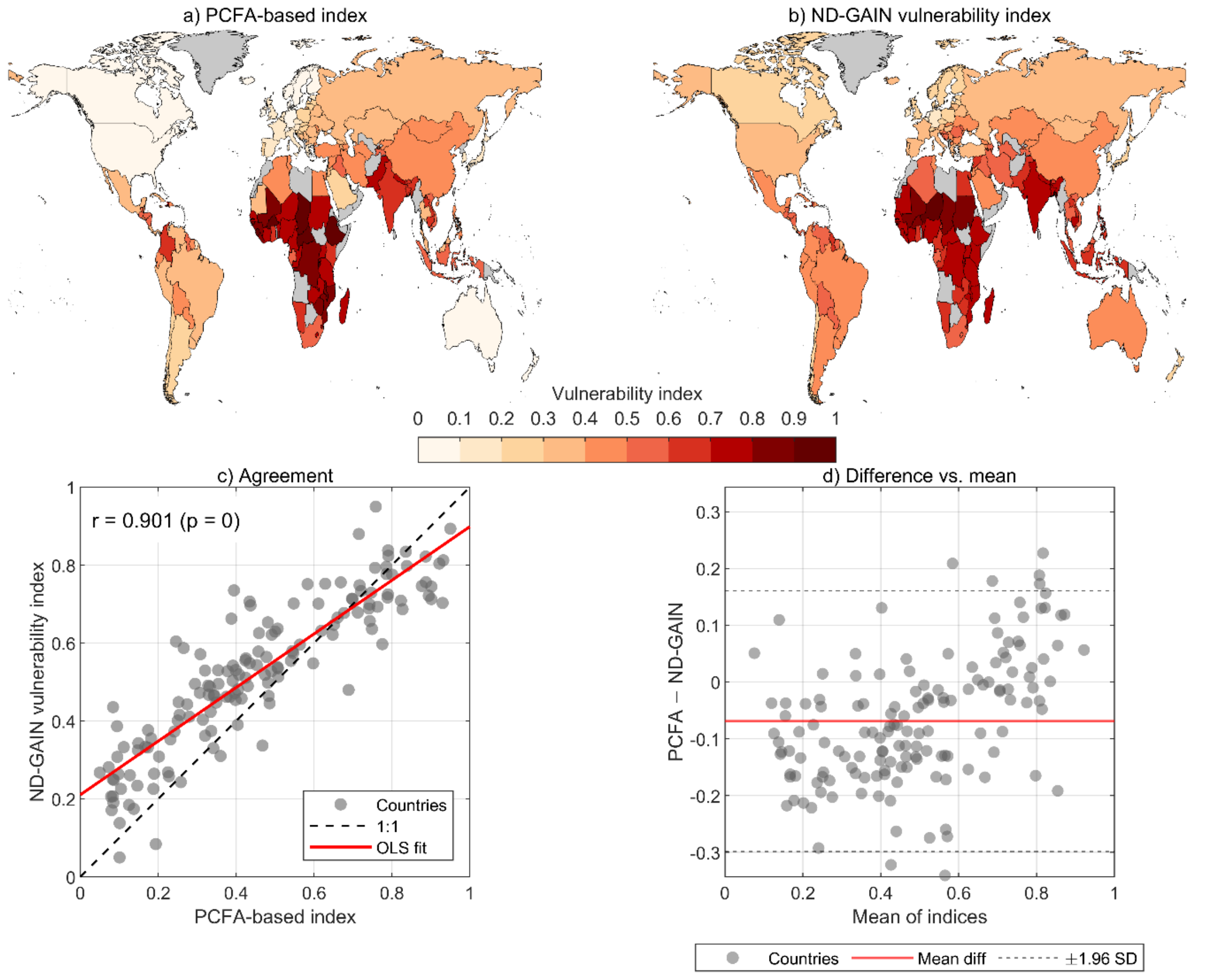

Supplementary Fig. 9 | Validation of the PCFA-based vulnerability index for the food sector. (a,b) Global maps of the PCFA-based vulnerability index (this study) and the ND-GAIN food-sector vulnerability for the historical period. (c) Country-level comparison with the 1:1 line (dashed) and ordinary least squares (OLS) fit (red). (d) Bland–Altman plot showing differences (PCFA − ND-GAIN) centered near zero and contained within ±1.96 SD.

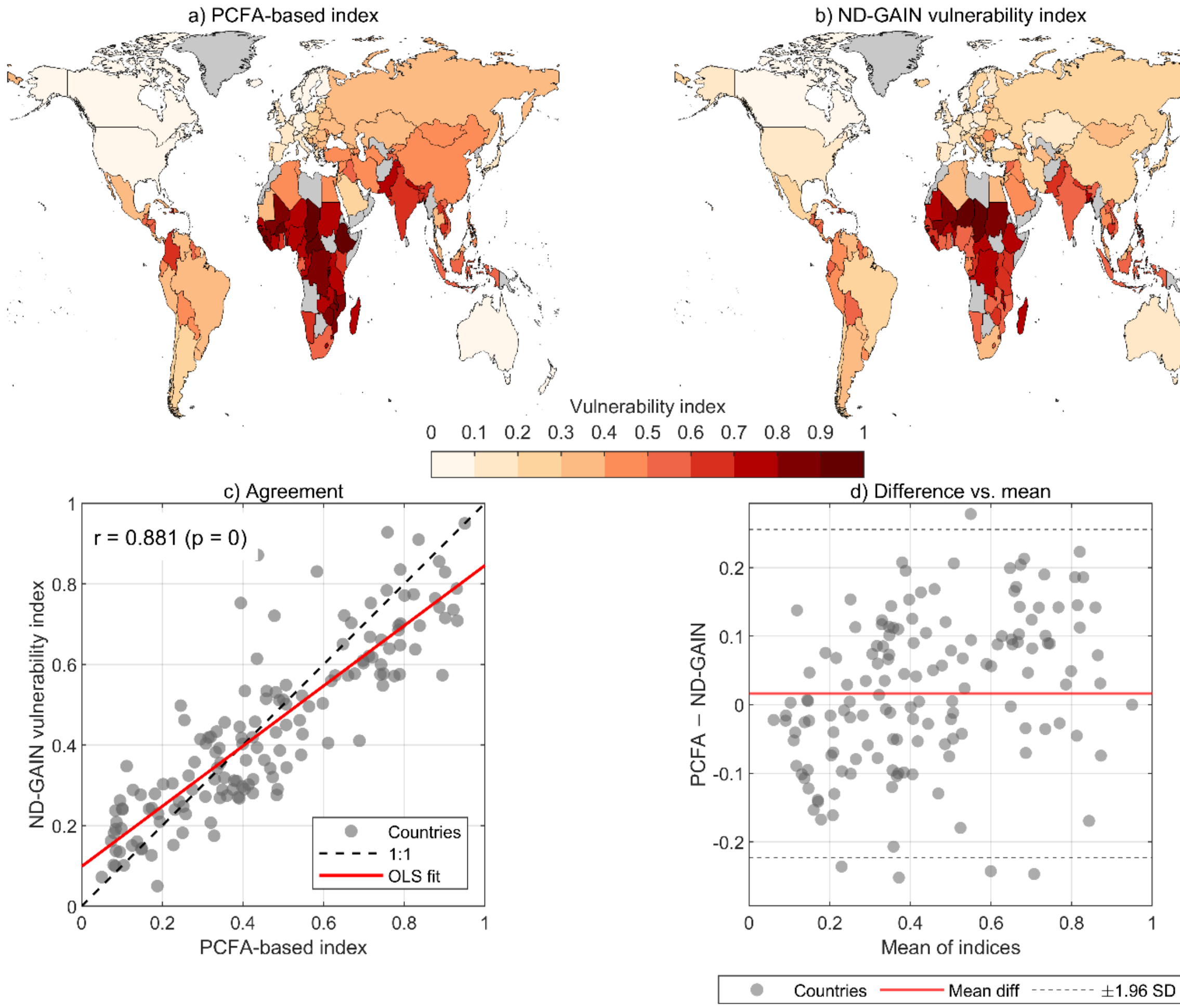

Supplementary Fig. 10 | Validation of the PCFA-based vulnerability index against overall ND-GAIN vulnerability (all sectors). (a,b) Global maps of the PCFA-based vulnerability index and the overall ND-GAIN vulnerability (all sectors) for the historical period. (c) Country-level comparison with the 1:1 line (dashed) and OLS fit (red). (d) Bland–Altman plot showing differences centered near zero and contained within ±1.96 SD.

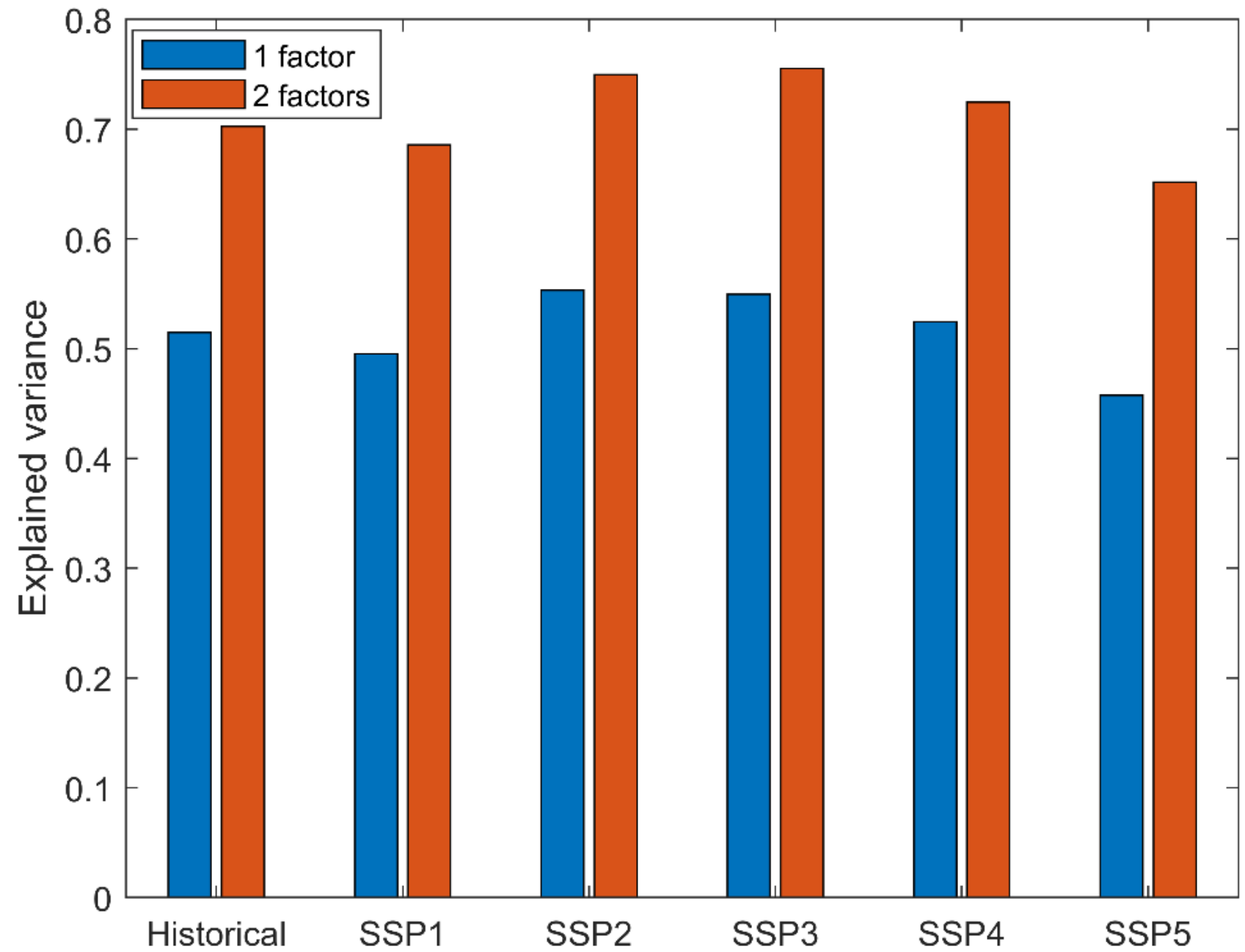

Supplementary Fig. 12 | Variance explained by 1-factor and 2-factor PCFA models. Explained variance for the 1-factor and 2-factor principal component and factor analysis (PCFA) models across the Historical and SSP scenarios. The 1-factor model captures the dominant latent structure of the socioeconomic indicators, while the 2-factor model substantially increases the proportion of variance explained, reflecting additional heterogeneity in the indicator set. The results confirm that although two factors describe more variance, a single factor captures the primary vulnerability gradient that drives cross-country differences.

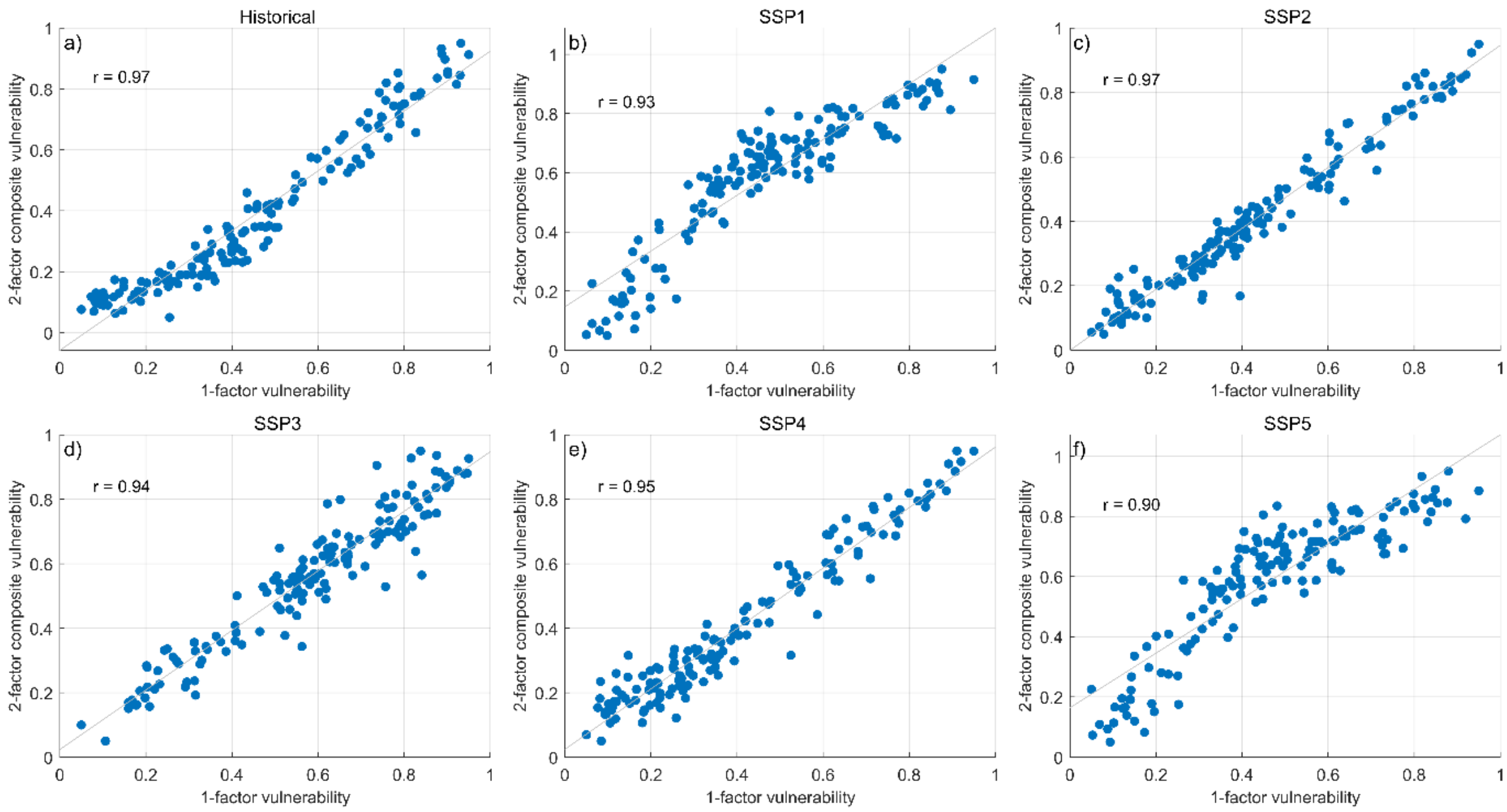

Supplementary Fig. 13 | Comparison between 1-factor and 2-factor PCFA vulnerability indices across scenarios. Panels a–f show the relationship between the 1-factor socioeconomic vulnerability index and the 2-factor composite vulnerability index, both derived using principal component and factor analysis (PCFA), for the historical, SSP1, SSP2, SSP3, SSP4, and SSP5 scenarios, respectively. Each point represents a country. Spearman rank correlation coefficients between the two indices are shown in each panel, and all correlations are statistically significant at the 0.01 level.

Results (Influence on risk):

"To better elucidate the moderating role of vulnerability, we examined the relationship between cropland exposure and resulting risk across different vulnerability quartiles (Fig. 6). Across all hazards and scenarios, the slope between exposure and risk systematically steepens from the lowest (Q1) to the highest (Q4) vulnerability quartile. This effect is most pronounced under SSP3, characterized by regional rivalry and limited adaptation capacity, and weakest under SSP1, where sustainable development reduces vulnerability. Notably, even under moderate exposure conditions, highly vulnerable countries (Q4) exhibit risk levels comparable to or exceeding those of less vulnerable countries under extreme exposure, underscoring the amplifying influence of socioeconomic fragility. The consistent widening gap between

quartiles across future scenarios suggests that disparities in adaptive capacity will increasingly drive the distribution of agricultural risk under climate change."

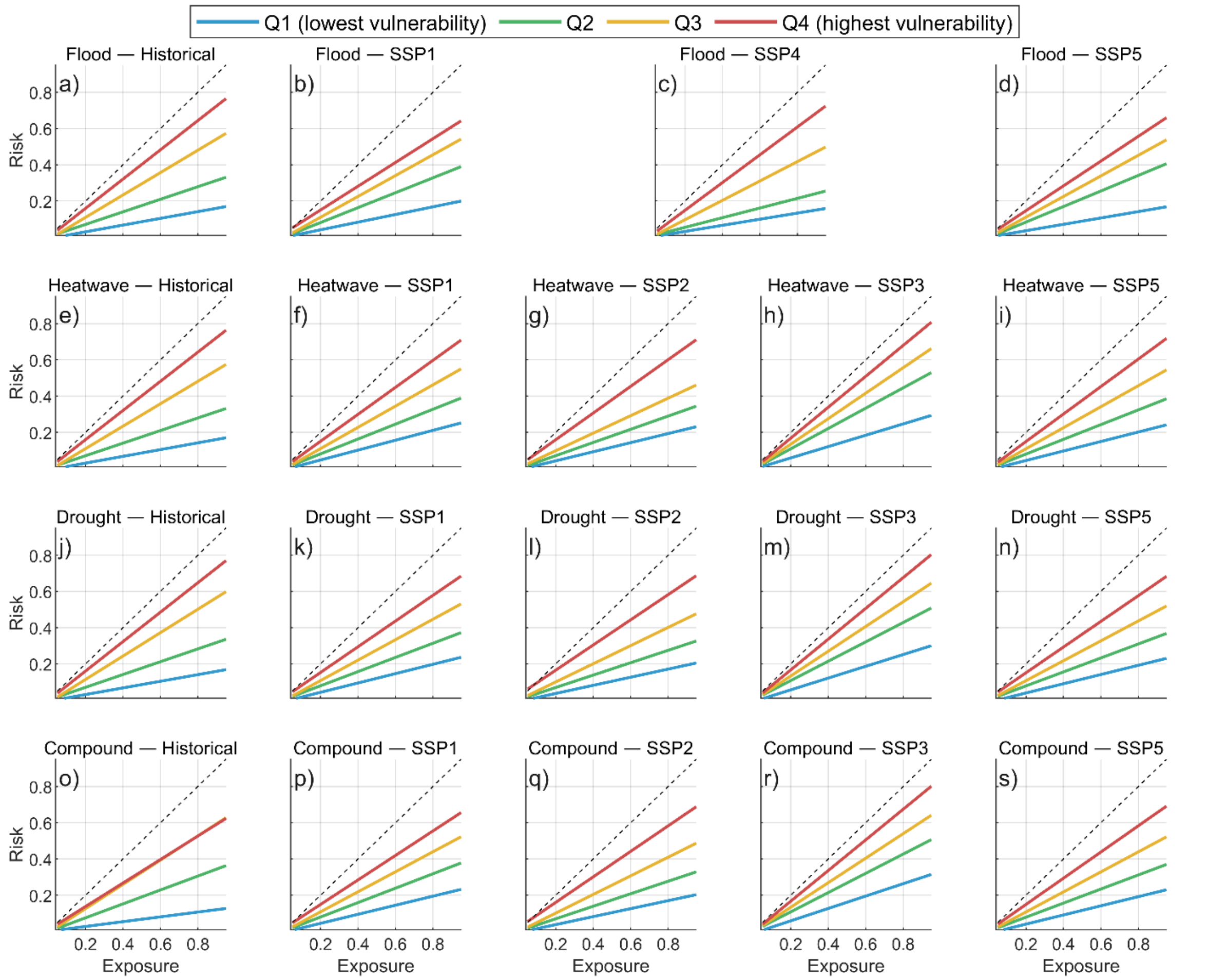

Figure 6 | Exposure–risk relationships by vulnerability quartiles across hazards and SSP scenarios. Each panel shows the relationship between normalized cropland exposure and resulting risk for four vulnerability quartiles (Q1–Q4; colored lines) across 151 countries. Panels a–e depict flood, f–j heatwave, k–o drought, and p–t compound hot–dry events. The dashed line indicates the 1:1 reference where risk = hazard × exposure × vulnerability.

Discussion (Scale and future refinement):

"Similar to our exposure analysis at grid scale (Fig. 1), future research could also explore vulnerability at finer spatial scales to capture subnational disparities and localized adaptation potential. Because

vulnerability is computed at the national scale, substantial within-country heterogeneity in socioeconomic and institutional conditions is not represented. Developing subnational vulnerability indices (e.g., at state/province/district levels) that incorporate region-specific determinants, such as irrigation and drainage access, market connectivity and post-harvest storage capacity, agricultural extension services, and access to credit/insurance, would allow the index to reflect local adaptive capacity rather than national averages. This finer resolution would reveal within-country risk hotspots in major producers (e.g., the Indo-Gangetic Plain in India, the U.S. Midwest/Mississippi Basin, and MATOPIBA in Brazil) and better align risk assessment with jurisdictions that implement adaptation. Such refinement would support targeted, equity-focused planning, by directing resources to places and populations that are both highly exposed and least able to cope. In addition, several vulnerability indicators such as governance-related metrics (e.g., institutional capacity and policy implementation effectiveness) are difficult to quantify systematically and often rely on qualitative assessments, which introduces further uncertainty into the vulnerability estimates."

Reviewer 2 Major comment 3
- Use of the term "compounding": The title is misleading. i) At no point do the authors analyze interactions between the four extremes. Instead, the study presents spatial pattern of risk associated with each climate extreme separately toward the end of the century. ii) Looking specifically at climate extremes in relation to both exposure and adaptive capacity, such analysis is again not demonstrated. The figures (e.g., the network analysis) appear to show only correlations between aggregated country-level indicators and individual climate extremes, rather than interactions among extremes, exposure, and adaptive capacity. No evidence is provided that the combined or synergistic effects of these factors were quantified.

Response

We thank the reviewer for the opportunity to clarify our use of the term "compounding" and the relationships among hazards, exposure, and adaptive capacity in our framework.

(i) Regarding the terminology, we agree that our analysis does not model interactions among all four climate extremes simultaneously. To avoid ambiguity, we revised the title to "Multi-hazard exposure and vulnerability drive rising agricultural risk in global breadbasket regions" and now refer in the text to a multi-hazard risk assessment. We have also clarified that one of the analyzed hazards (compound hot–dry events) explicitly represents a compounding of heat and drought conditions. This event type is included because it is among the most consequential compound extremes affecting agricultural systems globally, and its explicit representation distinguishes our study from previous single-hazard assessments. The revised Methods and Figure captions now clearly define this usage and specify that the "compound" terminology applies only to co-occurring hot–dry conditions rather than to interactions among all four extremes.

(ii) Regarding the relationships among hazards, exposure, and adaptive capacity, we appreciate this comment, which prompted an important addition to the manuscript. To better illustrate how these components interact within our framework following the standard IPCC risk assessment framework, we added a new figure (Fig. 6) showing the relationships among climate hazards, exposure, vulnerability, and resulting risk. This analysis reveals that, for the same level of exposure, risk increases disproportionately where vulnerability is higher. The consistent widening gap between vulnerability quartiles across future scenarios highlights that disparities in adaptive capacity will increasingly govern the distribution of agricultural risk under climate change. We thank the reviewer for this insightful suggestion, which led to an additional layer of interpretation and strengthened the overall contribution of the study.

Finally, we extended our analysis by quantifying risk changes in major breadbasket countries (Argentina, Brazil, China, India, Nigeria, Ukraine and the USA) in a new figure (Supplementary Fig. 12). This

addition illustrates how the combined effects of hazard, exposure and vulnerability translate into heterogeneous multi-hazard risk trajectories across key food-producing regions, and the corresponding results have been incorporated into the revised Results section.

The following explanation of the Fig. 6's results were added to the Results section:

"To better elucidate the moderating role of vulnerability, we examined the relationship between cropland exposure and resulting risk across different vulnerability quartiles (Fig. 6). Across all hazards and scenarios, the slope between exposure and risk systematically steepens from the lowest (Q1) to the highest (Q4) vulnerability quartile. This effect is most pronounced under SSP3, characterized by regional rivalry and limited adaptation capacity, and weakest under SSP1, where sustainable development reduces vulnerability. Notably, even under moderate exposure conditions, highly vulnerable countries (Q4) exhibit risk levels comparable to or exceeding those of less vulnerable countries under extreme exposure, underscoring the amplifying influence of socioeconomic fragility. The consistent widening gap between quartiles across future scenarios suggests that disparities in adaptive capacity will increasingly drive the distribution of agricultural risk under climate change."

The following explanation of the risk changes in major breadbasket countries was added to the Results section:

"Focusing on the major breadbasket countries further illustrates how these dynamics translate into distinct trajectories of agricultural risk (Supplementary Fig. 11). The seven countries exhibit heterogeneous responses across hazards and scenarios. For floods, risk approximately doubles in the USA, Nigeria and India across almost all scenarios, with a moderate increase in Argentina, particularly under SSP2. Heatwaves produce the strongest and most widespread amplification: the USA, Ukraine, Nigeria and India show large increases across all scenarios, with fourfold increases under SSP3, whereas Brazil and Argentina

exhibit pronounced intensification under SSP3 and more moderate increases in the remaining SSPs. China experiences a moderate increase in heatwave-related agricultural risk, most clearly under SSP3. For drought, risk increases consistently across all scenarios in the USA, while in the other breadbasket countries the amplification is concentrated under SSP3. Compound hot–dry events broadly mirror the heatwave response, but with slightly larger risk amplification. Overall, these patterns indicate that the type of climate extreme strongly shapes the magnitude and distribution of future agricultural risk in breadbasket countries, underlining the importance of a multi-hazard perspective in agricultural risk assessment."

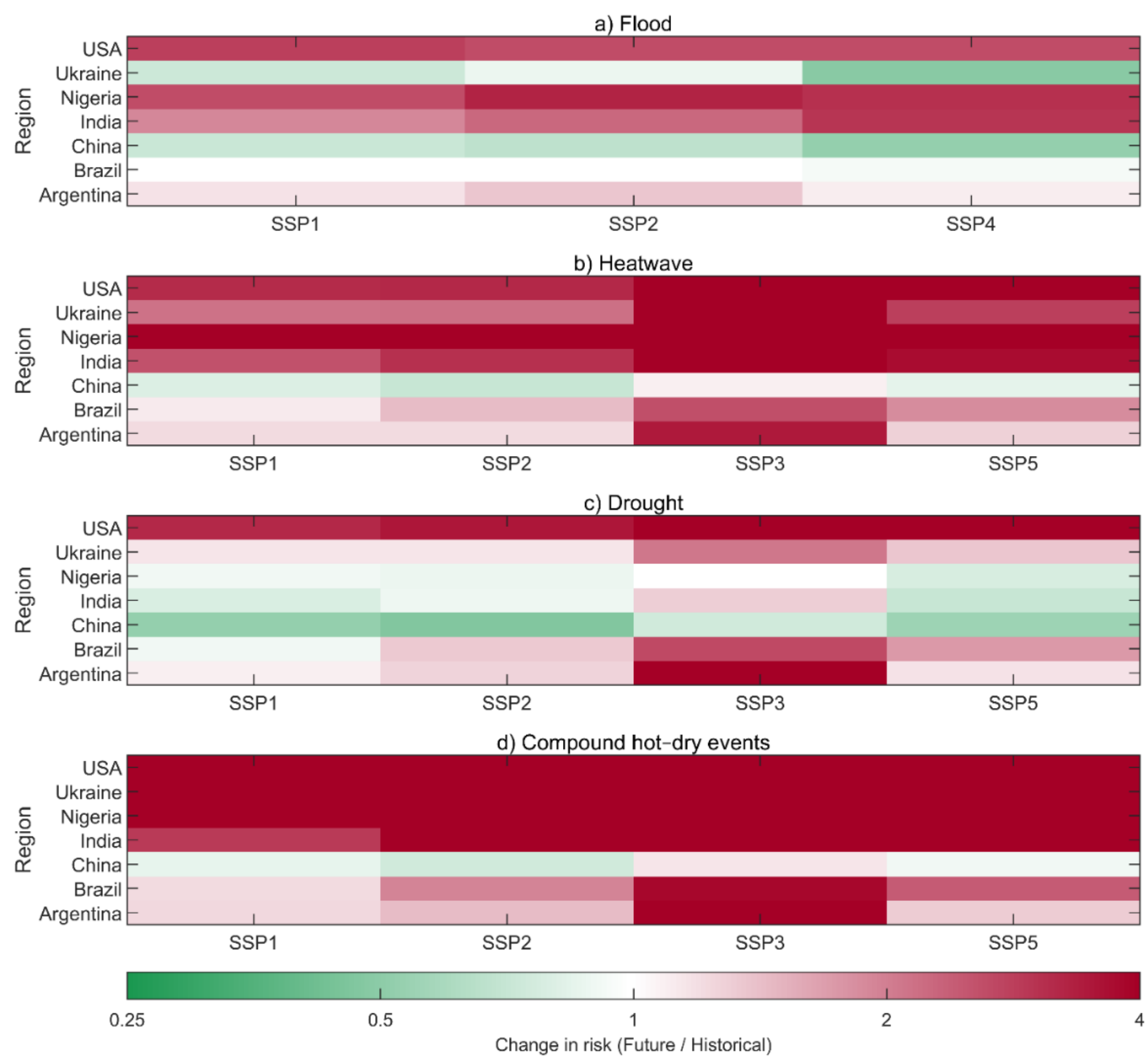

Supplementary Fig. 11 | Changes in agricultural risk across global breadbasket regions under different future scenarios. Heatmaps show the change in agricultural risk (Future / Historical) for a) floods, b) heatwaves, c) droughts and d) compound hot–dry events across seven major breadbasket countries

(Argentina, Brazil, China, India, Nigeria, Ukraine and the USA). Risk is calculated following the IPCC risk assessment framework, as the product of hazard, exposure and vulnerability.

Reviewer 2 Major comment 4
- Risk terminology: The study does not employ an established risk framework such as that of the IPCC. As a result, the terminology is unclear and at times used interchangeably, which weakens the conceptual foundation of the analysis.

Response

In the revised manuscript, we explicitly aligned our terminology and analytical structure with the IPCC risk framework, where risk is defined as the outcome of the interaction between hazard, exposure, and vulnerability. Accordingly:

- The four climate extremes analyzed (heatwaves, droughts, floods, and compound hot–dry events) are now referred to as hazards.
- The projected cropland area affected by these extremes is defined as exposure.
- The socio-demographic and institutional indicators (e.g., governance, education, human development) combined through the PCFA approach represent vulnerability.

We clarified these definitions in the Methods section and used consistent terminology throughout the manuscript to strengthen the conceptual coherence of our framework.

Reviewer 2 Major comment 5
- Discussion: The discussion section is overly general and descriptive, and it lacks quantitative metrics and uncertainty information. This further reinforces the impression that the study does not provide significant novelty.

Response

We agree that a clear discussion of quantitative results and uncertainty is essential for demonstrating the robustness and novelty of the study. We therefore substantially revised the Discussion to ensure it contains clear quantitative metrics, scenario-specific contrasts, and explicit statements about uncertainty, directly addressing the reviewer's concern. The revised text now reports the magnitude of projected exposure increases (e.g., 2–8 million km² for heatwaves and 0.5–3 million km² for droughts in breadbasket regions), differences across scenarios (exposure increases in SSP1–2.6 and SSP2–4.5 being 17–85% smaller than in SSP3–7.0 and SSP5–8.5), and disparities across vulnerability quartiles (high-vulnerability regions showing 1.1–3.3× greater exposure under SSP5–8.5). We also explicitly discuss uncertainty, noting that ensemble inter-model spreads are quantified in the analysis and that ensemble medians consistently indicate increases across hazards, crops, and regions. Finally, the Discussion now clearly articulates the study's novelty by highlighting the emergence of a statistically significant exposure–vulnerability relationship ($p < 0.001$) in future scenarios—a pattern absent historically—demonstrating a structural shift in how agricultural risk evolves under climate change.

Reviewer 2 Specific comment 1
Abstract:<br>In its current form, about 2/3 of the abstract deals with background and methodology. As a result, the novelty of this research is not clear.

Response

We agree that the original abstract placed too much emphasis on background and methodological details, which reduced the visibility of the study's novelty and key findings. To address this, we have fully rewritten the abstract. The revised version now clearly states that global multi-hazard agricultural risk assessments that jointly consider multiple hazards, crop exposure and socioeconomic vulnerability are still missing, and that our study provides a global assessment that fills this gap. The updated abstract reads:

"Climate extremes increasingly threaten global food production, yet global multi-hazard agricultural risk assessments that jointly consider multiple hazards, crop exposure and socioeconomic vulnerability are still missing. This gap limits our ability to identify where climate shocks to croplands are most likely to translate into high agricultural risk. Here we present a global agricultural risk assessment that quantifies the exposure of croplands and ten major crop groups to heatwaves, droughts, floods and compound hot–dry events, and combines this with a vulnerability index constructed from socio-economic and institutional indicators using latent factor modelling. Using ensembles of climate and hydrological models under multiple future scenarios, we show widespread increases in multi-hazard exposure, with the strongest intensification across South Asia, Sub-Saharan Africa and major U.S. and European breadbaskets. A significant relationship between vulnerability and cropland exposure emerges in future scenarios but is absent historically, indicating a growing role for vulnerability in shaping future agricultural risk. These results demonstrate that reliably identifying future agricultural risk hotspots requires jointly accounting for multi-hazard exposure and socioeconomic vulnerability, so that adaptation efforts can be targeted to regions where intensifying extremes coincide with high vulnerability."

Reviewer 2 Specific comment 2
Abstract:<br>The presented results do not clearly demonstrate the importance of integrating multiple hazards and adaptive capacity.

Response

We agree that the original abstract did not clearly convey why integrating multiple hazards and adaptive capacity is essential for identifying agricultural risk hotspots. In the revised abstract, we now explicitly state that a significant relationship between vulnerability and cropland exposure emerges only in future

scenarios and is absent historically, highlighting the growing role of vulnerability in shaping agricultural risk. We also strengthened the concluding sentence to make clear that high-risk hotspots can only be identified when multi-hazard exposure and socioeconomic vulnerability are considered together. The relevant part of the revised abstract now reads:

"Using ensembles of climate and hydrological models under multiple future scenarios, we show widespread increases in multi-hazard exposure, with the strongest intensification across South Asia, Sub-Saharan Africa and major U.S. and European breadbaskets. A significant relationship between vulnerability and cropland exposure emerges in future scenarios but is absent historically, indicating a growing role for vulnerability in shaping future agricultural risk. These results demonstrate that reliably identifying future agricultural risk hotspots requires jointly accounting for multi-hazard exposure and socioeconomic vulnerability, so that adaptation efforts can be targeted to regions where intensifying extremes coincide with high vulnerability."

Reviewer 2 Specific comment 3
Introduction:<br>P1: "The resilience of the global food system hinges on a small number of highly productive agricultural regions, often referred to as breadbaskets…" give concreate evidence. How many tons of crop where produce in these hyper productive regions which account for a low percentage of total cropland.

Response

We acknowledge that the initial sentence lacks concrete numerical evidence. We revised the Introduction to include the best available global estimate:

"*Together, the United States, Argentina, Europe, Russia/Ukraine, China, India, Australia, Indonesia, and Brazil produce 56%, 56%, 73%, and 38% of global wheat, maize, soybean, and rice production, respectively in 2012*[1]*, while occupying about half of the world's cropland*[3,4]."

Reviewer 2 Specific comment 4
Introduction: P1: "which already accounts for nearly…": not very clear; for which period? In addition, I note the inconsistent use of vulnerability and exposure throughout the manuscript where both terms are often used interchangeably.

Response

The studies supporting this statement indeed refer to specific historical periods. Ray et al. (2015) analyzed 1979–2008 and showed that climate variability explains more than 60% of year-to-year yield variability in major global breadbaskets, while Frieler et al. (2017) examined 1971–2012 and similarly found that more than half of global crop-yield variability is attributable to climate variability.

To clarify this in the manuscript, we revised the sentence to:

"*Climate variability already accounts for nearly half of global year-to-year yield fluctuations over the past several decades*[9,10]*, and the intensification of extremes raises the risk of simultaneous production shocks that could destabilize markets and food access*[11-12]."

This wording accurately reflects the multi-decadal periods of the cited studies and removes ambiguity about the relevant time frame. The inconsistent use of vulnerability and exposure noted by the reviewer was also resolved through the broader restructuring and clarification of terminology in the revised Introduction.

Reviewer 2 Specific comment 5
Introduction:<br><br>P2: "…ability to assess risks from these increasingly severe extremes remains limited". I am not convinced that "limited" is the correct term since there are a lot of global studies looking at the impact of climate change on future floods, drought... on population exposure, (crop) damage… Please revise and precise what is/are the limitation(s).

Response

The sentence in question ("…ability to assess risks from these increasingly severe extremes remains limited") no longer appears in the revised Introduction. During the restructuring of the Introduction, we removed this sentence and replaced it with a more precise explanation of the existing gaps, specifically the lack of explicit multi-hazard assessments, the limited treatment of compound extremes, and the frequent omission of socioeconomic vulnerability and adaptive capacity in global crop-risk studies. These revisions clarify the specific limitations in the existing literature and address the reviewer's concern.

Reviewer 2 Specific comment 6
Introduction:<br><br>P2: By "production shocks across multipole breadbaskets", do the author mean simultaneous shocks?

Response

The term did refer to simultaneous production shocks occurring in different major breadbasket regions. To make this explicit and avoid ambiguity, we revised the sentence in the Introduction to: "*Climate variability already accounts for nearly half of global year-to-year yield fluctuations over the past several*

decades[9,10], and the intensification of extremes raises the risk of simultaneous production shocks that could destabilize markets and food access[11-12]."

Reviewer 2 Specific comment 7
Introduction:<br>P2: "Yet, most assessments focus on individual events…": I am not sure I agree with this statement. In this review (https://www.sciencedirect.com/science/article/pii/S2212096321001078#f0010), table 1 provides a relatively view of what has been done. Clearly there are a lot of studies and quickly looking online I could find this study (https://www.nature.com/articles/s43017-023-00491-0) that include both water and temperature stresses and this one (https://www.nature.com/articles/s43017-022-00368-8) including compound heat and moisture extreme. I also notice some crop specific assessments at the global scale such as this article for cotton https://www.sciencedirect.com/science/article/pii/S0378377425000216#fig0005 Overall the statement is too general and it is very unclear what in unknown.

Response

We agree that the original statement in the Introduction was too general, and we revised it to more precisely describe the remaining knowledge gaps. We reviewed the studies cited by the reviewer, including those listed in Table 1 of Shah et al. (2021). Most studies in that table are local or regional studies, as indicated in the "Coping practice" column. Only a small number of large-scale studies exist, and these focused solely on growing-season mean temperatures and critical temperature thresholds during the reproductive stage of crops (Gourdji et al., 2013; Teixeira et al., 2013; Asseng et al., 2014).

Importantly, Lesk et al. (2022) and Rezaei et al. (2023), mentioned by the reviewer, are review papers, not original global multi-hazard assessments. They synthesize existing knowledge but do not provide new global multi-hazard analyses of climate extremes or compound events across multiple crop types. Their own conclusions emphasize that substantial gaps remain in understanding the combined effects of

multiple extreme hazards on global crop production. Likewise, Liu et al. (2025) examines compound drought–heat events but only for a single crop (cotton) and only for the historical period, making it too narrow to address global, multi-crop, multi-hazard futures.

Our literature review shows that no existing global study combines multiple extreme-event types (including compound events) with ten major crop groups and future climate and land-use scenarios. More importantly, socioeconomic vulnerability is entirely absent from these global multi-hazard assessments. To address these gaps, our work presents the first global multi-hazard agricultural risk assessment that integrates exposure to four climate extremes with a multidimensional vulnerability index.

We revised the Introduction accordingly:

"Yet major gaps remain. Most global process-based assessments of agricultural impacts of climate change[14,15,21,23,30,31,32] focus on mean temperature, precipitation, and CO_2 trends or on simple anomalies rather than explicitly quantifying climate extremes (floods, droughts, and heatwaves) that directly drive agricultural losses. In addition, global empirical and statistical analyses typically consider only one hazard at a time[33-35], and future multi-hazard assessments, especially those that include compound events across diverse crop types, are still missing. The absence of such a framework limits understanding of how different hazards and their concurrent or sequential interactions, such as compound hot–dry events, exacerbate agricultural impacts[4,24,36]. Moreover, global crop risk analyses rarely include vulnerability, despite evidence that socioeconomic and institutional conditions strongly modulate impacts[37,38]. As a result, true global agricultural risk hotspots, where high exposure coincides with limited adaptive capacity, remain poorly identified."

Reviewer 2 Specific comment 8
Introduction:<br><br>P2: “cascading impact”: what does that mean concretely?

Response

The phrase “cascading impact” no longer appears in the revised Introduction, as the entire section was restructured during revision.

Reviewer 2 Specific comment 9
Introduction:<br><br>P2: “can trigger non-linear…”; that is also the case for non-compound events. This statement is again too generic. Please revise.

Response

The sentence referring to “can trigger non-linear …” was removed when the Introduction was fully restructured.

Reviewer 2 Specific comment 10
Introduction:<br><br>P2: I find that there is redundant information in the last two paragraphs. In addition these last two paragraph somewhat contradict the previous authors statement that “limiting our understanding of how overlapping climate hazards may jointly disrupt agriculture on a global scale”. As clearly there are a lot of studies.

Response

The Introduction was substantially revised, and the earlier redundancy between the two paragraphs no longer exists. In revising the text, we also clarified the distinction between the existence of studies on individual hazards or specific crop–stress interactions and the absence of a global, multi-hazard assessment that integrates multiple extremes, multiple crop types, future scenarios, and socioeconomic vulnerability. The revised paragraphs now consistently state that, although numerous studies exist, they predominantly address single hazards, individual crop processes, or regional scales, and therefore do not resolve how overlapping climate hazards jointly affect agriculture at the global scale. The introduction was rewritten accordingly to remove redundancy and ensure conceptual consistency.

Reviewer 2 Specific comment 11
Introduction:<br><br>P3: "Yet, few global-scale studies…": Which interacting hazard? Please be specific.

Response

The revised Introduction now explicitly specifies the interacting hazards by referring to compound hot–dry events (co-occurring heatwaves and droughts). The previous vague wording was replaced during restructuring, and the interacting hazard is now clearly identified.

Reviewer 2 Specific comment 12
Introduction:<br><br>P3: "A breadbasket's ability to withstand and recover…" what is the meaning of recover in this context? Do the authors mean that the next growing season will be unimpacted?

Response

The sentence containing the term "recover" was removed when the Introduction was restructured. In the revised version, we describe vulnerability using the IPCC risk framework as suggested by the reviewer.

Reviewer 2 Specific comment 13
Introduction:<br>P3: "by its adaptive capacity…" is what follow the authors definition of adaptive capacity? If so, make that clear.

Response

The sentence containing the phrase "by its adaptive capacity" was removed when the Introduction was restructured. In the revised version, we describe vulnerability consistently within the IPCC risk framework and refer to a vulnerability index that is formally defined in the Methods section.

Reviewer 2 Specific comment 14
Introduction:<br>P3: "Socioeconomic development, governance…" Some studies have started to consider these factors, but they are poorly quantified, provided at coarse spatial and temporal resolutions, and very uncertain. I agree that that should not prevent researchers from including them in their studies, but these limitations must be clearly indicated.

Response

The wording referenced in this comment was removed when the Introduction was restructured. To address the concern raised, we explicitly added the limitations of our vulnerability analysis in the Discussion section. The revised text now reads:

"Similar to our exposure analysis at grid scale (Fig. 1), future research could also explore vulnerability at finer spatial scales to capture subnational disparities and localized adaptation potential. Because vulnerability is computed at the national scale, substantial within-country heterogeneity in socioeconomic and institutional conditions is not represented. Developing subnational vulnerability indices (e.g., at state/province/district levels) that incorporate region-specific determinants, such as irrigation and drainage access, market connectivity and post-harvest storage capacity, agricultural extension services, and access to credit/insurance, would allow the index to reflect local adaptive capacity rather than national averages. This finer resolution would reveal within-country risk hotspots in major producers (e.g., the Indo-Gangetic Plain in India, the U.S. Midwest/Mississippi Basin, and MATOPIBA in Brazil) and better align risk assessment with jurisdictions that implement adaptation. Such refinement would support targeted, equity-focused planning, by directing resources to places and populations that are both highly exposed and least able to cope. In addition, several vulnerability indicators such as governance-related metrics (e.g., institutional capacity and policy implementation effectiveness) are difficult to quantify systematically and often rely on qualitative assessments, which introduces further uncertainty into the vulnerability estimates."

Reviewer 2 Specific comment 15
Introduction:<br><br>P3: "breadbaskets, intermediate, and limited farming zones" Some definition of these categories is needed.

Response

We added a brief clarification in the Introduction that breadbasket, intermediate, and limited farming zones are defined by their cropland fraction, with the precise thresholds specified in the Methods section.

The revised sentence now reads: "*The projected cropland exposure is compared among farming regions (breadbaskets, intermediate, and limited farming zones, defined by their cropland fraction).*"

Reviewer 2 Specific comment 16
Introduction:<br>P3: "Here, we present a global assessment…" In this sentence, the word "vulnerability" is used. Yet the sentence does not mention the "adaptive capacity" although the authors clearly stated that "Critically, exposure does not equate to vulnerability." Altogether this illustrates a core issue where terminology is not used consistently. I strongly suggest sticking with an international framework, for example the one provided by the IPCC: risk arises from the interaction of three factors: hazards, exposure, and vulnerability. This whole paragraph should be rewritten for consistency with the adopted terminology and also to highlight that "exposure does not equate to vulnerability".

Response

In the revised manuscript, we explicitly aligned our terminology and analytical structure with the IPCC risk framework, where risk is defined as the outcome of the interaction between hazard, exposure, and vulnerability. Accordingly:

- The four climate extremes analyzed (heatwaves, droughts, floods, and compound hot–dry events) are now referred to as hazards.
- The projected cropland area affected by these extremes is defined as exposure.
- The socio-demographic and institutional indicators (e.g., governance, education, human development) combined through the PCFA approach represent vulnerability.

We clarified these definitions in the Methods section and used consistent terminology throughout the manuscript to strengthen the conceptual coherence of our framework.

Reviewer 2 Specific comment 17
Results:<br><br>P4: "This expansion is consistent…" expansion of what? If the authors are discussing flood risk the term expansion is not appropriate.

Response

We revised the sentence to: "*The largest increases occur in low-lying, intensively farmed regions of South and Southeast Asia, particularly the Ganges–Brahmaputra and Mekong basins (Fig. 1), consistent with the projected intensification of extreme precipitation in these monsoon-dominated regions*[40]."

Reviewer 2 Specific comment 18
Results:<br><br>P4: "see the largest expansion in cropland affected by persistent extreme heat". Provide some quantitative numbers for these regions.

Response

We added quantitative values to this section.

Reviewer 2 Specific comment 19
Results:<br><br>P4: "robust signal of rising". What does robust mean here?

Response

In climate change studies, and as commonly defined in IPCC reports, robustness typically refers to at least two-thirds of models agreeing on the sign of change. However, to avoid confusion with the term "robustness" used elsewhere in the manuscript for assessing the vulnerability index, we revised the

wording for clarity. The sentence now reads: "*These regions see the largest expansion in cropland affected by persistent extreme heat, reflecting the consistent increase in mean temperatures and heat extremes projected across climate models*[43]*.*"

Reviewer 2 Specific comment 20
Results:<br><br>P5: "striking spatial convergence": what does convergence mean? Do the authors mean that results are similar for both compound hot-dry and drought?

Response

We thank the reviewer for pointing out the ambiguity. By "spatial convergence," we intended to convey that the spatial distribution of compound hot-dry events is shaped by the combined influence of both drought and heatwave exposure, rather than implying that the patterns are identical. To clarify this, we revised the sentence to: "*The spatial pattern of compound hot-dry events reflects the combined influence of both drought and heatwave patterns, with 91% of global croplands showing increasing exposure.*"

Reviewer 2 Specific comment 21
Results:<br><br>P5: "The stark contrast with the lower exposure increases shown under the low-emission scenario SSP1-2.6 (Supplementary Fig. 1) underscores the critical importance of ambitious climate mitigation in safeguarding food production." This is a good illustration of the problems in this section. This is purely descriptive. No numbers are provided at all. Please revise the whole section to: i) provide quantitative metric, ii) and compare the results with existing literature.

Response

We revised this section to (i) include quantitative metrics for the projected changes in exposure and (ii) add comparisons with findings from existing literature.

For example, the mentioned sentence now reads:

"The stark contrast with the lower exposure increases under the low-emission scenario SSP1-2.6, where 54%, 63%, 43%, and 79% of global cropland area are projected to experience increasing exposure to floods, heatwaves, droughts, and compound hot-dry events, respectively (Supplementary Fig. 1), underscores the critical importance of ambitious climate mitigation in safeguarding food production."

We also revised the Results section to explicitly compare our projected changes in cropland exposure with existing studies. In addition, we added a new paragraph in the Discussion where we relate our exposure projections to existing crop-modelling and empirical yield-impact studies, clarifying how the identified increases in exposure are expected to translate into yield losses, especially in major breadbasket regions.

Reviewer 2 Specific comment 22
Results:<br>P8: "experience minimal long-term damage", How can the authors capture damage no data linking crop damage to the four climate extremes are available.

Response

We agree with the reviewer that our analysis does not quantify crop damage, and therefore statements referring to "long-term damage" were not appropriate. It was revised to avoid implying any direct assessment of damage:

“Cropland exposed to extreme events may contribute only modestly to increases in agricultural risk where vulnerability is low, because impacts can be more effectively absorbed and managed. Conversely, even moderate exposure can lead to marked increases in agricultural risk when vulnerability is high.”

Reviewer 2 Specific comment 23
Results:<br><br>P8: “result in severe consequences”, what are we looking at then, damage, yield. Be specific.

Response

It was revised as follows:

“Cropland exposed to extreme events may contribute only modestly to increases in agricultural risk where vulnerability is low, because impacts can be more effectively absorbed and managed. Conversely, even moderate exposure can lead to marked increases in agricultural risk when vulnerability is high.”

Reviewer 2 Specific comment 24
Results:<br><br>P8: “national capacities” The differences in spatial resolution could be an issue. In some nations there are wide differences in some of the factors used to computing adaptive-capacity yet this possible limitation is mention nowhere.

Response

We acknowledge the reviewer’s concern regarding spatial resolution differences in the vulnerability/adaptive-capacity indicators. We have now added a statement in the revised manuscript noting that the vulnerability index is computed at the national scale, which may not fully capture subnational heterogeneity in governance, education, demographics, or development. We also clarify that

this limitation may lead to an underestimation of within-country variability in adaptive capacity, particularly in large or socioeconomically diverse nations. A discussion of this limitation has been added to the Results section and expanded in the Discussion:

"Similar to our exposure analysis at grid scale (Fig. 1), future research could also explore vulnerability at finer spatial scales to capture subnational disparities and localized adaptation potential. Because vulnerability is computed at the national scale, substantial within-country heterogeneity in socioeconomic and institutional conditions is not represented. Developing subnational vulnerability indices (e.g., at state/province/district levels) that incorporate region-specific determinants, such as irrigation and drainage access, market connectivity and post-harvest storage capacity, agricultural extension services, and access to credit/insurance, would allow the index to reflect local adaptive capacity rather than national averages. This finer resolution would reveal within-country risk hotspots in major producers (e.g., the Indo-Gangetic Plain in India, the U.S. Midwest/Mississippi Basin, and MATOPIBA in Brazil) and better align risk assessment with jurisdictions that implement adaptation. Such refinement would support targeted, equity-focused planning, by directing resources to places and populations that are both highly exposed and least able to cope. In addition, several vulnerability indicators such as governance-related metrics (e.g., institutional capacity and policy implementation effectiveness) are difficult to quantify systematically and often rely on qualitative assessments, which introduces further uncertainty into the vulnerability estimates."

Reviewer 2 Specific comment 25
Methods:<br><br>"The outputs from global climate models". Add the number of GCM here.

Response

We added the number of models used. The sentence in the Methods now reads: "*The outputs from 26 global climate models (GCMs) participating in the Coupled Model Intercomparison Project Phase 6 (CMIP6) were used to quantify drought, heatwave, and compound hot-dry events (Supplementary Table 1).*"

Reviewer 2 Specific comment 26
Methods:<br>"For flood analysis…" why did the authors used ISIMIP2 when ISIMIP3 data are now available. This is problematic since the analysis relies on CMIP6 data (as indicated in the paragraph above) and the older CMIP5 data which is what ISIMIP2 relies on. This is critical for future simulations! Where water withdrawal is constant in the future and did, they increase, in line with population growth. If the later this could be an issue since the population (cropland expansion…) projections used in ISIMIP2 are different from those used in the SSPs.

Response

We acknowledge that the next round of ISIMIP, namely ISIMIP3b (based on CMIP6), is underway and that Frieler et al. (2025) describe in detail the scenario set-up and climate-forcing data for groups I and II. However, at the time our analyses were conducted, the full suite of impact-model outputs for ISIMIP3b had not yet been released or evaluated for global consistency. The ISIMIP3b set-up paper (Frieler et al., 2025) itself indicates that additional experiments (group III) are still under preparation.

For this reason, we based our flood analysis on ISIMIP2b, which at that time was the only fully released and quality-controlled global multi-model dataset available for this sector. ISIMIP2b continues to be used in recent studies by even ISIMIP model developers (e.g., Grant et al., 2025) and remains widely adopted by the broader community (Qiu et al., 2025; Woolway et al., 2025; Felikson et al., 2025), underscoring its role as a standard reference framework while ISIMIP3b is being rolled out.

ISIMIP3b simulations will provide an important opportunity to update flood projections with CMIP6-based forcings in future work. However, we do not expect this transition to alter our main conclusions such as the growing role of vulnerability in shaping agricultural risk.

Reviewer 2 Specific comment 27
Methods:<br><br>Adaptive capacity: The input data are disclosed but the section does not provide any insight how these six indicators were combined into a single adaptive capacity…

Response

The procedure for combining them into a single adaptive capacity index are described in the Assessing vulnerability and risk subsection. To ensure that readers are clearly directed to this methodological explanation without duplicating content, we added the following sentence to the end of the Vulnerability data subsection: "*The six indicators were subsequently integrated into a single vulnerability index, as detailed in the assessing vulnerability and risk subsection.*" In addition, the rationale behind selecting these indicators has been moved to the Vulnerability data subsection to consolidate all indicator-related information in one place.

Reviewer 2 Specific comment 28
Methods:<br><br>- Quantification of cropland exposure to climate extremes<br><br>Which simulations specifically were used for the flood analysis: did the authors account for human activity and dams and reservoirs? Why the focus on 50-year flood when many other study use 100-year flood?

Response

For the flood analysis, we used the ISIMIP2b global hydrological model (GHM) simulations, which include human water-use sectors (e.g., irrigation, domestic, industry, livestock) and, for many models, reservoir storage compartments. As documented in the ISIMIP2b Simulation Protocol and extensively summarized by Telteu et al. (2021), the participating GHMs differ in their representation of key hydrological processes, including runoff generation, routing schemes, soil-moisture dynamics, groundwater and surface-water storage, human water use, and reservoir dynamics. Some models include representations of dams, reservoirs, and water withdrawals, while others simulate largely naturalized hydrology. This structural heterogeneity is an important contributor to hydrological model uncertainty.

To ensure transparency without reproducing large technical tables in our paper, we refer readers to Telteu et al. (2021), who provide detailed model-by-model documentation. The following tables in that paper describe the relevant structural features:

- Table 1: Canopy compartment and water flows
- Table 2: Snow and soil compartments and water flows
- Table 3: Groundwater, lake, reservoir, and wetland compartments
- Table 4: River routing schemes
- Table 5:Human water-use sectors
- Tables 6–9: Water-storage compartments, water flows, and key model characteristics

In the revised manuscript, we have expanded the original sentence:

"*Further methodological details on model structure, storage dynamics, and the integration of anthropogenic influences can be found in Telteu et al.*[75]."

to the following:

"*Further methodological details on model structure, including canopy, snow, soil, groundwater, lake, reservoir, wetland and river compartments, human water use sectors, and associated water storage and flow schemes, are provided in Telteu et al.*[74]."

Additionally, we have clarified how this heterogeneity contributes to uncertainty. The revised text now reads:

"Notably, breadbasket regions not only face higher exposure in absolute terms but also exhibit greater inter-model spread, reflecting both intensified and more uncertain risks under future climate conditions. Importantly, the inter-model spread increases from limited to intermediate to breadbasket regions (Fig. 2), indicating higher uncertainty in areas with greater projected exposure. For drought, heatwave, and compound hot–dry events, uncertainty mainly stems from differences in climate inputs across GCMs. For floods, in addition to climate-model differences, uncertainty arises from structural heterogeneity among global hydrological models. This ensemble spread is therefore a key component of projection uncertainty and highlights that regional exposure estimates should be interpreted as probabilistic rather than deterministic."

Regarding the chosen return period of floods, in our analysis, we originally evaluated floods across multiple return periods ranging from 10 to 100 years and found broadly consistent spatial patterns and exposure responses across these thresholds. For brevity, we presented only the 50-year return period in the initial submission. However, we agree that the 100-year flood return period is more commonly used in the literature and enables clearer comparison with previous studies. In the revised manuscript, we therefore recalculated and updated all flood related results and figures using the 100-year return period.

Reviewer 2 Specific comment 29
Methods:<br><br>- Quantification of cropland exposure to climate extremes<br><br>Note that the GHMs do not represent backflow which can be problematic for accurately representing flood dynamics in some flat delta regions. Also critically, note this article (https://agupubs.onlinelibrary.wiley.com/doi/full/10.1029/2024GL108855) that indicate that overestimation of future flood will occur given the current methodology. Overall, many caveats and uncertainty are not discussed at all.

Response

We agree that both global hydrological models (GHMs) and global climate models (GCMs) have structural and methodological limitations that affect simulations of extreme events. GCMs rely heavily on parameterizations of convection, cloud microphysics, precipitation formation, and land–atmosphere exchanges because these processes cannot be explicitly resolved at the coarse spatial resolution required for global simulations. These simplifications directly influence the magnitude, spatial pattern, and temporal variability of extreme precipitation—one of the main drivers of flooding.

Similarly, GHMs include simplified representations of infiltration, groundwater–surface water interactions, floodplain storage, river routing, and reservoir operations, and these schemes vary substantially across models (Telteu et al., 2021). Many GHMs used in ISIMIP rely on coarse global drainage direction maps and apply one-dimensional routing along a single downstream link per grid cell. Such routing schemes do not explicitly represent backflow or tidal/surge-driven hydrodynamics and therefore cannot reproduce complex flood behaviour in flat delta regions (Telteu et al., 2021; Heinicke et al., 2024). In addition, recent evidence shows that statistical approaches used to project extreme events may introduce systematic bias. For example, extreme value analysis applied to short reference periods can overestimate future flood frequency (Tabari, 2021; Zhao et al., 2024).

Together, these limitations—affecting GCMs, GHMs, and statistical methods—introduce uncertainty into flood and extreme-event projections. We have now explicitly addressed these caveats in the revised manuscript (see below).

"Moreover, there are important limitations associated with the global climate (GCM) and hydrological (GHM) simulations used in this study. Both modelling systems rely on simplified representations of key physical processes because fully resolving atmospheric and hydrological dynamics at fine spatial scales is computationally infeasible at the global level. In GCMs, processes such as convection, cloud microphysics, boundary-layer turbulence, and land–atmosphere coupling are parameterized, which affects the simulation of extreme precipitation[72,73]. GHMs likewise parameterize infiltration, groundwater–surface water interactions, floodplain storage, river routing, reservoir operations, and water withdrawals, with substantial structural differences across models[74]. Many global hydrological models, including several used in ISIMIP, employ coarse global drainage direction maps and one-dimensional routing along a single downstream link per grid cell. These schemes do not explicitly represent backflow or tidal and surge-driven hydrodynamics and are therefore not suited to resolving flood processes in flat delta regions[74,75]. Moreover, statistical methods used to estimate extreme events may introduce bias: both extreme value analysis[40] and short-baseline frequency estimation can lead to overestimation of future flood occurrence[76]. Combined with the coarse resolution of GCMs and GHMs, the structural heterogeneity among models, and methodological biases, these factors contribute to uncertainty in global hazard projections."

Reviewer 2 Specific comment 30
Methods:<br><br>- Quantification of cropland exposure to climate extremes<br><br>“Following the calculation of event probabilities, cropland exposure was determined for each grid cell by multiplying the cropland proportion”: is this a commonly applied methodology, reference?

Response

Defining exposure as the product of asset extent and hazard probability is standard practice in agricultural exposure studies (e.g., Mondal et al., 2024; Gu et al., 2025). To clarify this in the manuscript, we revised the sentence to: “*Following the calculation of event probabilities, cropland exposure was determined for each grid cell by multiplying the cropland proportion by the event probabilities, consistent with standard exposure definitions used in agricultural exposure studies*[107,108]*.*”

Reviewer 2 Specific comment 31
Methods:<br><br>- Quantification of cropland exposure to climate extremes<br><br>“Limited farming regions have cropland coverage of less than 10%, intermediate farming regions have...” is the analysis based on 0.5-degree grids? Is this classification/definition widely used?

Response

The analysis is indeed based on 0.5° × 0.5° grid cells, which is the spatial resolution of the cropland data and exposure calculations used throughout the study. Breadbasket regions have been defined in various ways in previous studies. These studies typically define breadbaskets based on present-day global production of specific crops (Gaupp et al., 2019, 2020; Caparas et al., 2021; Zhang et al., 2024), and mostly at coarse national or sub-national scales (Anderson et al., 2023). Such definitions neglect (i) potential

future changes in breadbasket locations driven by land-use change and climate, and (ii) smaller-scale heterogeneity within countries or large regions.

Because our aim is to analyze cropland exposure at a finer spatial scale, we define farming regions at the grid-cell level rather than at national or subnational administrative units. In addition, to account for possible future changes in cropland extent, and thus in farming regions (Supplementary Fig. 2), we define farming regions separately for the historical period and for each future SSP scenario.

Similar to the spatial extent of breadbasket regions in earlier work, there is no universally accepted standard threshold for defining breadbaskets. Previous studies have, for example, used thresholds such as:

- regions contributing ≥10% of simulated yields for a given crop within a continent (Franke et al., 2022),
- regions producing ≥70% of present-day global production of a given crop (Zhang et al., 2024), or
- sets of countries that collectively contribute ≥90% of global maize or wheat production (Anderson et al., 2023).

In our original analysis, we defined three farming regions, limited farming, intermediate farming, and breadbasket regions, based on the proportion of total cropland in each 0.5° × 0.5° grid cell (Supplementary Fig. 2). For each historical and future scenario, we used:

- Limited farming regions: cropland coverage < 10%
- Intermediate farming regions: cropland coverage between 10% and 30%
- Breadbasket regions: cropland coverage > 30%

This dynamic grid-based definition allowed us to consistently track changes in the spatial distribution of intensive cropping areas over time and across scenarios. Our historical breadbasket regions, defined in this way, correspond well to known major crop-producing states and provinces of key agricultural countries based on FAOSTAT (FAO, 2022) and official subnational production statistics. Examples include

Shandong, Hebei, Henan, Jiangsu, and Anhui in China; Córdoba in Argentina; Paraná and São Paulo in Brazil; Uttar Pradesh, Punjab, Maharashtra, and Karnataka in India; and Wisconsin, Minnesota, and Illinois in the United States.

In the revised manuscript, we adopted a more systematic, distribution-based classification using quartiles of the cropland proportion to define farming clusters:

- Limited farming regions: grid cells with cropland proportion below the first quartile (Q1, i.e. below the 25th percentile).
- Lower and upper intermediate farming regions: grid cells with cropland proportion between the first quartile (Q1) and the third quartile (Q3, i.e. between the 25th and 75th percentiles). These together form the intermediate farming regions in our study.
- Breadbasket regions: grid cells with cropland proportion above the third quartile (Q3, i.e. above the 75th percentile).

This quartile-based classification yields very similar patterns and only slightly changes the quantitative results compared to the original fixed-threshold approach. However, it is more systematic and aligns with common practice in socioeconomic vulnerability analysis, such as the World Bank's classification of countries by income into quantile-based groups (Fantom and Serajuddin, 2016), the use of income or wealth quintiles in poverty and inequality assessments (Alizadeh et al., 2022), and the aggregation of regional vulnerability or exposure into terciles or quartiles in risk-index studies.

The following sub-section was added to the revised paper:

"Definition of farming regions

There is no universally accepted standard method for defining farming regions, and breadbasket regions have been defined in various ways in previous studies, typically based on present-day global production of

specific crops[1,12,13,102] and mostly at coarse national or sub-national scales[103]. Because our aim is to analyze cropland exposure at a finer spatial scale, we define farming regions at the 0.5° × 0.5° grid-cell level, consistent with the spatial resolution of the cropland dataset used in this study. To account for possible future changes in cropland extent, and thus in farming regions, we identify farming regions separately for the historical period and for each future SSP scenario, so that the classification reflects changing land use patterns (Supplementary Fig. 2).

We use the statistical distribution of cropland proportion to distinguish three types of farming regions. Grid cells with cropland proportion below the first quartile (Q1) are classified as limited farming regions and represent the least intensively cultivated 25% of cropland areas. Intermediate farming regions comprise grid cells with cropland proportion between the first and third quartiles (Q1–Q3), capturing moderately cultivated landscapes. Breadbasket regions are defined as grid cells with cropland proportion above the third quartile (Q3), corresponding to the most intensively cropped 25% of areas.

Historical breadbasket regions delineated using this approach correspond closely to known major crop producing areas based on FAOSTAT[2] and subnational production statistics, such as Shandong, Hebei, Henan, Jiangsu, and Anhui in China; Uttar Pradesh, Punjab, Maharashtra, and Karnataka in India; Paraná and São Paulo in Brazil; Córdoba in Argentina; and Wisconsin, Minnesota, and Illinois in the United States[1].”

Reviewer 2 Specific comment 32
Methods:<br><br>- Quantification of cropland exposure to climate extremes<br><br>In this section, it is not clear us how the results for different GCM and GHM were aggregated (mean median…) and if/how uncertainty was quantified.

Response

We agree that mentioning the ensemble median only in the figure captions was not sufficient. We have therefore revised the Methods section to clarify how results from different GCM–GHM combinations are aggregated and how uncertainty is quantified. Specifically, we now state that all GCM–GHM combinations are treated as equally plausible ensemble members, that the main maps show the multi-model ensemble median change in exposure, and that uncertainty is represented by the spread across all models. We also clarify that the boxplots and ± values reported in the text describe the inter-model spread.

The Methods section now includes the following clarification:

"To aggregate results across climate models, each GCM is treated as an equally plausible ensemble member. All central estimates reported in the main text and figures correspond to the multi-model ensemble median, unless stated otherwise. Projection uncertainty is quantified as the inter-model spread, computed for each metric across the full ensemble. Specifically, we report uncertainty as the 95th–5th percentile range in Figs. 1 and 2, and as the standard deviation across models in Fig. 3."

"As with the CMIP6 ensemble, all GCM–GHM combinations were equally weighted, and hydrological model uncertainty is represented by the inter-model spread (Figs. 1-3)."

Reviewer 2 Specific comment 33
Methods:<br><br>- Assessing adaptive capacity<br><br>Adaptive capacity was assessed…": redundant with Adaptive capacity data section.

Response

The detailed description of the six indicators is now confined to the Vulnerability data subsection. In the Assessing vulnerability and risk subsection, we no longer repeat the indicators and instead refer only to a composite vulnerability index derived from these six socio-demographic and institutional indicators, followed by a detailed explanation of the PCFA method used to combine them.

Reviewer 2 Specific comment 34
Methods:<br>- Assessing adaptive capacity<br>What is not clear about all input data is their spatial resolution. As a result, the methodology is also very unclear.

Response

All six indicators used to quantify adaptive capacity are available at the country level. This information is already included in the Vulnerability data subsection. To make the spatial resolution more explicit, we clarified the opening sentence to state that country-level data were used.

Reviewer 2 Specific comment 35
Methods:<br>- Assessing adaptive capacity<br>"To improve the accuracy of the factor solution.." Accuracy about what? There is no ground truth here so how do the authors can assess whereas the derived indicator is "accurate" or not?

Response

We agree that the term "accuracy" is inappropriate here. The intention was to describe improvements in the statistical fit and numerical stability of the factor solution. To avoid any misunderstanding, we replaced "improve the accuracy of the factor solution" with "*improve the numerical stability and fit of the factor solution*".

Reviewer 2 Specific comment 36
Figures:<br><br>Fig. 1: is there any quantification of uncertainty?

Response

In the revised manuscript, we now quantify and visualize the uncertainty in Fig. 1 using inset boxplots. These boxplots summarize the spread of projected changes across all hydrological and climate models for each farming region (limited, intermediate, and breadbasket): the boxes show the interquartile range of model outcomes and the whiskers represent the full ensemble range. This provides a clear and explicit representation of inter-model uncertainty in future exposure estimates. In addition, Fig. 2 represents the uncertainty in the magnitude of the projected changes in total cropland exposure, and Fig. 3 addresses model uncertainty in crop-specific exposure projections by showing the inter-model spread of exposure changes for major crop types.

Reviewer 2 Specific comment 37
Figures:<br><br>Fig. 2: as I noticed previously. The ISIMIP2 data does not use CMPI6. As a result, the x axis of the flood panel can be misleading as ssp1-2.6 in flood is not truly comparable to ssp1-2.6 for the other extreme

events which do rely on CMIP6. Also make clear which land use scenario is presented here. If this is the default scenario, then the x-axis of the panel for flood should be RCP2.6, RCP4.6 and RCP8.5.

Response

Figure 2 presents cropland exposure, which we define as the product of a climate-driven hazard and cropland proportion. For floods, the hazard component is taken from the ISIMIP2b simulations, which are forced by CMIP5 RCP scenarios, whereas the cropland component follows the AIM-SSP/RCP land-use projections (e.g. SSP1–RCP2.6, SSP2–RCP4.5). A fully explicit scenario label for flood exposure would therefore take the form "RCP2.6 + SSP1–RCP2.6", but such notation would be cumbersome and potentially more confusing in the figure. For this reason, we retained the abbreviated SSP labels in the flood panel to denote the combined exposure scenario. However, we agree that without explanation this could be misinterpreted, and we have now clarified this in both the figure caption and the Methods.

For heatwaves, droughts and compound hot–dry events, both the hazard and cropland components are derived from CMIP6/SSP scenarios, and the SSP labels directly correspond to the underlying forcing for these hazards.

We have implemented the following clarifications:

Sentence added to the Figure 2 caption:

"Cropland exposure was defined as the product of a climate-driven hazard and cropland proportion, and changes are calculated as differences between the late twentieth century and the late twenty-first century. For floods, the hazard is taken from RCP-forced ISIMIP2b simulations and combined with SSP-based cropland, whereas for the other extremes both hazard and land use are SSP-based."

Sentences added to the Methods:

"To assess cropland exposure to climate extremes, we obtained total cropland proportions for historical (2005) and future (2085) periods from the AIM-SSP/RCP Gridded Emissions and Land-use dataset at a 0.5° × 0.5° resolution[92] for five scenario combinations: SSP1–RCP2.6, SSP2–RCP4.5, SSP3–RCP6.0, SSP4–RCP4.5 and SSP5–RCP6.0."

"For floods, the event probabilities are taken from ISIMIP2b simulations forced by RCP scenarios and are combined with the SSP-based cropland fractions described above to obtain flood exposure. For heatwaves, droughts and compound hot–dry events, both event probabilities and cropland are derived from SSP scenarios."

Reviewer 2 Specific comment 38
Figures:<br>Fig. 4: the scale change constantly across extreme events but also farming regions. As a result, it is impossible to compare the panels with each other.

Response

In the original Fig. 4, we used different radial scales for the individual panels to improve the visibility of within-panel patterns and added numerical values on each circle. We agree that this choice made visual comparison across panels difficult. In the revised version, we therefore use a common radial scale (-2 — 4) for all panels to ensure full comparability.

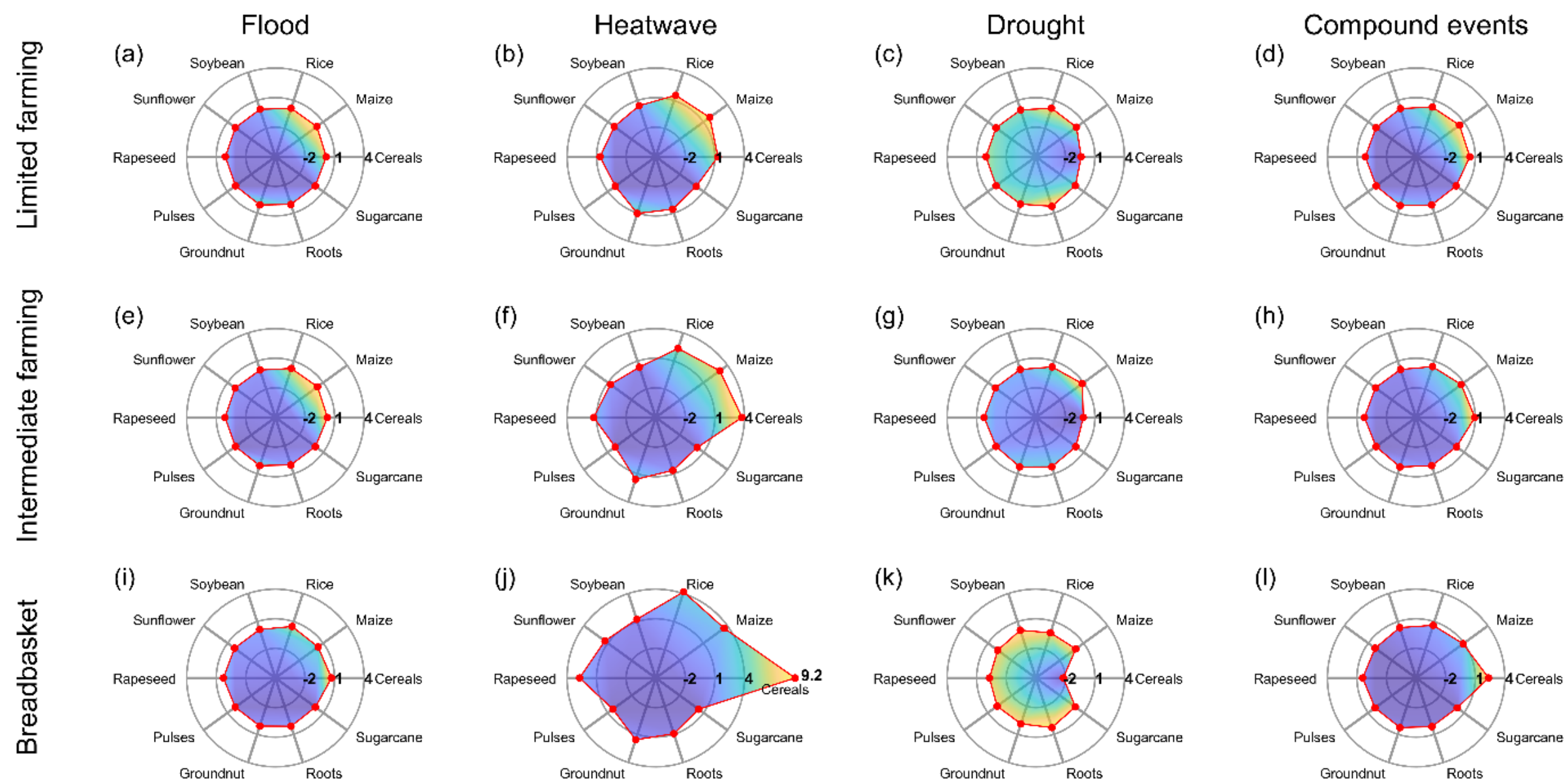

Fig. 4 | Comparison of projected changes in global cropland exposure of different crops to climate extremes across farming regions for SSP5-8.5. Shown are the differences in cropland exposure (×10^5 km^2) between the late 20th century and the late 21st century for various crops, represented as values along the axes of plots. The concentric circles indicate increasing levels of exposure change. Results for other scenarios are provided in Supplementary Figs. 3-6.

Reviewer 2 Specific comment 39

Figures:

Fig. 6: I do not understand the point of this figure; it doesn't add much scientific insight beyond what a correlation matrix would show and ultimately is more of a visualization exercise than an analysis. It seems that the authors aggregated metric at the country level to compute correlation (would be nice to add the explanation…). What is the meaning of the arrow, correlation is symmetric? To me the finding also appears to be inconsistent particularly when looking at SSP3 and SSP4 versus the other graphs.

Response

We agree that the original Fig. 6 did not substantially advance the objectives of the study and that its correlation-based design could be misleading. In the revised manuscript, we removed this figure and replaced it with a new figure that explicitly stratifies the exposure–risk relationship by vulnerability quartiles across all hazards and scenarios, thereby providing a clearer and more directly relevant analysis.

References

Alizadeh, M. R., Abatzoglou, J. T., Adamowski, J. F., Prestemon, J. P., Chittoori, B., Akbari Asanjan, A., & Sadegh, M. (2022). Increasing heat-stress inequality in a warming climate. Earth's Future, 10(2), e2021EF002488.

Anderson, W., Baethgen, W., Capitanio, F., Ciais, P., Cook, B. I., da Cunha, C. G., ... & You, L. (2023). Climate variability and simultaneous breadbasket yield shocks as observed in long-term yield records. Agricultural and Forest Meteorology, 331, 109321.

Asseng, S., Ewert, F., Martre, P., Rötter, R. P., Lobell, D. B., Cammarano, D., ... & Zhu, Y. (2015). Rising temperatures reduce global wheat production. Nature climate change, 5(2), 143-147.

Caparas, M., Zobel, Z., Castanho, A. D., & Schwalm, C. R. (2021). Increasing risks of crop failure and water scarcity in global breadbaskets by 2030. Environmental Research Letters, 16(10), 104013.

Fantom, N. J., & Serajuddin, U. (2016). The World Bank's classification of countries by income. World bank policy research working paper, (7528), available at: https://documents1.worldbank.org/curated/en/408581467988942234/pdf/WPS7528.pdf

FAO. 2022. Land use statistics and indicators. Global, regional and country trends – 2000–2020. FAOSTAT Analytical Brief, no. 48. Rome. https://doi.org/10.4060/cc0963en

Felikson, D., Rounce, D. R., Fasullo, J., Rodriguez, A., Adhikari, S., Buzzanga, B., ... & Weathers, M. (2025). Progress and future directions in constraining uncertainties in sea-level projections using observations. Nature Climate Change, 1-13.

Franke, J. A., Müller, C., Minoli, S., Elliott, J., Folberth, C., Gardner, C., ... & Moyer, E. J. (2022). Agricultural breadbaskets shift poleward given adaptive farmer behavior under climate change. Global Change Biology, 28(1), 167-181.

Frieler, K. et al. Understanding the weather signal in national crop-yield variability. Earth's Future 5, 605-616 (2017).

Frieler, K., Lange, S., Schewe, J., Mengel, M., Treu, S., Otto, C., ... & Lengaigne, M. (2025). Scenario set-up and the new CMIP6-based climate-related forcings provided within the third round of the Inter-Sectoral Model Intercomparison Project (ISIMIP3b, group I and II). EGUsphere, 2025, 1-70.

Gaupp, F., Hall, J., Hochrainer-Stigler, S. & Dadson, S. Changing risks of simultaneous global breadbasket failure. Nat. Clim. Change 10, 54-57 (2020).

Gaupp, F., Hall, J., Mitchell, D. & Dadson, S. Increasing risks of multiple breadbasket failure under 1.5 and 2 C global warming. Agric. Syst. 175, 34-45 (2019).

Gourdji, S. M., Sibley, A. M., & Lobell, D. B. (2013). Global crop exposure to critical high temperatures in the reproductive period: historical trends and future projections. Environmental Research Letters, 8(2), 024041.

Grant, L., Vanderkelen, I., Gudmundsson, L., Fischer, E., Seneviratne, S. I., & Thiery, W. (2025). Global emergence of unprecedented lifetime exposure to climate extremes. Nature, 641(8062), 374-379.

Gu, L., Schumacher, D. L., Fischer, E. M., Slater, L. J., Yin, J., Sippel, S., ... & Knutti, R. (2025). Flash drought impacts on global ecosystems amplified by extreme heat. Nature Geoscience, 1-7.

Liu, S., Zhang, W., Shi, T., Li, T., Li, H., Zhou, G., ... & Ma, X. (2025). Increasing exposure of cotton growing areas to compound drought and heat events in a warming climate. Agricultural Water Management, 308, 109307.

Mondal, S. K., Su, B., Huang, J., Zhai, J., Wang, G., Kundzewicz, Z. W., ... & Jiang, T. (2024). Climate change will aggravate South Asian cropland exposure to drought by the middle of 21st century. Earth's Future, 12(5), e2023EF003872.

Qiu, Y., Chen, J., Chen, D., Thiery, W., Mercado-Bettín, D., Xiong, L., ... & Woolway, R. I. (2025). Enhanced heating effect of lakes under global warming. Nature communications, 16(1), 1-11.

Ray, D. K., Gerber, J. S., MacDonald, G. K. & West, P. C. Climate variation explains a third of global crop yield variability. Nat. Commun. 6, 5989 (2015).

Shah, H., Hellegers, P., & Siderius, C. (2021). Climate risk to agriculture: A synthesis to define different types of critical moments. Climate Risk Management, 34, 100378.

Teixeira, E. I., Fischer, G., Van Velthuizen, H., Walter, C., & Ewert, F. (2013). Global hot-spots of heat stress on agricultural crops due to climate change. Agricultural and forest Meteorology, 170, 206-215.

Woolway, R. I., Kayastha, M. B., Tong, Y., Feng, L., Shi, H., & Xue, P. (2025). Subsurface heatwaves in lakes. Nature Climate Change, 1-6.

Zhang, H., Yu, X., Qiao, F., & Li, C. (2024). Increasing concurrent exposure of global breadbaskets to reproductive heat extremes. Environmental Research Letters, 19(8), 084046.

3 Reviewer 3

Reviewer 3 General Comments
This is an valuable and timely works that brings together climate extremes and adaptive capacity in a global agricultural vulnerability framework. The integration of multiple hazards with socio-institutional indicators is valuable, and the use of the existing model is innovative. The manuscript is clear and well organized, but several areas need improvement to strengthen its clarity, methodological robustness, and policy relevance. Paper can be accepted after addressing following major revision comments.

Response

We thank the reviewer for the constructive and encouraging evaluation of our manuscript. We carefully addressed all revision points to improve the paper's clarity, methodological transparency, and policy relevance. Specifically, we:

1. **Clarified the research gap and novelty** by explicitly stating how our study advances beyond previous assessments of compound extremes and adaptive capacity, emphasizing our integrated, multi-hazard, and risk-based framework.
2. **Strengthened methodological transparency** by detailing how model uncertainty, bias correction, and ensemble weighting were handled for both CMIP6 and ISIMIP datasets, and by incorporating explicit quantification of inter-model spread and uncertainty in the Results.
3. **Expanded the adaptive capacity framework discussion** to validate our vulnerability index against the established ND-GAIN dataset to confirm robustness.
4. **Addressed scale limitations** by explicitly discussing the need for subnational vulnerability assessments that account for within-country heterogeneity and locally relevant adaptation capacity.

5. **Provided a more balanced assessment of intermediate farming regions**, quantifying their increasing exposure and discussing their potential role as stabilizing buffers in the global food system.
6. **Substantially expanded the policy implications section** to include concrete, region-specific adaptation options for key hotspots such as South Asia, Sub-Saharan Africa, and North America, focusing on crop diversification, irrigation efficiency, early-warning systems, and trade mechanisms.

Reviewer 3 Comment 1
The research gap could be stated more explicitly. The introduction provides useful context but does not clearly explain how this study advances beyond earlier work on compound extremes or adaptive capacity assessments.

Response

We clarified the research gap and our advancement in the revised Introduction. Numerous global studies examine gradual climate change—typically mean temperature, precipitation, and CO_2—using process-based or statistical crop models, which primarily assess yield sensitivity to mean climate forcing. In contrast, our study adopts a risk-based, multi-hazard framework centered on climate extremes (heatwaves, droughts, floods, and compound hot–dry events) which are among the most critical yet least represented drivers of agricultural disruption and risk amplification.

Importantly, our analysis is underpinned by new, high-resolution projections for four climate hazards, derived from ensembles of climate and hydrological models under multiple future scenarios. Producing these multi-hazard projections in a consistent global framework required hazard-specific statistical modeling and validation, yielding a unique, internally consistent suite that captures both single and compound extremes. To our knowledge, this is the first global effort to jointly quantify future exposure of

cropland and crop types to multiple hazards—including compound events—within a unified modeling framework.

Beyond exposure mapping, our approach explicitly integrates the socioeconomic dimension of risk. Rather than modeling physiological yield responses, we quantify where and how much cropland and crop types will be exposed to multiple hazards and combine this with vulnerability and adaptive capacity indicators derived via PCFA (principal-component factor analysis). This integration identifies true risk hotspots—locations where high physical exposure intersects with limited coping capacity—an aspect not addressed by prior studies.

Thus, our contribution is both methodological and conceptual. Methodologically, we generate global multi-hazard projections (including compound extremes) from a large climate–hydrology ensemble, providing a new community dataset; and we develop a PCFA-based vulnerability index that we integrate with exposure to quantify agricultural risk. Conceptually, we bridge physical hazard exposure with socioeconomic vulnerability, moving beyond impact quantification toward a risk-informed resilience assessment that directly advances earlier work on compound extremes and adaptive capacity.

Finally, we revised the Introduction to state these gaps explicitly and to highlight the novelty of our study as the first global risk assessment of agricultural crops that integrates multi-hazard exposure and socioeconomic vulnerability.

"Over the past decade, major advances have been made in quantifying the impacts of gradual warming and shifting climate patterns on agricultural productivity. Process-based crop models have been used to project yields under different global warming levels[14], rising atmospheric CO_2[15], and regional climate conditions[16-20]. Global multi-model ensembles now estimate mean yield responses for major crops under various emission scenarios[21]. Similarly, empirical approaches link historical crop yields to observed climate anomalies[1,5,22-26] or explore adaptation measures such as adjusting sowing dates and cultivar maturity

classes[27], as well as breeding advances[28,29]. These studies have provided valuable insights into long-term changes in crop productivity under gradual climate forcing.

Yet major gaps remain. Most global process-based assessments of agricultural impacts of climate change[14,15,21,23,30,31,32] focus on mean temperature, precipitation, and CO_2 trends or on simple anomalies rather than explicitly quantifying climate extremes (floods, droughts, and heatwaves) that directly drive agricultural losses. In addition, global empirical and statistical analyses typically consider only one hazard at a time[33-35], and future multi-hazard assessments, especially those that include compound events across diverse crop types, are still missing. The absence of such a framework limits understanding of how different hazards and their concurrent or sequential interactions, such as compound hot–dry events, exacerbate agricultural impacts[4,24,36]. Moreover, global crop risk analyses rarely include vulnerability, despite evidence that socioeconomic and institutional conditions strongly modulate impacts[37,38]. As a result, true global agricultural risk hotspots, where high exposure coincides with limited adaptive capacity, remain poorly identified."

Reviewer 3 Comment 2

The choice of six adaptive capacity indicators is reasonable, but the rationale for excluding other relevant factors such as infrastructure, inequality, or access to technology needs to be discussed. Even a brief sensitivity test (not compulsory but recommended) or acknowledgement of this limitation would increase confidence in the index.

Response

We agree that infrastructure, inequality, and access to technology are critical dimensions of adaptive capacity. In this study, we focused on six indicators for which future projections under SSPs 1–5 are available, selected for their demonstrated ability to capture the socio-demographic and institutional dimensions of vulnerability.

Within this framework, the Governance Indicator (GI) serves as a proxy for institutional strength and the enabling environment, indirectly reflecting a country's capacity to provide and maintain infrastructure and to facilitate technology development and diffusion. Likewise, the Human Development Index (HDI) captures broader socioeconomic development that is closely associated with inequality patterns, even though it does not explicitly quantify distributional inequality.

To address the reviewer's comment regarding robustness, we benchmarked our PCFA-based vulnerability index against the comprehensive Notre Dame Global Adaptation Initiative (ND-GAIN) index, which includes 36 indicators but is only available for the historical period. The results show strong agreement ($r = 0.881$; $p < 0.001$), indicating that our selected indicators successfully capture the core structural aspects of vulnerability and adaptive capacity.

The validation of the PCFA-based index for both the food sector and all sectors is presented in Supplementary Figures 9 and 10, and described in the Results section as follows:

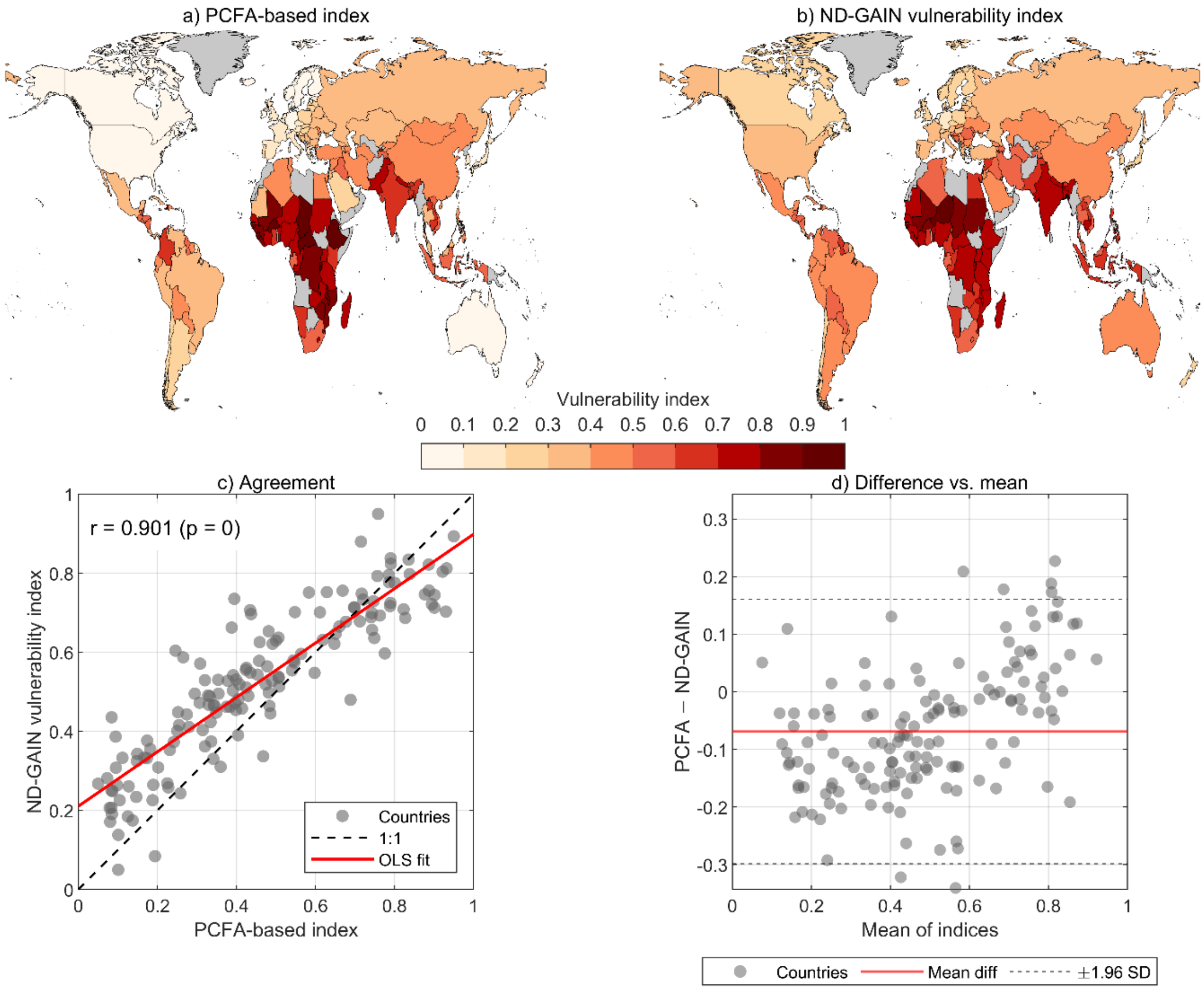

Supplementary Fig. 9 | Validation of the PCFA-based vulnerability index for the food sector. (a,b) Global maps of the PCFA-based vulnerability index (this study) and the ND-GAIN food-sector vulnerability for the historical period. (c) Country-level comparison with the 1:1 line (dashed) and ordinary least squares (OLS) fit (red). (d) Bland–Altman plot showing differences (PCFA − ND-GAIN) centered near zero and contained within ±1.96 SD.

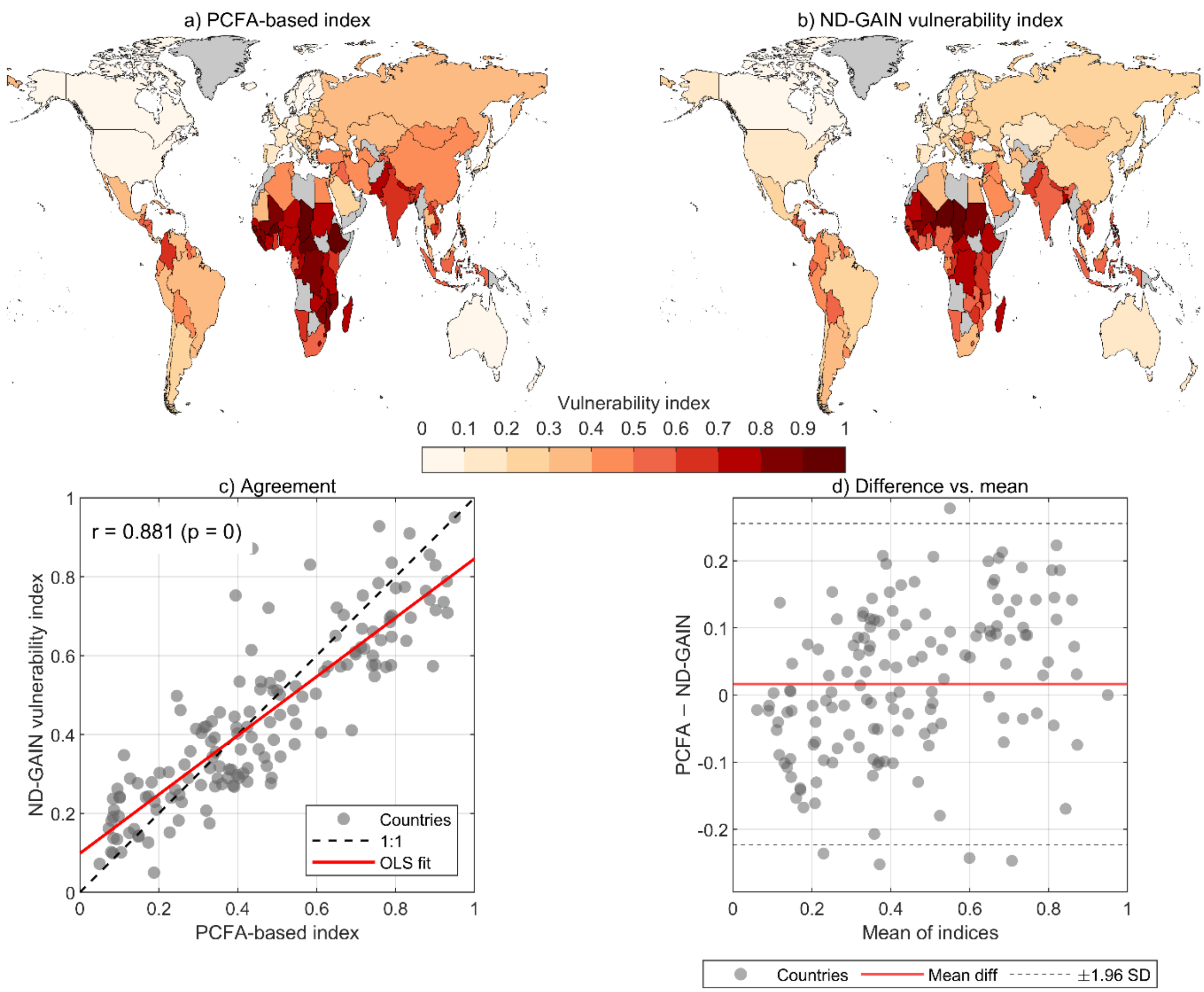

Supplementary Fig. 10 | Validation of the PCFA-based vulnerability index against overall ND-GAIN vulnerability (all sectors). (a,b) Global maps of the PCFA-based vulnerability index and the overall ND-GAIN vulnerability (all sectors) for the historical period. (c) Country-level comparison with the 1:1 line (dashed) and OLS fit (red). (d) Bland–Altman plot showing differences centered near zero and contained within ±1.96 SD.

"To assess the robustness, we benchmarked the PCFA vulnerability index against the Notre Dame Global Adaptation Initiative (ND-GAIN), a widely used and comprehensive composite of socioeconomic vulnerability that lacks future projections. Compared with the ND-GAIN food-sector vulnerability, spatial patterns align across countries (Supplementary Fig. S9a,b) and a strong linear association emerges in the

country-by-country comparison ($r = 0.881$, $p < 0.001$), with most countries near the 1:1 line (Supplementary Fig. S9c). A Bland–Altman analysis shows differences cantered near zero and bounded within ±1.96 SD with no trend versus the mean (Supplementary Fig. S9d), indicating no systematic bias. To test generalizability beyond the food sector, we compare the PCFA index with overall ND-GAIN vulnerability (36 indicators across six sectors); agreement remains high in both spatial patterns and cross-country correlations (Supplementary Fig. S10), with similarly small, unbiased differences in Bland–Altman space. Consistency with both sector-specific and overall ND-GAIN indices indicates that the PCFA index captures core structural dimensions of socioeconomic vulnerability and supports its use for future projections."

In the Methods, we added the following justification text:

"We selected these indicators because future projections are available under SSPs 1–5 and they robustly capture socio-demographic and institutional dimensions of vulnerability, as detailed below."

We also added the following explanation about the validation of our vulnerability index in the Methods section:

"To evaluate the robustness and representativeness of the PCFA-based vulnerability index, we compared it against the Notre Dame Global Adaptation Initiative (ND-GAIN)[114] vulnerability index, which is among the most comprehensive and widely used indicators of socioeconomic vulnerability. The ND-GAIN index is available at the country level for the historical period and quantifies vulnerability across six sectors (food, water, health, ecosystem services, human habitat, and infrastructure) based on 36 indicators. For consistency, we compared the PCFA index first with the ND-GAIN food-sector vulnerability, which is most directly related to agricultural and food system sensitivity, and subsequently with the overall ND-GAIN vulnerability encompassing all sectors. Validation was conducted through correlation and regression

analyses to assess agreement, and (ii) Bland–Altman analysis to evaluate systematic bias and limits of agreement."

Reviewer 3 Comment 3
The handling of model uncertainty deserves more attention. The paper draws on outputs from CMIP6 and ISIMIP, yet it is not clear how inter-model spread, bias correction, or weighting influenced the results. More transparency on these points would improve robustness.

Response

We thank the reviewer for highlighting the need for transparency to improve interpretation and reproducibility. We now clarified how model uncertainty, bias correction, and ensemble weighting were handled throughout the study. Specifically, CMIP6 projections were used without additional bias correction, while ISIMIP hydrological simulations were produced within the ISIMIP framework by forcing global hydrological models with bias-corrected CMIP5 GCMs, using the trend-preserving correction to the EWEMBI dataset (Hempel et al., 2013; Lange, 2019). To ensure consistency and avoid overemphasizing any single model, all CMIP6 and ISIMIP ensemble members were assigned equal weight, and central estimates are reported as multi-model ensemble medians. Model uncertainty is explicitly quantified as the inter-model spread (95th–5th percentile), shown in Fig. 2 for exposure and in Fig. 3 as the standard deviation across models.

We added the following clarifications to the manuscript to enhance transparency and clearly communicate the range of plausible outcomes:

In Methods (model weighting and uncertainty):

"To aggregate results across climate models, each GCM is treated as an equally plausible ensemble member. All central estimates reported in the main text and figures correspond to the multi-model ensemble median, unless stated otherwise. Projection uncertainty is quantified as the inter-model spread, computed for each metric across the full ensemble. Specifically, we report uncertainty as the 95th–5th percentile range in Figs. 1 and 2, and as the standard deviation across models in Fig. 3."

"As with the CMIP6 ensemble, all GCM–GHM combinations were equally weighted, and hydrological model uncertainty is represented by the inter-model spread (Figs. 1-3)."

In Methods (bias correction):

"Before being used to force the GHMs, the raw GCM outputs were bias-corrected to the EWEMBI reference dataset (EartH2Observe, WFDEI, and ERA-Interim data Merged and Bias-corrected for ISIMIP[90]) using a trend-preserving method[91]."

In Results (uncertainty in Fig. 2):

"Notably, breadbasket regions not only face higher exposure in absolute terms but also exhibit greater inter-model spread, reflecting both intensified and more uncertain risks under future climate conditions. Importantly, the inter-model spread increases from limited to intermediate to breadbasket regions (Fig. 2), indicating higher uncertainty in areas with greater projected exposure. For drought, heatwave, and compound hot–dry events, uncertainty mainly stems from differences in climate inputs across GCMs. For floods, in addition to climate-model differences, uncertainty arises from structural heterogeneity among global hydrological models. This ensemble spread is therefore a key component of projection uncertainty

and highlights that regional exposure estimates should be interpreted as probabilistic rather than deterministic."

In Results (uncertainty in Fig. 3):

"In terms of spatial coverage (Fig. 1), a large fraction of breadbasket regions is projected to experience increasing cropland exposure: 59% (±7% uncertainty across models), 94% (±0.6%), 53% (±8%), and 98% (±1.1%) of breadbasket croplands show rising exposure to floods, heatwaves, droughts, and compound hot-dry events, respectively."

"In terms of spatial coverage (Fig. 1), 62% (±4% uncertainty across models), 86% (±0.4%), 57% (±6%), and 91% (±1.8%) of intermediate farming regions are projected to experience increasing cropland exposure to floods, heatwaves, droughts, and compound hot-dry events, respectively."

"The inter-model spread (error bars) indicates moderate to large uncertainty, particularly for heatwave and flood exposure.

Heatwaves drive the largest increases, with cereal exposure expanding by 0.7–1.6 million km² (±0.2 million km² uncertainty) depending on the scenario (Fig. 3). Among individual crops, maize and rice are consistently the most exposed to climate extremes, particularly to heatwaves, 0.4-0.9 million km² (±0.1 million km² uncertainty), followed by compound hot-dry events, floods, and droughts. However, sugarcane stands out with higher exposure to drought. Most crop types follow a similar exposure ranking: heatwaves > compound events > floods > droughts."

Reviewer 3 Comment 4

The analysis is performed at 0.5° spatial resolution, which inevitably masks sub-national heterogeneity within breadbasket regions. This limitation should be acknowledged more explicitly, especially given the importance of local-level adaptation.

Response

We fully agree. In response, we expanded the Discussion section to emphasize the importance of finer-scale vulnerability assessments that capture subnational disparities and localized adaptation potential. The revised text now reads:

"Similar to our exposure analysis at grid scale (Fig. 1), future research could also explore vulnerability at finer spatial scales to capture subnational disparities and localized adaptation potential. Because vulnerability is computed at the national scale, substantial within-country heterogeneity in socioeconomic and institutional conditions is not represented. Developing subnational vulnerability indices (e.g., at state/province/district levels) that incorporate region-specific determinants, such as irrigation and drainage access, market connectivity and post-harvest storage capacity, agricultural extension services, and access to credit/insurance, would allow the index to reflect local adaptive capacity rather than national averages. This finer resolution would reveal within-country risk hotspots in major producers (e.g., the Indo-Gangetic Plain in India, the U.S. Midwest/Mississippi Basin, and MATOPIBA in Brazil) and better align risk assessment with jurisdictions that implement adaptation. Such refinement would support targeted, equity-focused planning, by directing resources to places and populations that are both highly exposed and least able to cope. In addition, several vulnerability indicators such as governance-related metrics (e.g., institutional capacity and policy implementation effectiveness) are difficult to quantify systematically and often rely on qualitative assessments, which introduces further uncertainty into the vulnerability estimates."

Reviewer 3 Comment 5
The adaptive capacity index, while methodologically interesting, would benefit from additional robustness comparison. For example, a comparison with existing vulnerability indices would help validate the results.

Response

We thank the reviewer for this insightful comment. Following the suggestion, we performed a validation analysis to evaluate the robustness of our adaptive capacity index. Specifically, we compared the adaptive capacity component of our PCFA-based vulnerability index with the Notre Dame Global Adaptation Initiative (ND-GAIN) vulnerability index, one of the most comprehensive and widely used global indicators of socioeconomic vulnerability. The comparison shows high consistency in both spatial patterns and country-level correlations, confirming that our representation of adaptive capacity aligns with established assessments. The results are now presented in Supplementary Figs. S9–S10 (as shown above), and the following text has been added to the Results and Methods sections, respectively:

Addition to Results:

"To assess the robustness, we benchmarked the PCFA vulnerability index against the Notre Dame Global Adaptation Initiative (ND-GAIN), a widely used and comprehensive composite of socioeconomic vulnerability that lacks future projections. Compared with the ND-GAIN food-sector vulnerability, spatial patterns align across countries (Supplementary Fig. S9a,b) and a strong linear association emerges in the country-by-country comparison ($r = 0.881$, $p < 0.001$), with most countries near the 1:1 line (Supplementary Fig. S9c). A Bland–Altman analysis shows differences cantered near zero and bounded within ±1.96 SD with no trend versus the mean (Supplementary Fig. S9d), indicating no systematic bias. To test generalizability beyond the food sector, we compare the PCFA index with overall ND-GAIN vulnerability (36 indicators across six sectors); agreement remains high in both spatial patterns and cross-country

correlations (Supplementary Fig. S10), with similarly small, unbiased differences in Bland–Altman space. Consistency with both sector-specific and overall ND-GAIN indices indicates that the PCFA index captures core structural dimensions of socioeconomic vulnerability and supports its use for future projections."

Addition to methods:

"To evaluate the robustness and representativeness of the PCFA-based vulnerability index, we compared it against the Notre Dame Global Adaptation Initiative (ND-GAIN)[114] vulnerability index, which is among the most comprehensive and widely used indicators of socioeconomic vulnerability. The ND-GAIN index is available at the country level for the historical period and quantifies vulnerability across six sectors (food, water, health, ecosystem services, human habitat, and infrastructure) based on 36 indicators. For consistency, we compared the PCFA index first with the ND-GAIN food-sector vulnerability, which is most directly related to agricultural and food system sensitivity, and subsequently with the overall ND-GAIN vulnerability encompassing all sectors. Validation was conducted through correlation and regression analyses to assess agreement, and (ii) Bland–Altman analysis to evaluate systematic bias and limits of agreement."

Reviewer 3 Comment 6
The focus is largely on breadbasket regions, yet intermediate regions also show substantial increases in exposure. These areas may play a buffering role in global supply chains, and a more balanced discussion of their significance would be good.

Response

We revised the text in the "Regional disparities in cropland exposure" section to include a more balanced discussion of intermediate farming regions. Specifically, we now quantify their increases in cropland

exposure across all hazards and scenarios and emphasize their potential role in stabilizing global food supply networks. The revised paragraph reads as follows:

"Although exposure in breadbasket regions is highest, intermediate farming regions also exhibit substantial increases in cropland exposure across all extremes and scenarios. These regions often act as transitional production zones and potential buffers within global food supply networks, and may therefore play a critical role in absorbing future production shocks and maintaining food system stability. For example, under SSP5-8.5, median exposure in intermediate regions increases by 2.5 million km² for heatwaves, 0.6 million km² for drought, 0.5 million km² for compound hot–dry events, and 0.3 million km² for floods, representing roughly 34–40% of the corresponding increases observed in breadbasket regions. In terms of spatial coverage (Fig. 1), 62% (±4% uncertainty across models), 86% (±0.4%), 57% (±6%), and 91% (±1.8%) of intermediate farming regions are projected to experience increasing cropland exposure to floods, heatwaves, droughts, and compound hot-dry events, respectively."

Reviewer 3 Comment 7
The policy implications remain quite general. The discussion would be stronger if it offered concrete examples of adaptation options tailored to the identified hotspots, such as crop diversification, irrigation efficiency, or regional trade measures.

Response

We agree and substantially expanded the policy discussion to include region-specific and actionable adaptation options aligned with the identified exposure and vulnerability hotspots. The revised section now provides concrete examples of measures relevant to key breadbasket regions:

"Governments, development agencies, and multilateral institutions should therefore prioritize context-specific and inclusive adaptation actions in the regional hotspots identified by emerging risk assessments. In South Asia, investments in irrigation efficiency, groundwater recharge, and drought-resilient crop varieties can buffer yield losses associated with recurrent heat and water stress. Expanding access to climate-smart irrigation and precision agriculture technologies will also enhance long-term water-use sustainability. In Sub-Saharan Africa, where limited institutional capacity exacerbates exposure, priority should be given to crop diversification, small-scale water storage, and farmer-led insurance and credit systems. These approaches strengthen resilience by stabilizing production, improving local food access, and supporting smallholder autonomy under climate uncertainty. In the United States, exposure is driven primarily by climate change rather than structural adaptive deficits. Adaptation should therefore focus on regional trade adjustments, modernization of irrigation infrastructure, and flexible water-allocation policies aligned with state and federal adaptation plans.

Across all breadbasket regions, accelerating early-warning systems, agricultural extension services, and climate-informed decision-support tools will be essential to translate adaptive capacity into tangible risk reduction. Evidence shows that early-warning investments yield "triple dividends"[78] by saving lives, reducing economic losses, and strengthening long-term resilience particularly when linked to community-based and digital advisory platforms. Crucially, coordinating adaptation across multi-breadbasket systems via interoperable early-warning and market information platforms, contingency trade arrangements, and regional risk-pooling is essential to limit cascading food insecurity risks and dampen systemic shocks to global supply chains."

Thank you for the opportunity to review this study entitled "*Compounding effects of high exposure and low adaptive capacity intensify climate risks in breadbasket regions*".

Unfortunately, I have several major concerns regarding this study:

- **Novelty**: the originality of this work is unclear. The authors state that "Although significant progress has been made in understanding the impacts of long-term mean climate change, the ability to assess risks from these increasingly severe extremes remains limited". However, this claim is overly general and not entirely accurate, as numerous global studies have already examined crop-climate interactions. The present study appears to simply overlay projections of floods, heatwaves, droughts, and compound hot-dry events onto cropland. It is not evident how this relatively simplistic approach advances beyond existing studies that employ crop models and account for CO_2 fertilization, temperature, water availability, management practices, and crop growth stages.
- **Adaptive capacity**: The only potential innovative aspect is the inclusion of "adaptive capacity", which I do not recall being incorporated in previous studies. While promising, its derivation, rationale, validation, and influence on crop risk are insufficiently explained. The PCFA method, used to derive adaptive capacity, is a standard method in climate vulnerability and risk assessments but has inherent limitations: subjectivity in choosing indicators (here 6), number of factors, and interpretation none of which are discussed in the article. As currently framed, it is unclear how this metric meaningfully alters the risk assessment. In principle, crop modeling could reveal cases where regions with high exposure (i.e., to floods or droughts) might reduce risk due to low vulnerability, but such insights are absent here. Fig. 6, the only figure showing "adaptive-capacity", reduce adaptive capacity (in the form of the 6 indicators) and risk into country-level aggregates, then correlate them. That is a very coarse representation, and it doesn't really capture how adaptive capacity modulates crop risk under extremes.
- **Use of the term "compounding"**: The title is misleading. i) At no point do the authors analyze interactions between the four extremes. Instead, the study presents spatial pattern of risk associated with each climate extreme separately toward the end of the century. ii) Looking specifically at climate extremes in relation to both exposure and adaptive capacity, such analysis is again not demonstrated. The figures (e.g., the network analysis) appear to show only correlations between aggregated country-level indicators and individual climate extremes, rather than interactions among extremes, exposure, and adaptive capacity. No evidence is provided that the combined or synergistic effects of these factors were quantified.
- **Risk terminology:** The study does not employ an established risk framework such as that of the IPCC. As a result, the terminology is unclear and at times used interchangeably, which weakens the conceptual foundation of the analysis.
- **Discussion**: The discussion section is overly general and descriptive, and it lacks quantitative metrics and uncertainty information. This further reinforces the impression that the study does not provide significant novelty.

As a result, I cannot recommend this study for publication.

Specific comments

Abstract

- In its current form, about 2/3 of the abstract deals with background and methodology. As a result, the novelty of this research is not clear.
- The presented results do not clearly demonstrate the importance of integrating multiple hazards and adaptive capacity

Introduction

P1: "The resilience of the global food system hinges on a small number of highly productive agricultural regions, often referred to as breadbaskets…" give concreate evidence. How many tons of crop where produce in these hyper productive regions which account for a low percentage of total cropland.

P1: "which already accounts for nearly…": not very clear; for which period? In addition, I note the inconsistent use of vulnerability and exposure throughout the manuscript where both terms are often used interchangeably.

P2: "…ability to assess risks from these increasingly severe extremes remains limited". I am not convinced that "limited" is the correct term since there are a lot of global studies looking at the impact of climate change on future floods, drought... on population exposure, (crop) damage… Please revise and precise what is/are the limitation(s).

P2: By "production shocks across multipole breadbaskets", do the author mean <u>simultaneous</u> shocks?

P2: "Yet, most assessments focus on individual events…": I am not sure I agree with this statement. In this review (https://www.sciencedirect.com/science/article/pii/S2212096321001078#f0010), table 1 provides a relatively view of what has been done. Clearly there are a lot of studies and quickly looking online I could find this study (https://www.nature.com/articles/s43017-023-00491-0) that include both water and temperature stresses and this one (https://www.nature.com/articles/s43017-022-00368-8) including compound heat and moisture extreme. I also notice some crop specific assessments at the global scale such as this article for cotton https://www.sciencedirect.com/science/article/pii/S0378377425000216#fig0005 Overall the statement is too general and it is very unclear what in unknown.

P2: "cascading impact": what does that mean concretely?

P2: "can trigger non-linear…"; that is also the case for non-compound events. This statement is again too generic. Please revise.

P2: I find that there is redundant information in the last two paragraphs. In addition these last two paragraph somewhat contradict the previous authors statement that "limiting our understanding of how overlapping climate hazards may jointly disrupt agriculture on a global scale". As clearly there are a lot of studies.

P3: "Yet, few global-scale studies…": Which interacting hazard? Please be specific.

P3: "A breadbasket's ability to withstand and recover…" what is the meaning of recover in this context? Do the authors mean that the next growing season will be unimpacted?

P3: "by its adaptive capacity…" is what follow the authors definition of adaptive capacity? If so, make that clear.

P3: "Socioeconomic development, governance…" Some studies have started to consider these factors, but they are poorly quantified, provided at coarse spatial and temporal resolutions, and very uncertain. I agree that that should not prevent researchers from including them in their studies, but these limitations must be clearly indicated.

P3: "breadbaskets, intermediate, and limited farming zones" Some definition of these categories is needed.

P3: "Here, we present a global assessment…" In this sentence, the word "vulnerability" is used. Yet the sentence does not mention the "adaptive capacity" although the authors clearly stated that "Critically, exposure does not equate to vulnerability." Altogether this illustrates a core issue where terminology is not used consistently. I strongly suggest sticking with an international framework, for example the one provided by the IPCC: risk arises from the interaction of three factors: hazards, exposure, and vulnerability. This whole paragraph should be rewritten for consistency with the adopted terminology and also to highlight that "exposure does not equate to vulnerability".

Results

P4: "This expansion is consistent…" expansion of what? If the authors are discussing flood risk the term expansion is not appropriate.

P4: "see the largest expansion in cropland affected by persistent extreme heat". Provide some quantitative numbers for these regions.

P4: "robust signal of rising". What does robust mean here?

P5: "striking spatial convergence": what does convergence mean? Do the authors mean that results are similar for both compound hot-dry and drought?

P5: "The stark contrast with the lower exposure increases shown under the low-emission scenario SSP1-2.6 (Supplementary Fig. 1) underscores the critical importance of ambitious climate mitigation in safeguarding food production." This is a good illustration of the problems in this section. This is purely descriptive. No numbers are provided at all. Please revise the whole section to: i) provide quantitative metric, ii) and compare the results with existing literature.

P8: "experience minimal long-term damage", How can the authors capture damage no data linking crop damage to the four climate extremes are available.

P8: "result in severe consequences", what are we looking at then, damage, yield. Be specific.

P8: "national capacities" The differences in spatial resolution could be an issue. In some nations there are wide differences in some of the factors used to computing adaptive-capacity yet this possible limitation is mention nowhere.

Methods

"The outputs from global climate models". Add the number of GCM here.

“For flood analysis…” why did the authors used ISIMIP2 when ISIMIP3 data are now available. This is problematic since the analysis relies on CMIP6 data (as indicated in the paragraph above) and the older CMIP5 data which is what ISIMIP2 relies on. This is critical for future simulations! Where water withdrawal is constant in the future and did, they increase, in line with population growth. If the later this could be an issue since the population (cropland expansion…) projections used in ISIMIP2 are different from those used in the SSPs.

Adaptive capacity: The input data are disclosed but the section does not provide any insight how these six indicators were combined into a single adaptive capacity…

Quantification of cropland exposure to climate extremes

Which simulations specifically were used for the flood analysis: did the authors account for human activity and dams and reservoirs? Why the focus on 50-year flood when many other study use 100-year flood?

Note that the GHMs do not represent backflow which can be problematic for accurately representing flood dynamics in some flat delta regions. Also critically, note this article (https://agupubs.onlinelibrary.wiley.com/doi/full/10.1029/2024GL108855) that indicate that overestimation of future flood will occur given the current methodology. Overall, many caveats and uncertainty are not discussed at all.

“Following the calculation of event probabilities, cropland exposure was determined for each grid cell by multiplying the cropland proportion”: is this a commonly applied methodology, reference?

“Limited farming regions have cropland coverage of less than 10%, intermediate farming regions have...” is the analysis based on 0.5-degree grids? Is this classification/definition widely used?

In this section, it is not clear us how the results for different GCM and GHM were aggregated (mean median…) and if/how uncertainty was quantified.

Assessing adaptive capacity

“Adaptive capacity was assessed…”: redundant with **Adaptive capacity data** section.

What is not clear about all input data is their spatial resolution. As a result, the methodology is also very unclear.

“To improve the accuracy of the factor solution..” Accuracy about what? There is no ground truth here so how do the authors can assess whereas the derived indicator is “accurate” or not?

Figures

Fig. 1: is there any quantification of uncertainty?

Fig. 2: as I noticed previously. The ISIMIP2 data does not use CMPI6. As a result, the x axis of the flood panel can be misleading as ssp1-2.6 in flood is not truly comparable to ssp1-2.6 for the other

extreme events which do rely on CMIP6. Also make clear which land use scenario is presented here. If this is the default scenario, then the x-axis of the panel for flood should be RCP2.6, RCP4.6 and RCP8.5

Fig. 4: the scale change constantly across extreme events but also farming regions. As a result, it is impossible to compare the panels with each other.

Fig. 6: I do not understand the point of this figure; it doesn't add much scientific insight beyond what a correlation matrix would show and ultimately is more of a visualization exercise than an analysis. It seems that the authors aggregated metric at the country level to compute correlation (would be nice to add the explanation…). What is the meaning of the arrow, correlation is symmetric? To me the finding also appears to be inconsistent particularly when looking at SSP3 and SSP4 versus the other graphs.
